# Integrative genomic analyses in adipocytes implicate DNA methylation in human obesity and diabetes

**DOI:** 10.1038/s41467-023-38439-z

**Published:** 2023-05-15

**Authors:** Liam McAllan, Damir Baranasic, Sergio Villicaña, Scarlett Brown, Weihua Zhang, Benjamin Lehne, Marco Adamo, Andrew Jenkinson, Mohamed Elkalaawy, Borzoueh Mohammadi, Majid Hashemi, Nadia Fernandes, Nathalie Lambie, Richard Williams, Colette Christiansen, Youwen Yang, Liudmila Zudina, Vasiliki Lagou, Sili Tan, Juan Castillo-Fernandez, James W. D. King, Richie Soong, Paul Elliott, James Scott, Inga Prokopenko, Inês Cebola, Marie Loh, Boris Lenhard, Rachel L. Batterham, Jordana T. Bell, John C. Chambers, Jaspal S. Kooner, William R. Scott

**Affiliations:** 1 Institute of Clinical Sciences, Faculty of Medicine, Imperial College London, London, W12 0NN UK; 2 MRC London Institute of Medical Sciences, London, W12 0NN UK; 3 Department of Twin Research and Genetic Epidemiology, King’s College London, London, UK; 4 Department of Epidemiology and Biostatistics, School of Public Health, Imperial College London, London, W2 1PG UK; 5 Department of Cardiology, Ealing Hospital, London North West University Healthcare NHS Trust, Middlesex, UB1 3HW UK; 6 UCLH Bariatric Centre for Weight Loss, Weight Management and Metabolic and Endocrine Surgery, University College London Hospitals, Ground Floor West Wing, 250 Euston Road, London, NW1 2PG UK; 7 Imperial BRC Genomics Facility, Faculty of Medicine, Imperial College London, London, W12 0NN UK; 8 School of Mathematics and Statistics, Faculty of Science, Technology, Engineering and Mathematics, The Open University, Milton Keynes, UK; 9 School of Cardiovascular and Metabolic Medicine and Sciences, James Black Centre, King’s College London British Heart Foundation Centre of Excellence, 125 Coldharbour Lane, London, SE5 9NU UK; 10 Department of Clinical & Experimental Medicine, University of Surrey, Guildford, UK; 11 Department of Microbiology and Immunology, Laboratory of Adaptive Immunity, KU Leuven, Leuven, Belgium; 12 VIB-KU Leuven Center for Brain and Disease Research, Leuven, Belgium; 13 Cancer Science Institute of Singapore, National University of Singapore, Singapore, Singapore; 14 Department of Pathology, National University Hospital, Singapore, Singapore; 15 MRC Centre for Environment and Health, School of Public Health, Imperial College London, London, UK; 16 National Institute for Health Research Biomedical Research Centre, Imperial College London, London, UK; 17 National Heart and Lung Institute, Imperial College London, London, W12 0NN UK; 18 Imperial College Healthcare NHS Trust, London, W12 0HS UK; 19 People-Centred Artificial Intelligence Institute, University of Surrey, Guildford, UK; 20 Institute of Biochemistry and Genetics, Ufa Federal Research Centre Russian Academy of Sciences, Ufa, Russian Federation; 21 Section of Genetics and Genomics, Department of Metabolism, Digestion and Reproduction, Imperial College London, London, UK; 22 Translational Laboratory in Genetic Medicine (TLGM), Agency for Science, Technology and Research (A*STAR), 8A Biomedical Grove, Immunos, Level 5, Singapore, 138648 Singapore; 23 Lee Kong Chian School of Medicine, Nanyang Technological University, Singapore, Singapore; 24 Centre for Obesity Research, Rayne Institute, Department of Medicine, University College, London, WC1E 6JJ UK; 25 National Institute of Health Research University College London Hospitals Biomedical Research Centre, London, W1T 7DN UK

**Keywords:** Obesity, Type 2 diabetes

## Abstract

DNA methylation variations are prevalent in human obesity but evidence of a causative role in disease pathogenesis is limited. Here, we combine epigenome-wide association and integrative genomics to investigate the impact of adipocyte DNA methylation variations in human obesity. We discover extensive DNA methylation changes that are robustly associated with obesity (N = 190 samples, 691 loci in subcutaneous and 173 loci in visceral adipocytes, P < 1 × 10-7). We connect obesity-associated methylation variations to transcriptomic changes at >500 target genes, and identify putative methylation-transcription factor interactions. Through Mendelian Randomisation, we infer causal effects of methylation on obesity and obesity-induced metabolic disturbances at 59 independent loci. Targeted methylation sequencing, CRISPR-activation and gene silencing in adipocytes, further identifies regional methylation variations, underlying regulatory elements and novel cellular metabolic effects. Our results indicate DNA methylation is an important determinant of human obesity and its metabolic complications, and reveal mechanisms through which altered methylation may impact adipocyte functions.

## Abstract (web summary)

DNA methylation variation is associated with human obesity but a whether it plays a causal role in disease pathogenesis is unclear. Here, the authors perfom an integrative genomic study in human adipocytes to show that DNA methylation variations contribute to obesity and type 2 diabetes susceptibility, revealing underlying genomic and molecular mechanisms.

## Introduction

Obesity is a disease of excess adipose tissue that impairs health^1^. Worldwide there are more than 650 million people affected by obesity^2^. These individuals are at high risk of developing obesity-induced inflammatory and metabolic disturbances, and subsequent type 2 diabetes (T2D)^3–5^. Existing treatments for obesity and T2D have major limitations^6^, and new therapeutic targets derived from increased understanding of disease mechanisms are a global health priority.

DNA methylation, the first layer of epigenetic regulation, is causally implicated in human obesity and T2D through diverse aetiological pathways^7–9^. These pathways include in utero programming of disease risk, long-term effects of diet and lifestyle, mediation of causal genetic variations, age-related susceptibility and even inter/trans-generational inheritance^10–16^. However, previous efforts to identify robust causal associations between DNA methylation and these common harmful human conditions have been hindered by issues with tissue selection, cell specificity and assigning causation^17–21^. Obesity-associated DNA methylation changes in human blood are primarily a consequence rather than a cause of disease^22,23^. Methylation variations in human metabolic tissues cannot be confidently linked to phenotype due to cellular and therefore epigenetic heterogeneity^18,19^. Very few studies have investigated genome-wide DNA methylation in clinically relevant human cell types due to the substantial challenges in collecting and then isolating cells from human tissues^24–26^.

Adipocytes are the major cell type in adipose tissues. These specialist metabolic cells have important roles in local energy storage and expenditure, whole-body energy and glucose homoeostasis, and obesity and T2D pathogenesis^27,28^. Interestingly, adipocytes in distinct anatomical locations have variable DNA methylation and transcriptomic profiles, molecular functions and impacts on metabolic health, with visceral adipocytes considered more harmful than subcutaneous adipocytes^25,29,30^. Recent experimental studies demonstrate that manipulation of DNA methylation enzymes in adipocytes can induce or prevent obesity and T2D, through cellular effects on energy expenditure and insulin sensitivity^31,32^. These proof-of-principle studies provide strong rationale for exploratory epigenomic studies in human adipocytes.

Here, we addressed key limitations of previous human epigenome-wide association studies (EWAS) in subcutaneous and visceral adipocytes with the aim of identifying novel epigenetic mechanisms underlying obesity or its adverse metabolic consequences. We used an integrated genomic strategy to: (i) identify robust alterations in human adipocyte DNA methylation associated with extreme obesity; (ii) predict the potential effector transcripts (cis-target genes) of these DNA methylation changes; and (iii) infer mechanisms underlying these DNA methylation-gene expression relationships. At a subset of methylation sites and target genes, we used genetic association, targeted methylation sequencing, and adipocyte genetic and epigenomic manipulation to provide complementary evidence of causation. Our results highlight the importance of studying cell type-specific epigenomic variations and the power of extreme trait sampling. We provide mechanistic insights into the role of DNA methylation in human obesity and T2D, and deliver novel targets for detailed functional characterisation and potential clinical translation.

## Results

### Genome-wide alterations in adipocyte DNA methylation in people with extreme obesity

To identify obesity-associated alterations in human adipocyte DNA methylation (5-methylcytosine, 5mC) we collected subcutaneous and visceral adipose tissue samples intraoperatively from people with extreme obesity and healthy controls, and isolated populations of adipocytes from these tissues. We then characterised genome-wide DNA methylation in 190 subcutaneous and visceral adipocyte samples from obese cases and controls in separate discovery and replication cohorts (Illumina HumanMethylation450 and EPIC Beadchips, Supplementary Fig. 1). The mean difference in body mass index (BMI) between obese cases and controls from both discovery and replication cohorts was large (~20 kg/m2) (Supplementary Data. 1; age (±3.5-yrs), sex and ethnicity matched).

**Fig. 1:**
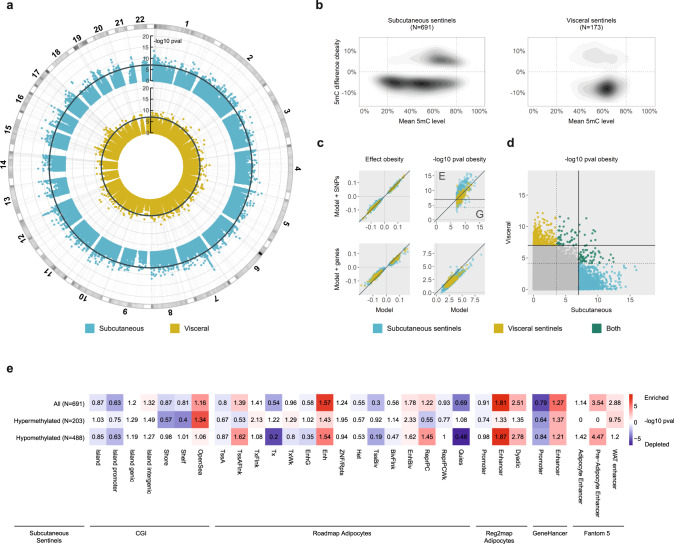
DNA methylation sites associated with extreme human obesity in subcutaneous and visceral adipocytes. **a** Genome-wide associations between 5mC and extreme obesity in subcutaneous and visceral adipocytes (*N* = 401,595 sites); -log10 pvalue in combined discovery and replication samples ordered by autosomal chromosome; threshold line epigenome-wide significance (EWS, *P* < 1 × 10^–7^). **b** 5mC differences between obese cases and controls relative to mean 5mC levels at *N* = 691 subcutaneous and *N* = 173 visceral sentinel methylation sites (%-methylation). **c** Comparisons of association models without and with adjustment for potential confounding variables to evaluate the effects of genetic variations and potential contaminating cell genes on 5mC-obesity relationships. Top panels: adjustment for cis-SNPs associated with each sentinel 5mC site (FDR < 0.01); effect size (beta) and -log10 pvalue in the combined discovery and replication cohorts; solid threshold EWS; dashed threshold Bonferroni corrected pvalue (0.05/N sentinels); G genetic effects; E non-genetic effects. Bottom panels: adjustment for principal components (PC1-5) derived from expression of 12 potential contaminating cell genes; effect size (beta) and -log10 pvalue in the replication cohort. **d** Cross tissue effects. Methylation-obesity association pvalue in subcutaneous and visceral adipocytes (combined discovery and replication samples); solid threshold EWS; dashed threshold Bonferroni corrected pvalue (0.05/N sentinels). All genome-wide association analyses were carried out separately in the discovery and replication cohorts using linear regression, and combined by inverse variance weighted meta-analysis. **e** Genomic annotation of obesity-associated DNA methylation sites. Numbered by fold change (observed compared to mean expected) and coloured by enrichment (red) or depletion (blue) -log10 pvalue (Fishers Exact Test, two-sided).

In subcutaneous adipocytes, we discovered 4485 5mC sites associated with extreme obesity at a false discovery rate (FDR) of <1%^33,34^. We then replicated the association of 5mC with obesity at 905 of these sites in an independent subcutaneous adipocyte sample (at FDR < 1% in the replication sample, and at epigenome-wide significance *P* < 1 × 10^–7^ in the combined discovery and replication samples, Fig. 1a). For subsequent genomic and functional analyses, we annotated the sentinel 5mC site at each replicated genomic locus (691 subcutaneous sentinels, lowest combined discovery and replication *P* value, ≥5-kb apart which is equivalent to reported CG island (CGI) widths, Supplementary Data. 2). Subcutaneous adipocyte sentinels had a median of 5.8% (range 1.1–17.9%) difference in methylation between obese cases and controls, and were systematically hypomethylated in obese cases (binomial sign test *P* < 6.4 × 10^–33^, Fig. 1b, consistent with previous reports^35^). More sentinels had intermediate (20–80%) than low (<20%) or high (>80%) methylation levels (Fig. 1b). 24 loci had ≥5 differentially methylated 5mC sites immediately flanking and within ±5-kb of the sentinel 5mC site (consistent association direction, *P* < 0.05, Bonferroni adjusted), indicating extended regions of differential methylation (Supplementary Data. 2). The largest differentially methylated regions were found at the *RUNX3* (19 sites, 1174-bp), *TBX5* (17 sites, 3187-bp) and *ISLR2* (14 sites, 4050-bp) loci. Overall, 4363 of the 4485 5mC sites (97%) identified at FDR < 1% in the discovery sample showed directional consistency for association with extreme obesity in the replication sample (binomial sign test *P* < 1 × 10-300), suggesting our findings represent only the strongest signals among a larger number of obesity-associated DNA methylation changes in subcutaneous adipocytes.

In visceral adipocytes, we identified 445 5mC sites associated with obesity at FDR < 1%, markedly fewer sites than in subcutaneous adipocytes. 220 of these 5mC sites replicated in an independent visceral adipocyte sample (replication FDR < 1%, combined discovery and replication *P* < 1 × 10^–7^, Fig. 1a). The 173 sentinel sites (visceral sentinels, lowest P value, ≥5-kb apart) had median methylation difference of 7.9% (range 2.9%-21.5%) between obese cases and controls (Fig. 1b, Supplementary Data 3). Visceral adipocyte sentinels were also preferentially hypomethylated in obesity (*P* = 3.8 × 10^–7^), and most sentinels had intermediate (20–80%) methylation levels (Fig. 1b). 2 loci showed extended regions of differential methylation (≥5 significantly differentially methylated 5mC sites within ±5-kb of the sentinel site, Supplementary Data 3); at *NFIA* (5 sites, 2081-bp) and near *ATG5* (5 sites, 831-bp). Again, overall directions of effect in visceral adipocytes were highly concordant between the discovery and replication samples (440 of 445 5mC sites (99%), *P* < 2 × 10^–123^).

Disease-associated alterations in DNA methylation are frequently the result of underlying differences in genetic polymorphisms or cellular (and therefore epigenetic) heterogeneity between individuals^18,19^. In sensitivity analyses, we found that the majority of sentinels remained associated with obesity after correction for genetic effects (Fig. 1c, Supplementary Fig. 2). Consistent with this, <1% of sentinels showed methylation distributions fitting with underlying SNP effects^36^, indicating that the identified methylation differences are predominantly environmentally driven. Similarly, we found that potential cellular heterogeneity (arising from impurity) and other unmeasured confounding exposures did not systematically alter our findings (Fig. 1c, Supplementary Fig. 3 and 4).

As adipose tissues from different depots have varying impacts on metabolic health^29^, we examined whether 5mC changes associated with obesity were specific to subcutaneous or visceral adipocytes. Only 23 subcutaneous adipocyte sentinels were robustly associated with obesity in visceral adipocytes, while a further 11 visceral sentinels replicated in subcutaneous adipocytes (consistent direction and *P* < 0.05, Bonferroni corrected for the number of sentinels, Fig. 1d, Supplementary Data 4). Similarly, we found only weak concordance when we compared overall directions of effect between subcutaneous and visceral adipocyte sentinels (374 of 671, *P* = 0.003 subcutaneous in visceral, and 105 of 173 *P* = 0.006 visceral in subcutaneous). We verified this depot specificity by demonstrating greater enrichment of subcutaneous (80% consistent direction and FDR < 0.01, *P* < 1.0 × 10^–300^) than visceral sentinels (21% consistent direction and FDR < 0.01, *P* = 9.3×10^–18^) in association with BMI among 538 whole subcutaneous adipose tissue samples^37^ (Supplementary Data 5).

Together, these genome-wide discovery and replication analyses quantify extensive alterations in DNA methylation in adipocytes from people with extreme obesity. Surprisingly, the majority of extreme obesity-associated 5mC changes are adipose depot specific, raising the possibility of tissue intrinsic biological origins and functions.

### Enrichment of extreme obesity-associated 5mC sites in active genomic regions

The genomic location of DNA methylation influences its gene regulatory potential and its mechanism of action^9,38,39^. To identify 5mC sites with active gene regulatory potential and evaluate underlying mechanisms, we mapped obesity-associated subcutaneous and visceral adipocyte sentinels to the human reference genome, human CpG island (CGI) annotations, and human adipose/adipocyte functional genomic annotations.

Differentially methylated subcutaneous adipocyte sentinels were significantly enriched in adipose tissue and adipocyte enhancers (Roadmap chromatin states, Reg2Map, FANTOM5^40–42^) and enhancers predicted from multifaceted datasets (GeneHancer^43^, Fig. 1e, Supplementary Fig. 5). Enrichment in enhancers was explained by both hypo- (lower methylation in obese adipocytes) and hyper- (higher methylation in obese adipocytes) methylated subcutaneous sentinels. Hypo-methylated sentinels were also (weakly) enriched in regions flanking active transcription start sites (TSS), bivalent enhancers and polycomb repressed regions, whereas hyper-methylated sentinels were generally underrepresented in these regions. In contrast, hyper-methylated sentinels were enriched and hypo-methylated sentinels were diminished in actively transcribed genic regions. In addition, subcutaneous sentinels were under-represented at promoter CGIs but not at intra- or inter-genic CGIs (Fig. 1e, Supplementary Fig. 5). Differentially methylated visceral adipocyte sentinels showed similar trends in CGIs and multifaceted enhancer datasets but were not significantly enriched in these, or in adipose tissue/adipocyte chromatin states (which notably are derived from the subcutaneous adipose depot, Supplementary Fig. 5). Our findings are consistent with previous studies localising variably methylated regions to cis-regulatory regions involved in transcriptional control and phenotypic variation^44–47^, rather than CG dense DNA sequences in the promoters of core housekeeping genes.

We also examined whether the genomic regions flanking our 5mC sentinels contained DNA sequence variants associated with human obesity phenotypes in genome-wide association studies (GWAS). Although we found no evidence of enrichment, 11 loci contained genetic variants associated with obesity (BMI^48^), 8 with central adiposity (waist-hip-ratio adjusted for BMI, WHR^49^) and 4 with T2D^50^, at genome-wide significance (*P* < 5 × 10^–8^, Supplementary Data 6); these loci included the BMI and adiposity locus *NRXN3*^51,52^, the WHR locus *TBX15*^53,54^ and the T2D locus *TCF7L2*^55^. This may reflect limited power to detect small genetic effects on phenotype mediated by DNA methylation. However, recent studies suggest modest overlap between EWAS and GWAS signals^56^, supporting the conclusion that most of the identified obesity-associated methylation changes in human adipocytes are environmentally rather than genetically determined.

### Predicted target genes of extreme obesity-associated 5mC sites

To identify genes that might be responsible for the effects of DNA methylation on phenotype (target effector genes), we carried out RNA sequencing in obese and control subcutaneous and visceral adipocytes with paired DNA methylation results (replication cohort, *N* = 89 samples). We split our 5mC sentinels into those in gene promoters, 5/3ʹUTRs and exons (*N* = 389 subcutaneous and *N* = 92 visceral sentinels) with unambiguous target genes, and those in intronic and distal intergenic regions (*N* = 302 subcutaneous and *N* = 81 visceral sentinels) without defined target genes.

**Fig. 2:**
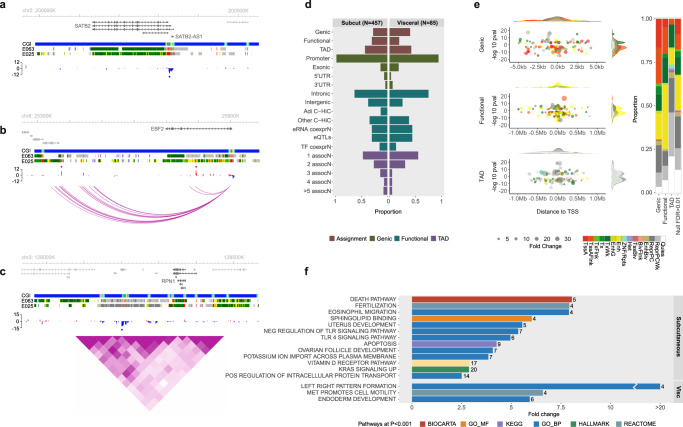
DNA methylation-target gene associations in human adipocytes. **a–c** Locus plots of sentinel 5mC sites (diamond) and their predicted target effector genes (dark grey). **a** Methylation at cg01558212 in the *SATB2* promoter was associated with *SATB2* and *SATB2-AS1* gene transcription (subcutaneous). **b** Methylation at two sites, cg11307296 and cg13390388, within distinct functional loops in human adipocyte promoter capture HiC connectivity maps, was associated with transcription of the adipocyte browning/beigeing gene *EBF2* (subcutaneous). **c** Methylation at cg03779326 was associated with transcription of *RPN1* but not other putative target genes within a shared human adipocyte TAD (visceral). Presented as %-difference in methylation between obese cases and controls, annotated by UCSC CpG island (CGI) and Roadmap adipose (E063) and adipocyte (E025) chromatin states. **d** Frequency of sentinel methylation-expression associations at FDR < 0.01 according to target gene assignment method. Genic: sentinel in promoter, 5/3ʹUTR or exon. Functional: intronic/intergenic sentinel sharing functional interaction with distal target gene. TAD: intronic/intergenic sentinel and distal target gene(s) within shared human adipocyte topologically associated domain. Adi C-HiC: human adipocyte promoter capture Hi-C interaction. Other C-HiC: promoter capture Hi-C interaction in another human tissue. eRNA coexprN: co-expression of distal eRNA and proximal promoter RNA. eQTLs: Association of distal SNP with proximal promoter expression. TF coexprN: TF binding in distal site (ChIP-seq) and TF-target gene co-expression. 1 to >5 assocN: Number of sentinel-target gene associations in shared TAD. **e** Subcutaneous sentinel-target gene associations at FDR < 0.01 grouped by target gene annotation method, coloured by adipocyte chromatin state (Roadmap E025). Left panel: distance to TSS and -log10 pvalue according to direction of effect, and sentinel density distribution. Right panel: frequencies of observed associations compared to the null background (sentinel-gene associations at FDR > 0.01). Fold change: log2 fold change in gene expression for each unit change in methylation. **f** Enriched pathways and genesets at *P* < 0.001 (Empirical, one-sided) based on the nearest cis−gene to each 5mC sentinel in subcutaneous and visceral adipocytes. Bar represents fold change of observed compared to mean expected frequency, number is the observed gene counts, in each pathway/geneset. All methylation-expression analyses were carried out using mixed-effects linear regression in combined adipocyte samples.

At promoters, 5/3ʹUTRs and exons, we examined the relationship between change in methylation at the 5mC sentinel site and change in expression of the overlapping/directly flanking cis-genes (mixed-effects model linear regression, combined subcutaneous and visceral adipocyte samples). Methylation at 121 subcutaneous adipocyte sentinels was robustly associated with expression of 126 unique cis-genes at FDR < 0.01 (P range 9.1×10-3 to 1.9×10-24, Fig. 2a, d, Supplementary Data 7). In addition, methylation at 29 visceral adipocyte sentinels was associated with expression of 32 unique cis-genes at FDR < 0.01 (*P* range 4.6 × 10^–3^ to 1.6 × 10^–12^, Fig. 2d, Supplementary Data 8). As expected, methylation changes at promoters, 5/3ʹUTRs and exons had predominantly negative effects on gene transcription (Binomial test *P* = 0.004, Fig. 2e). Subcutaneous sentinels associated with gene transcriptional changes were enriched in the genomic regions flanking active TSS rather than at active TSS (2.4-fold, Fisher’s exact *P* = 0.0005 in adipocytes and 2.4-fold, *P* = 7.4 × 10^–5^ in adipose, Fig. 2e, Supplementary Fig. 6).

At intergenic and intronic sites, we used human adipocyte chromosomal interaction maps (promoter capture Hi-C^57^) and enhancer-promoter inference datasets (GeneHancer^43^) to functionally assign 5mC sentinels to specific distal target genes, then tested sentinel 5mC sites for association with target gene expression. 84 subcutaneous and 14 visceral sentinels were associated with 135 and 16 unique distal target genes, respectively, at FDR < 0.01 (*P* range 1.1 × 10^–2^ to 8.6 × 10^–18^, Fig. 2b, d, Supplementary Data 9 and 10). For intergenic and intronic sentinels not assigned to target genes through direct functional interactions (*N* = 161 in subcutaneous and *N* = 56 in visceral) we localised the 5mC site to a human adipocyte topologically associated gene regulatory domain (TAD^58^) and defined all genes within the domain as potential targets (median 7 range 1-46 target genes per sentinel). 91 subcutaneous sentinels were associated with 177 cis-genes and 21 visceral sentinels with 34 cis-genes in the same TAD at FDR < 0.01 (*P* range 2.7 × 10^–3^ to 3.5 × 10^–20^, Fig. 2c, d, Supplementary Data 11 and 12). Most associations occurred in unique TADs, although 8 TADs had ≥5 sentinel methylation-target gene associations (Fig. 2d). As expected, sentinels associated with functionally assigned target genes were enriched in enhancers (subcutaneous 1.8-fold *P* = 8.4 × 10^–4^ in adipocytes and 1.5-fold *P* = 0.03 in adipose; visceral 3.1-fold P = 0.05 in adipocytes and 3.3-fold *P* = 0.01 in adipose, Fig. 2e and Supplementary Fig. 6). By contrast, sentinels associated with genes in a shared TAD were enriched in polycomb repressed regions (subcutaneous 1.5-fold *P* = 0.007 in adipocytes, 1.8-fold *P* = 0.0002 in adipose, Fig. 2e), suggesting that genomic context-specific regulatory mechanisms underlie the observed methylation-expression associations.

As the observed methylation differences between obese and control adipocytes were modest (median 5.8% at subcutaneous and 7.9% at visceral sentinels), we examined whether the identified target genes were systematically differentially expressed in adipocytes from their respective depot in association with obesity. In subcutaneous adipocytes, 34% of the target genes were differentially expressed at FDR < 0.01 (background 6%, binomial test *P* = 7 × 10^–75^, Supplementary Data 13). In visceral adipocytes, 32% of the target genes were differentially expressed (background 9%, binomial test *P* = 6 × 10^–37^, Supplementary Data 13). We then verified these findings in an independent human cohort comprising whole subcutaneous and visceral adipose tissues (GTEx Consortium). Despite the confounding effects of cellular heterogeneity in whole tissues^59^, the identified target genes showed consistent depot specific enrichment in association with BMI (Supplementary Data 13). We also examined whether altered expression of DNA methylating or demethylating enzymes might underlie the systematic hypomethylation observed in obese subcutaneous and visceral adipocytes, but found no evidence to support this explanation.

Many of the genes associated with altered DNA methylation in adipocytes have important roles in insulin signalling/sensitivity, adipogenesis, fatty acid metabolism and adipocytokine signalling (Supplementary Data 7 to 12). As examples, the *IRS2*, *ADIPOR2*, *PLIN5*, *FABP3, SOCS3, RBP4* and *AKT3* genes in subcutaneous adipocytes^60–66^, and the *KLF6*, *TULP3, DOC2B*, *ACOT11*, *PRKD2*, *SLC22A3* and *KIF5C* genes in visceral adipocytes^67–73^. Interestingly, several methylation-expression associations involved genes that control neurite formation, axonal guidance and synaptic plasticity (*NRN1*, *SEMA6B*, *SEMA4B* and *NRXN3*^74–76^), raising the possibility of epigenetic effects on neural inputs to expanding adipose tissue. Subcutaneous sentinels were associated with important genes involved in browning/beigeing of white adipocytes including the master regulator transcription factors *PRDM16* and *EBF2*^77,78^, and the signalling molecules *FGF9* and *IL10RA*^79,80^. The directions of effect of methylation on expression of *EBF2*, *FGF9* and *IL10RA* were all consistent with reduced browning in obese subcutaneous adipocytes. Whereas, at the *PRDM16* locus, methylation at different genomic positions was positively and negatively associated with *PRDM16* expression, suggesting complex methylation-expression relationships. We also discovered a novel association between expression of a cluster of micro-RNAs implicated in adipogenesis, *MIR23A*, *MIR24-2* and *MIR27A*^81–86^, and methylation of its shared promoter in subcutaneous adipocytes. Nonetheless, most potential effector genes of obesity-associated 5mC changes have unknown functions in adipocytes.

### Functional annotation of target genes linked to extreme obesity-associated 5mC changes

We used gene set enrichment analyses to systematically evaluate the molecular and functional significance of cis-genes linked to our 5mC sentinels either by genomic location (nearest cis-gene) or by association (methylation-expression FDR < 0.01). The nearest cis-genes to subcutaneous sentinel 5mC sites were enriched in growth and development, inflammatory, metabolic and apoptosis pathways (FDR < 0.01 Empirical, Fig. 2f, Supplementary Data 14). Notable pathways included *TLR4* signalling, a key trigger of the obesity-induced inflammatory response^87^, sphingolipid binding which is implicated in cell stress and metabolic dysfunction^88^, and Vitamin D metabolism which is linked to adipogenesis, lipid storage and adipocytokine production^89^. The genes nearest to visceral sentinels were over-represented in endoderm development, body pattern formation and cell motility pathways (FDR < 0.01 Empirical, Fig. 2f, Supplementary Data 14). Cis-genes associated with change in methylation at subcutaneous sentinels were enriched in a cluster of intersecting gene sets involved in transcriptional control and cell/tissue development (FDR < 0.01 Hypergeometric, gProfiler^90^). Relevant sub-clusters included cell differentiation and muscle development gene sets (Supplementary Fig. 7 and Supplementary Data 15). Similarly, cis-genes associated with visceral sentinels were enriched in intersecting embryonic and tissue development gene sets, and in transmembrane drug transporter genes (FDR < 0.01 Hypergeometric, gProfiler, Supplementary Fig. 7 and Supplementary Data 15). Taken together, these pathway analyses link obesity-associated 5mC sites in subcutaneous and visceral adipocytes to genes involved in cell lineage and fate determination, tissue development and remodelling, inflammation, and metabolic function/dysfunction, canonical functions of genomic enhancers^91,92^.

### Evidence of mechanistic interactions between transcription factors and extreme obesity-associated 5mC sites

Recent studies suggest that DNA methylation in enhancers and other active cis-regulatory regions may systematically regulate gene transcription by altering the binding of methylation-sensitive transcription factors (TFs^93–95^). Alternatively, TF binding can alter the methylation status of flanking DNA in these regions^94,95^. To investigate putative mechanistic interactions between obesity-associated 5mC changes and TFs, we mapped human TF binding motifs within ±150-bp of each sentinel 5mC site using the Homer database (de novo)^96^. We then identified the TFs with the strongest potential to bind to each motif, and examined the relationship between expression of these TFs, change in sentinel methylation and change in sentinel target gene transcription in adipocytes.

**Fig. 3:**
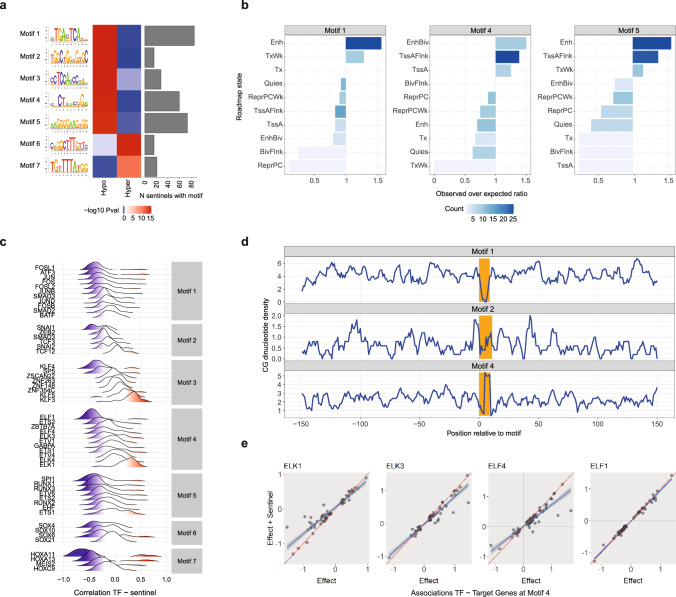
Interactions between DNA methylation and transcription factors in human subcutaneous adipocytes. **a** Enrichment of extreme obesity-associated DNA methylation sentinels in 7 transcription factor binding motifs (subcutaneous sentinels). Left panel: the predicted DNA sequence corresponding to each enriched motif, based on observed nucleotide frequencies (Homer). Motifs 2 and 4 both contained CG sites within their predicted DNA binding sequence. Centre panel: heatmap of -log10 pvalue for enrichment of: i. hypo- (lower in obesity); and ii. hyper-methylated subcutaneous sentinels (relative to permuted background, hypergeometric test, one-sided). Right panel: bar plot of the number of subcutaneous sentinels mapping to each motif. **b** Human adipocyte Roadmap chromatin state annotation of Motifs 1, 4 and 5; bar plots of observed over expected ratio in selected roadmap states, coloured by observed counts (number of sentinel-motif pairs). Motif 1 was over-represented in enhancers, Motif 4 in active TSS, and Motif 5 in enhancers and active TSS. **c** Density/ridge plots of pairwise correlation between TF expression and methylation level at each of its corresponding sentinels in subcutaneous adipocytes, split by Motif and ranked by mean correlation. **d** Distribution of genomic CG sites in the ±150-bp regions flanking Motifs 1, 2 and 4, centred on the motif (coloured in orange). Genomic CG sites were enriched at Motif 4 (peak), and depleted at Motif 1 (trough), relative to the flanking DNA sequences. A peak of genomic CGs was also observed immediately upstream of Motif 2 though other genomic CG peaks were present in its flanking DNA sequences. **e** Relationships between expression of 4 TFs predicted to bind at Motif 4 (*ELK1*, *ELK3*, *ELF1*, *ELF4*) and expression of the assigned target genes of each methylation sentinel corresponding to Motif 4. Presented as association beta without (Effect) and with (Effect + Sentinel) adjustment for sentinel DNA methylation level (combined adipocyte samples), with regression line and 95% confidence intervals. Adjustment for sentinel DNA methylation levels systematically influenced the associations between the *ELK1*, *ELK3* and *ELF4* TFs and their target genes, but not the *ELF1* TF.

The genomic regions flanking the 671 subcutaneous sentinels were enriched for 7 distinct TF binding motifs (*P* range 1 × 10^–9^ to 1 × 10^–13^); 5 motifs at hypo- (lower in obesity) and 2 motifs at hyper-methylated (higher in obesity) sentinels (median 28 range 16-86 sentinels per motif, Fig. 3a, Supplementary Data 16). Motif 1 was preferentially located at tissue-specific enhancers, Motif 4 at regions flanking active TSS, and Motif 5 at both enhancers and active TSS flanking regions (Fig. 3b, Supplementary Fig. 8). Overall, 49 TFs with potential to bind at these 7 motifs were expressed in human subcutaneous adipocytes (median 8 range 4–11 TFs per motif, Supplementary Data 17). In visceral adipocytes, we identified several putative motifs at obesity-associated 5mC sites (Supplementary Data 18) but none reached the stringent significance threshold required for robust de novo motif discovery.

Consistent with evidence that TF binding activity can alter DNA methylation levels, we found correlation between TF expression and sentinel methylation levels at each motif (Fig. 3c). The strongest correlations involved the *HOXA11* and *HOXA13* TFs (Motif 7, Fig. 3c), with weaker correlations between the *ATF3, FOSL1* and *JUN* (Motif 1), *SNAI1* (Motif 2), *KLF3*, *KLF4* and *KLF5* (Motif 3), *ELF1* and *ELK1* (Motif 4), *RUNX1*, *RUNX3* and SPI*1* (Motif 5), and *SOX4*, *SOX6* and *SOX10* (Motif 6) TFs and subsets of their sentinels (Fig. 3c). Motifs 2 and 4 contained CG sites within their binding sites, raising the possibility that the presence of DNA methylation might directly alter TF binding affinity at loci linked to these motifs (Fig. 3a, Supplementary Data 16). To evaluate this further, we examined whether these motifs were enriched for differentially methylated sites (sentinel and flanking sites) associated with obesity. No enrichment was observed but array coverage of potential CG sites was sparse (median 22% IQR 14-33% of genomic CG sites within ±150 bp of a Motif, Supplementary Fig. 8). As DNA methylation levels at adjacent CG sites are correlated^97^, we examined motifs for enrichment of genomic CG sites not covered in our dataset at which 5mC could impact TF-DNA binding. Motif 4 was strongly enriched for genomic CG sites relative to the flanking DNA sequences (±150-bp, Fig. 3d). A cluster of genomic CGs was also present immediately upstream of Motif 2, though other genomic CG clusters were observed in the DNA sequences flanking Motif 2. We therefore examined whether sentinel methylation levels at Motifs 2 and 4 influenced co-expression between TFs and their predicted target genes (the predicted target genes of each sentinel site corresponding to that TF-Motif pair). At both motifs, levels of methylation impacted TF-target gene relationships, suggesting potential mediation of transcriptional regulation by methylation (Fig. 3e, Supplementary Fig. 8). The largest effects of methylation on TF-target gene relationships were observed at the *ELK1*, *ELK3* and *ELF4* TFs (Fig. 3e), of which *ELK1* and *ELF4* have been shown to be methylation-sensitive in vitro^98,99^. Importantly, the TFs implicated in these reciprocal relationships with DNA methylation are widely involved in adipocyte biology, adiposity and metabolic dysfunction (Supplementary Data 17^54,67,100–109^).

### Genetic association analyses to infer disease causation

**Fig. 4:**
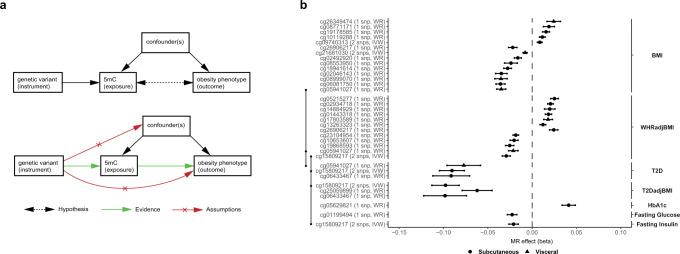
Causal inference analyses. **a** Mendelian Randomisation analysis using genetic variants as instrumental variables to evaluate cause-consequence relationships between DNA methylation sentinels and human obesity phenotypes. Required evidence: robust association between: i. the instrumental genetic variant and the methylation exposure; and ii. the instrumental genetic variant and the outcome phenotype. Assumptions: i. the instrument only influences the outcome through the exposure not through any other pathway (horizontal pleiotropy); and ii. the instrument is not associated with confounders. **b** Forest plots of the effect sizes of subcutaneous and visceral adipocyte sentinels causally associated with human obesity phenotypes through two sample MR in adipocytes (FDR < 0.01 in both MR causal and Steiger directionality tests). Centre values mark effect size estimates (MR beta) and error bars show the 95% confidence intervals. Sentinels causally associated with both adiposity and its metabolic consequences are annotated (connected lines). MR causal tests: Wald Ratio for single SNP IV (WR); Inverse Variance Weighted for >1 SNP IV (IVW). BMI: body mass index as a measure of obesity (GIANT, *N* ≤ 795,640). WHRadjBMI: Waist-hip ratio adjusted for BMI as a measure of central adiposity (GIANT, *N* ≤ 694,649). T2D and T2D adjusted for BMI as measures of T2D risk (DIAGRAM, *N* ≤ 231,422). Fasting glucose and insulin (MAGIC, *N* ≤ 138,589) and HbA1c (MAGIC, *N* ≤ 159,940) as measures of glycaemic traits linked to T2D.

To distinguish individual 5mC sites with potential causal effects on obesity or obesity-induced metabolic disturbances, we carried out two sample Mendelian Randomisation analyses (MR). We used whole adipose tissue (*N* = 588 samples, Twins UK^110–113^) rather than isolated adipocytes to increase power to identify independent cis-SNPs (pairwise linkage disequilibrium (LD) R2 < 0.01) associated with each sentinel 5mC site (within +/500-kb) and selected a significance threshold of 0.05 (Bonferroni corrected) to reduce weak instrument bias. We then used the identified cis-SNPs as instrumental variables (IV) to infer causal effects of DNA methylation on obesity, central adiposity, T2D, glycaemic traits linked to T2D, and lipid traits, among large-scale human GWAS (Fig. 4a^48–50,114,115^).

Only 191 subcutaneous and 34 visceral sentinels had significantly associated cis-SNPs, meaning we were unable to assess causation at a large fraction of sites. Nevertheless, we found genetic evidence to support causal effects of 5mC on obesity (measured using BMI) at 10 loci in subcutaneous adipocytes and 4 loci in visceral adipocytes (MR single instrument Wald Ratio or multiple instrument IVW random effects FDR < 0.01, and Steiger directionality test FDR < 0.01, Fig. 4b, Supplementary Data 19^116,117^). We also identified potential causal effects of DNA methylation on central adiposity at 12 loci (10 subcutaneous, 2 visceral, measured using WHR), T2D at 4 loci (3 subcutaneous, 1 visceral), and fasting glucose, insulin and HbA1c at 3 loci (all subcutaneous, Fig. 4b, Supplementary Data 19). Methylation at 3 loci was causally linked to multiple obesity phenotypes: cg26906217 (BMI and WHR; near the *PIK3C2A* gene); cg15809217 (WHR, T2D and fasting insulin (FI); associated with *PRRC2A* expression in subcutaneous adipocytes); and cg05941027 (BMI, WHR and T2D; associated with *LIMD2* expression in visceral adipocytes). Another notable locus was the 5mC site cg21681030, causally associated with BMI through MR, and respectively associated with expression of *RHOQ*, a glucose receptor trafficking gene^118,119^, in our human adipocyte samples. For blood lipids traits, we identified potential causal associations between DNA methylation and HDL cholesterol at 15 loci (14 subcutaneous, 1 visceral), LDL cholesterol at 11 loci (10 subcutaneous, 1 visceral), triglycerides at 3 loci (2 subcutaneous, 1 visceral), and multiple lipid types at 8 loci (all subcutaneous, Supplementary Fig. 9, Supplementary Data 19). 6 of the 37 lipid loci were also causally implicated through MR in adiposity or its metabolic consequences (Supplementary Fig. 9).

As our MR analyses were predominantly based on single SNPs as IVs, we repeated them using a larger number of correlated cis-SNPs (pairwise r2 < 0.8, SNP-methylation *P* < 0.05, Bonferroni corrected) as IVs to evaluate for horizontal pleiotropy (Fig. 4a). 44 loci had at least 3 correlated IV SNPs enabling sensitivity testing at these loci. 30 loci replicated using MR IVW regression and 10 replicated using the less powerful MR Egger regression (*P* < 0.05, Supplementary Data 19). Multiple loci showed evidence of heterogeneity between IVs, but only 11 loci violated the MR assumption of no horizontal pleiotropy (MR Egger intersect *P* < 0.05, Supplementary Data 19). We then evaluated the relevancy of our MR findings to adipocytes by replication testing the SNP-5mC associations identified in whole adipose tissue in subcutaneous and visceral adipocytes. Despite the small sample size, 21 of 61 cis-SNPs replicated in adipocytes at *P* < 0.05 (Binomial Test *P* = 8.2×10^–13^) and 50 of 61 cis-SNPs had consistent directions of effect (Binomial Test *P* = 4.6 × 10^–7^, Supplementary Data 20). Thus, through genetic association, we infer causal effects of adipocyte DNA methylation on obesity or obesity-induced metabolic disturbances at up to 28 independent genomic loci, and on lipid traits at up to 31 further genomic loci.

### Adipocyte functional studies and methylation fine-mapping

Finally, we selected two target genes of 5mC sites implicated in disease pathogenesis through MR, but without known metabolic functions, for functional screening in adipocytes. We focused on the *PRRC2A* gene which was linked to central adiposity, insulin resistance and T2D, and the *LIMD2* gene which was linked to obesity, central adiposity and T2D.

**Fig. 5:**
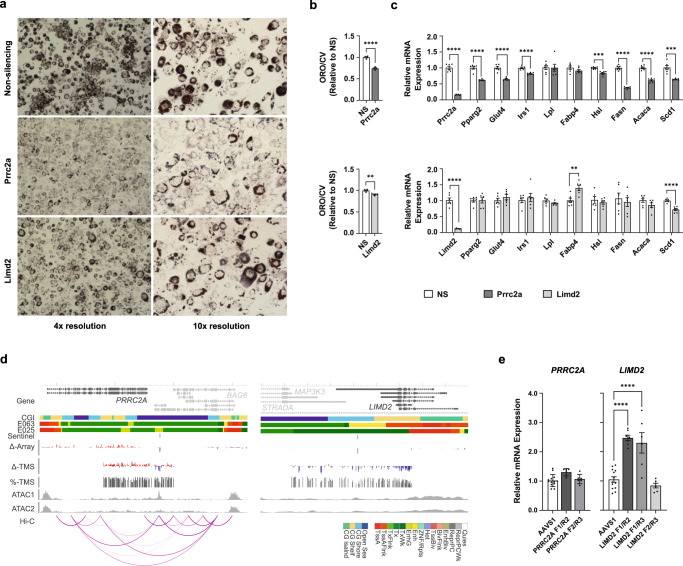
Adipocyte genomic and functional studies. **a** Oil Red O (ORO, red/brown) lipid staining in day 6 differentiated 3T3-L1 adipocytes reverse transfected with non-silencing (NS), *Prrc2*a or *Limd2* siRNA at day 2 of differentiation. **b** Equivalent spectrophotometric measurements of eluted ORO, normalised for cell number using crystal violet (CV, day 6, *N* = 4 independent samples, *Prrc2a* *P* = 3.5 × 10^–5^, *Limd2* *P* = 0.0047). Presented as mean ± SEM relative to NS control, compared by Student’s *t* test (two-sided). **c** Expression of adipogenesis, insulin signalling and lipid metabolism genes at day 6 of differentiation in 3T3-L1 adipocytes transfected with siRNA against *Prrc2a*, *Limd2* or NS control at day 2 of differentiation (*N* = 6 independent samples). Real-time qPCR values were normalised to housekeeping genes (*Nono*, *Ywhaz*). Presented as mean ± SEM relative to NS control, compared by Student’s *t* test (two-sided). **d** Targeted methylation sequencing at the *PRRC2A* (left panel, subcutaneous adipocytes, *N* = 43) and *LIMD2* (right panel, visceral adipocytes, *N* = 46) loci. CGI: UCSC CpG islands. E063 and E025: Roadmap adipose and adipocyte chromatin states. Sentinel: Sentinel methylation site in combined discovery and replication data. Δ-Array and Δ-TMS: Difference in methylation (range −10 to 10%) in obesity in combined discovery and replication array data, and targeted methylation sequencing data (red higher, blue lower). %-TMS: Mean methylation level (0 to 100%) in targeted methylation sequencing data. ATAC1 and ATAC2: ATAC sequencing of human undifferentiated preadipocytes and mature differentiated adipocytes. Hi-C: Human adipocyte Hi-C functional connectivity maps at day 3 of differentiation. **e** Targeted activation at the *PRRC2A* locus in human adipocytes (using two pairs of guides, F1/R2 *N* = 6 and F2/R3 *N* = 6 independent samples) had no effect on *PRRC2A* expression. Targeted activation at the *LIMD2* locus increased *LIMD2* expression in two (F1/R2 *N* = 7 and F1/R3 *N* = 6 independent samples) but not a third cell line (F2/R3 *N* = 6 independent samples). Presented as mean ± SEM relative to *AAVS1* control cells, standardised to housekeeping genes (*ACTB*, *GAPDH*). *AAVS1* represents the combined results for the *AAVS1* F1/R2 and F1/R3 guide pairs (*N* = 13 independent samples, One-Way ANOVA test, two-sided, Dunnett’s test for multiple comparisons). **P < 0.01, ***P < 0.001, ****P < 0.0001. Source data are provided in the Source Data file.

We evaluated the effect of silencing each target gene on a cellular model of adipogenesis, because adipogenesis has an important role in adipose tissue expansion and insulin sensitivity. We found that knockdown of both genes significantly reduced lipid accumulation during adipocyte differentiation, with a more marked effect observed after *Prrc2a* than *Limd2* knockdown (Fig. 5a, b, Supplementary Fig. 10). As adipocyte differentiation models are prone to variability, we independently replicated our lipid accumulation studies, observing concordant results (Supplementary Fig. 10). We also examined the effects of *Prrc2a* and *Limd2* gene silencing on key adipogenesis, lipid metabolism and insulin signalling genes. *Prrc2a* knockdown led to a consistent reduction in expression of *Pparg*, the master regulator adipogenic transcription factor, and concordant reductions in lipid metabolism and insulin signalling genes that are regulated by *Pparg* (Fig. 5c, Supplementary Fig. 10). In contrast, *Limd2* gene silencing did not alter *Pparg* expression (Fig. 5c, Supplementary Fig. 10). Instead, *Limd2* silencing led to alterations in fat mobilising and lipid synthesis genes, though findings were inconsistent across replicates (Fig. 5c, Supplementary Fig. 10). We repeated our gene silencing studies using two distinct siRNAs for each gene, targeting different sites on the mRNA, which verified that our findings were not due to off target effects or altered cell viability (Supplementary Fig. 11).

In parallel, we used targeted methylation sequencing to fine map obesity-associated DNA methylation changes in the regulatory regions flanking the sentinel sites at *PRRC2A*, *LIMD2* and 69 additional loci (*N* = 43 subcutaneous and *N* = 46 visceral adipocyte samples, Supplementary Data 21). Although targeted methylation sequencing lacked the precision of methylation arrays or whole-genome bisulphite sequencing^120^ (Supplementary Fig. 12), we found that methylation sentinels were enriched for association with obesity in targeted sequencing results, and effect sizes were highly concordant with array results (Supplementary Fig. 12). We therefore selected a > 5% change at *P* < 0.05 as evidence of differential methylation at sites tagged by robust sentinel associations. At the *PRRC2A* locus, we identified multiple differentially methylated sites adjacent to the sentinel at an open chromatin region^121^, annotated as an intronic enhancer, with functional connectivity to the *PRRC2A* gene^58^ (Fig. 5D, Supplementary Fig. 13, Supplementary Data 22). Here, expression of 2 TFs predicted to bind at the methylation sites (*ELK1* and *ESSR*) correlated strongly with *PRRC2A* expression and explained >30% of its variance (Supplementary Fig. 14, Supplementary Data 23), linking the methylation changes to TF-mediated control of *PRRC2A* transcription. The underlying open chromatin region and enhancer elements were specific to adipocyte precursors (they were not present in mature adipocytes or whole adipose tissue) supporting our gene silencing and published findings implicating *PRRC2A* in adipogenesis and cell specification^122^. At the *LIMD2* locus, we revealed an extended region of differential methylation covering a proximal enhancer and several *LIMD2* exons and splice sites (Fig. 5D, Supplementary Fig. 13, Supplementary Data 22) at which methylation might impact transcription initiation, elongation or splicing. At other loci with established roles in adipocyte biology and/or genetic risk, targeted methylation expanded the sentinel associations to differentially methylated regions, the functional units by which DNA methylation controls gene expression and phenotype, suggesting potential pathogenic effects at these loci (Supplementary Fig. 15, Supplementary Data 22). At loci linked to Motif 4, we found that the CG dinucleotide positions within the motif were systematically hypomethylated in people with obesity (18 of 24, 75%, *P* = 0.01, Supplementary Fig. 14) and that the methylation differences at these sites were concordant with those observed at the adjacent sentinel sites (R = 0.63, *P* = 0.001, Supplementary Fig. 14). Thus establishing that the binding motifs of methylation-sensitive transcription factors are enriched for methylation changes associated with obesity that influence TF-target gene co-expression relationships (Fig. 3e).

As the sites of methylation changes were distal to the *PRRC2A* and *LIMD2* promoters, we used CRISPR-activation to assess whether the regulatory elements harbouring obesity-associated methylation changes were involved in target gene transcription. At both loci, we targeted differentially methylated open chromatin peaks at/flanking the sentinel sites using a dual guide-based approach to maximise potential activation^123^ (Supplementary Fig. 16). Targeted activation significantly increased expression of *LIMD2* (>2-fold, *P* < 0.0001) but not its neighbouring genes (Fig. 5e, Supplementary Fig. 17). Targeted activation did not alter *PRRC2A* expression or expression of the *BAG6* gene which shares the same TAD (Fig. 5e, Supplementary Fig. 17). These experimental studies confirm unequivocally that the differentially methylated genomic region at *LIMD2* regulates *LIMD2* transcription. They do not however rule out a comparable effect at the *PRRC2A* locus where the absence of activation may be due to technical factors^124–126^ considering the combinatorial genomic evidence linking the differentially methylated region to *PRRC2A* expression.

Overall, our integrative genomic and adipocyte functional studies are consistent with lower methylation levels at cg15809217 in obese subcutaneous adipocytes promoting insulin resistance and T2D, through reduced *PRRC2A* expression and impaired adipogenesis. They also suggest that reduced methylation at cg05941027 in obese visceral adipocytes may increase *LIMD2* expression and thereby increase lipid storage. *PRRC2A* was recently reported as a reader of N6-methyladenosine (m6A), the most abundant internal modification to mRNA, that stabilises transcripts as they translocate from the nucleus to ribosomes for protein synthesis and regulates cell specification, offering a novel mechanism through which its metabolic effects may be mediated^122,127^. *LIMD2* is a structural protein requiring further elucidation^128^.

## Discussion

Epigenetic processes are tissue and cell specific which has made investigating their roles in complex human diseases a major challenge. In this study, we combine integrated functional genomics with extreme trait sampling in human adipocytes, from functionally distinct adipose depots, to elucidate epigenetic mechanisms underlying human obesity and obesity-induced metabolic disturbances. We discover and replicate extensive changes in DNA methylation associated with extreme obesity at epigenome-wide significance. Surprisingly, these methylation changes are largely adipose depot-specific with many more obesity-associated 5mC sites occurring in subcutaneous than visceral adipocytes. We surmise this depot specificity may be due to the local tissue microenvironment in the absence of technical, genetic or other known confounding factors.

By integrating our DNA methylation findings with adipocyte-specific transcriptomic and chromosomal interaction datasets, and cross-tissue enhancer-promoter catalogues, we statistically and functionally connect extreme obesity-associated 5mC sites to transcriptomic changes at >500 genes. These putative effector genes of obesity-associated methylation changes cluster in developmental, metabolic and inflammatory pathways, and encode proteins with key roles in adipogenesis, browning/beigeing of white adipocytes and insulin signalling. Of particular interest, we associate lower levels of DNA methylation in obese subcutaneous adipocytes with increased expression of a micro-RNA cluster, comprising *MIR23A*, *MIR24-2* and *MIR27A*, whose members have been shown to inhibit *PPARG* signalling, suppress adipogenesis and induce insulin resistance^81–86^.

Our analyses co-localise extreme obesity-associated 5mC variations to functionally active genomic regions and transcription factor binding sites. They further suggest that TF activity may alter methylation status and that methylation status may impact TF activity at differentially methylated sites associated with obesity. These findings require experimental validation but are supported by existing evidence of reciprocal relationships between DNA methylation and TFs, and methylation-sensitivity of the TFs we identify^93–95,98^. At a cellular level, many of the TF families linked to obesity-associated DNA methylation variations – AP1, KLF, SOX and ETS – have established or emergent roles in adipocyte biology and metabolism^67,100–103,108^, connecting disease-related DNA methylation variations to potential pathogenic pathways.

Contrary to most previous human obesity EWAS, we causally implicate up to 26% of extreme obesity-associated 5mC sites in obesity and obesity-induced metabolic disease susceptibility. Our MR findings should be regarded as causal estimates of the effects of methylation on phenotype under genetic control^129^. They do not indicate absence of causation at environmentally determined loci, the majority of our dataset. Targeted methylation sequencing and CRISPR-activation add to our understanding of regional methylation patterns and their putative impact on gene expression regulation. Complementary functional screens of target genes of causative methylation variations demonstrate novel effects on adipogenesis, *PPARG* signalling and adipocyte lipid handling, offering cellular mechanisms by which DNA methylation may promote obesity and its consequences.

New technologies for profiling DNA methylation and transcriptional regulation at single cell resolution will enable future studies to address the importance of cellular epigenetic heterogeneity, methylation and transcriptional dynamics, and spatial microenvironmental interactions, in obese adipose tissue remodelling^130,131^. Nevertheless, we show that existing technologies that facilitate the study of epigenomic variations in larger numbers of individuals, and thus better capture human phenotypic diversity, remain a valuable tool for de-convoluting the epigenetic basis of human obesity and T2D. Combining these high-throughput approaches with precision epigenome tools^132–135^ will clarify the impact of DNA methylation, including at the sites we identify, on disease pathogenesis.

Taken together, our exploratory studies in human adipocytes begin to reveal genomic mechanisms and molecular signalling pathways through which DNA methylation may impact human obesity and its metabolic consequences. We provide new evidence of causation at a sizeable fraction of extreme obesity-associated 5mC sites, and a resource of epigenomic variations and genes for furthering our understanding of the human epigenome and its role in obesity and its metabolic complications.

## Methods

### Study design

Case-control DNA methylation analyses were carried out in 95 subcutaneous and 95 visceral adipocyte samples from people with extreme obesity and healthy controls in separate discovery and replication cohorts (Supplementary Fig. 1). In total, 44 participants in the discovery cohort and 42 participants in the replication cohort provided both subcutaneous and visceral adipocyte samples. The remaining subcutaneous and visceral adipocyte samples came from distinct individuals. RNA sequencing was carried out in adipocytes from the replication cohort.

### Participant selection and sample processing

Adipose tissue samples were obtained intraoperatively from morbidly obese individuals (mean (sd) BMI 44.8 (7.2) kg/m2) undergoing laparoscopic bariatric surgery and healthy controls (mean (sd) BMI 24.9 (3.3) kg/m2) undergoing non-bariatric laparoscopic abdominal surgery (Supplementary Data 1). Subcutaneous tissue was collected from abdominal surgical incision sites and visceral tissue from the omentum. Participants were unrelated, between 20-70 years of age, from a multi-ethnic background, and free from systemic illnesses not related to obesity. Controls and cases were well-matched for age (within 3.5-yrs), sex and ethnicity (Supplementary Data 1). People with treated T2D were excluded because of potential effects of hypoglycaemic medications on adipose tissue metabolism. All participants gave informed consent. The study was approved by the London—City Road and Hampstead Research Ethics Committee, United Kingdom (reference. ^13^/LO/0477).

Whole tissue samples were processed immediately to isolate populations of primary adipocytes using established protocols^136,137^. Polypropylene plastics were used to minimise adipocyte cell lysis. Tissues were cut into 1-2-mm3 pieces and washed in Hank’s buffered salt solution (HBSS), before digestion using type 1 collagenase (1 mg/ml, Worthington) in a water bath at 37 C, shaking at 100-rpm for ~30-min. Digested samples were filtered through a 300-micron nylon mesh to remove debris, and the filtered solution was centrifuged at low speed (500-g; 5-min; 4 C). After removal of the oil layer, floating mature adipocytes were collected by pipette, washed in ~5x volume of HBSS and recentrifuged. After 3 washes the clean adipocyte cell suspension was collected for snap freezing and storage at −80 C.

### Quantification of DNA methylation

Genomic DNA and RNA were extracted in parallel from isolated adipocytes using the Qiagen AllPrep DNA/RNA/miRNA Universal Kit according to the manufacturer’s protocol for lipid-rich samples. Genome-wide DNA methylation was assayed using Illumina Infinium HumanMethylation450 (96 discovery samples) and EPIC (96 replication samples) beadchips. In both cohorts, all samples were randomised and processed in single batches. 0.2–0.5 µg of genomic DNA was bisulphite converted using EZ DNA Methylation-Direct Kits (Zymo Research, Irvine, CA). Bisulfite-treated DNA was denatured, neutralised and subjected to an overnight whole-genome amplification reaction. Amplified DNA was enzymatically fragmented, precipitated and resuspended for hybridisation to respective HumanMethylation450K and EPIC beadchips. After hybridization, beadchips were processed through a primer-extension protocol, stained, coated and then imaged using the HiScan System (Illumina).

Raw signal intensities were retrieved using the readIDAT function of the R package Minfi (version 1.36.0, Bioconductor^138^), followed by background correction with the function bgcorrect.illumina from the same R package. Detection P values were derived using the function detectionP as the probability of the total signal (methylation + unmethylated) being detected above the background signal level, as estimated from negative control probes. Signals with detection P values ≥ 0.01 were removed. >99% of CG sites in both cohorts passed this quality control threshold. One visceral (discovery) and one subcutaneous (replication) sample with less than 95% of CG sites providing a detected signal were excluded. To reduce non-biological variability between observations, data were quantile normalised with the function normalizeQuantiles of the R package limma (version 2.12.0, Bioconductor). DNA methylation was quantified on a scale of 0–1, where 1 represents 100% methylation.

Separate principal component analyses (PCA) were carried out on HumanMethylation450K and EPIC positive control probe signal intensities. These probes assess multiple steps in the laboratory processing and the resulting principal components (PCs), which capture technical variability in the experiment, were included as covariates in our discovery and replication models to remove technical biases^139^.

### RNA sequencing

RNA sequencing was carried out in the 96 samples with paired DNA methylation results from the replication cohort. Sample order was randomised for library preparation and sequencing. Total RNA was quantified using the Qubit Fluorometer (RNA HS Assay) and aliquots of 10-ng were used for library preparation. RNA integrity numbers (RINs, Aligent 2100 Bioanalyzer) ranged from 2.5 to 7.9 (median 5.8), an indication of suboptimal transcript quality in isolated human adipocytes (stored at −80 C). Sequencing libraries were therefore generated using the SMARTer Stranded Total RNA-Seq Kits (v2 Pico input) which uses Switching Mechanism at the 5ʹ end of RNA Template technology and random priming to make cDNA from low-quality, low-input RNA volumes. Takara Bio/Clontech recommendations were followed at each library preparation stage (fragmentation, first-strand cDNA synthesis, addition of adapters and indexing, AMPure bead purification, library amplification, AMPure bead repeat purification). Final libraries were validated using the Aligent BioAnalyzer (DNA1000 and High Sensitivity chips) and 2 failed libraries were removed. Sample-specific libraries were pooled at equimolar concentrations (4 nM) to avoid batch effects and sequenced according to the manufacturer’s protocols using the HiSeq 4000 (16 lanes). Each sample was sequenced to >80 million 100-bp paired-end reads.

Raw sequencing data was demultiplexed using Bcl2Fastq (version 2.20) and read quality was checked using fastQC (version 0.11.5). Reads were adapter trimmed (Illumina generic adapters) and mapped against the Ensembl human reference genome GRCh37 v87 using the splice aware aligner STAR (version 2.6.0, options: -outFilterMultimapNmax 20 -outFilterMismatchNoverReadLmax 0.04). Lane level bams were merged, sorted and indexed using Samtools (version 1.9) to create library level bams for each sample. Mapping rates and quality control measures were evaluated level using Picard (version 2.18.12) and RseQC (version 2.6.4), and summarised using MultiQC (version 1.9). Raw counts per gene were generated using FeatureCounts (subread version 1.6.2); multimapped reads were included (options: -M -fraction) to capture short reads resulting from lower quality transcripts. 2 samples with low number of aligned reads (<15 M) reflecting low-quality libraries were excluded. 3 other outlying samples with low median transcript integrity (TIN) or evidence of GC bias were removed (Supplementary Fig. 18). After exclusions, gene expression results were available for 43 subcutaneous and 46 visceral adipocyte samples and 24,187 genes (counts ≥5 in ≥20% of samples). As a final QC step, surrogate variable analysis (SVA^140^) was carried out on variance stabilising transformed counts (generated using DESeq2, version 1.28.0^141^) for each sample to capture unwanted variation in our RNA sequencing data, particularly that arising due to differences in transcript and sequencing quality (Supplementary Fig. 18). The resulting surrogate variables (SV) were included as covariates in 5mC regression models to remove associations driven by sample and sequencing biases.

### Epigenome-wide association analyses

All methylation-phenotype analyses were carried out separately in subcutaneous and visceral adipocytes. 44,101 probes were removed because of known cross hybridisation^142,143^ or presence of a common genetic variant (SNP, indels, or structural variation, 1000 Genomes European phase 3 dataset, MAF > 1%) within the probe sequence. After QC and removal of problem probes, only the 401,595 single CG sites present on both the Illumina HumanMethylation450 and EPIC beadchips were investigated. Single markers passing quality control were tested for association with extreme obesity using linear regression and an established analytic strategy to reduce batch and other technical confounding effects^139^. Obesity status was used as the predictor with methylation as the outcome variable to generate %-methylation differences between cases and controls. Covariate adjustments were made for potential biological (age, sex and ethnicity) and technical confounding variables (the first 4 control probe PCs which explained >95% of control probe technical variation). Association results from the discovery and replication cohorts were combined using inverse variance weighted (IVW) meta-analysis and evaluated for heterogeneity. Sex-stratified analyses were not carried due to limited power to detect sex-specific effects in our cohorts.

Statistical significance was inferred at FDR < 0.01^33,34^ in the discovery sample to provide an inclusive set of biologically relevant results. A more stringent cut off of (i) FDR < 0.01 in the replication sample (with consistent direction of effect) and (ii) combined *P* < 1 × 10^–7^ (epigenome-wide significance, EWS^139^) in the combined discovery and replication samples was then used to define significant associations. Markers associated with obesity at *P* < 1 × 10^–7^ within ±5000-bp of each other were considered as a single genomic locus. A ± 5000-bp distance was selected to take into account the known sizes of discrete regions of DNA methylation, for example CGIs^144^. At each independent locus the CG site with lowest P value for association with obesity was defined as the sentinel marker.

Depot-specificity was evaluated by replication testing sentinel sites identified in subcutaneous and/or visceral adipocytes among *N* = 538 independent whole subcutaneous adipose tissue samples from the Twins UK cohort (cohort description provided in Methods: Genetic association analyses section). Methylation profiling, quality control and data analysis was carried out as described previously^37^. 822 of the 864 identified methylation sentinels were available for replication testing after quality control filtering. Individual sentinel sites were tested for association with linear change in BMI using mixed effects models adjusting for biological and technical covariates (fixed effects: age, smoking status, cell type proportion, methylation chip, sample position; random effects: zygosity, family). An exact binomial test (R function binom.test) was used to test whether consist directions of effect between discovery and replication, and between subcutaneous and visceral, were observed more often than expected by chance.

### Genetic confounding and adipocyte purity

Genotype data for each participant was generated from whole blood using Illumina Infinium OmniExpress-24 v1.2 beadchips. We removed directly genotyped SNPs with call rates <90%, minor allele frequency <0.01, Hardy-Weinberg Equilibrium *P* < 1 × 10^–6^, SNPs on sex chromosomes and duplicated SNPs. After quality control, 649,007 SNPs were taken forward for imputation. SHAPEIT^145^ was used to infer haplotypes, and imputation was carried out in IMPUTE2 (version 2.3.2^146^) using the 1000 genome reference panel Phase 3 (all ancestries). Each chromosome was divided into 5 Mb chunks for imputation and merged at the end. A random seed was supplied automatically. Effective population size (Ne) reflecting genetic diversity was 20,000 as recommended when using a multi-population reference panel. After imputation, genotype data was available for 81,656,368 SNPs. Sensitivity analyses were carried out in combined discovery and replication results (IVW meta-analysis). Multivariate regression models with and without genetic confounding factors were compared (Supplementary Fig. 2A and B). First, PCA was performed on participant GWAS data and the first 5 PCs explaining >95% of inter-individual variation were included as covariates to adjust for population stratification. Second, the effects of cis-SNPs (within 500-kb) on methylation-phenotype associations were examined by including (i) the cis-SNP most strongly associated with DNA methylation and (ii) all independent cis-SNPs associated with DNA methylation at FDR < 0.01 (pairwise LD R2 < 0.8) in multivariate regression models. Gaphunting from the Minfi package was also used to identify and flag methylation sites at which the distribution of methylation was consistent with an underlying SNP driven effect (version 1.36.0^36,138^ Supplementary Data 2 and 3).

Purity of the isolated adipocytes was evaluated using two approaches. First, in the replication cohort, RNA sequencing results for immune and stromovascular cell-specific genes were used to evaluate associations driven by potential contamination (broad immune cell—*PTPRC*; monocyte/macrophage—*CD68*, *ITGAM*, *CD14*; B/T cell—*TNFRSF8*, *CD19*, *CD4*, *CD8A*, *CD2*; NK cell—*NCAM1*; endothelial cell—*CD31*; preadipocyte—*DLK1*, *CD34*). Replication models without and with variance stabilised transformed gene expression counts (DESeq2, version 1.28.0) were compared for each immune- or stromovascular cell-specific gene (Supplementary Fig. 3A and B). PCA analysis was also carried out to summarise the variance across the potential contaminating cell genes, and models without and with PCs were compared (Supplementary Fig. 3A and B). Second, in the combined discovery and replication cohorts, SVA analysis was performed to capture unexplained variation due to cellular heterogeneity (resulting from contamination) and other potential confounding factors in our high-dimensional DNA methylation data^140,147–149^. The resulting SV were then included as covariates in regression models to test whether the observed associations were driven by impurity or other unknown confounding factors (Supplementary Fig. 4).

### Methylation-expression analyses

Individual sentinel 5mC sites were assigned to potential target genes using three approaches. First, sentinels in exons, 5/3ʹ UTRs or within 5-kb of a promoter were assigned to overlapping genes. A 5-kb cut-off to define a promoter region was selected based on an observed drop off in the number of 5mC sentinels beyond this distance from their respective TSS (using sequential 1-kb bins). Second, for 5mC sites in intronic/intergenic regions, we overlapped the sentinel methylation site with distal chromosomal intervals connected to proximal gene transcription start sites from: i. Adipocyte Capture Hi-C results; and ii. the GeneHancer enhancer-promoter inference database^43^. Human adipocyte Capture Hi-C data was available at GEO (Accession ID: GSE110619^57^) as pre-processed and pre-called interactions. GeneHancer interactions were taken from the combined standard and elite functional interaction sets, with removal of standard interactions defined by proximity alone. Third, for those intronic/intergenic sentinels not assigned to a functional targeted gene, we intersected the sentinel sites with adipocyte-specific TADs, and took all genes within intersected sentinel-TAD pairs as potential targets. Adipocyte TADs were called from available Hi-C data generated at day 3 of human adipocyte differentiation in vitro (GEO, Accession ID: GSE109924^58^). TADs were called from bed files, using Arrowhead at 25-kb resolution with default parameters, and merged, removing hierarchal structures by retaining largest domains to maximise TAD coverage, for a final set of 5323 domains of median 425-kb, range 125-kb to 4,025-kb intervals.

Sentinel 5mC sites were tested for association with expression of each of their functionally assigned target genes using linear regression. Initially, we carried out association testing separately in subcutaneous and visceral adipocytes, but had limited power to detect methylation-expression associations. Thus, for our final analyses we used the combined subcutaneous and visceral adipocyte datasets to identify methylation-expression relationships, and mixed-effect modelling to control for sample relatedness (i.e. subcutaneous and visceral adipocytes from the same individuals). Mixed effect models were implemented using Dream analysis from the Bioconductor package variancePartition (version 1.18.0) in R; methylation betas were used as the predictor and logCPM transformed gene expression counts (voomWithDreamWeights) as the outcome. Study participant ID was included as a random effect. Two methylation control probe PCs, two SVs generated using SVA of the gene expression counts, age, sex, and ethnicity, and RNA integrity numbers (RINs) were included as fixed effects to adjust for technical, biological and sample-related biases. Mixed model results from Dream were compared with those from the nlme R package (version 3.1-149, using variance stabilised transformed counts from DESeq2, version 1.28.0) as a further sensitivity test. Statistical significance was inferred at FDR < 0.01 (qvalue version 2.20.0^33^) based on the number of methylation-target gene pairs analysed.

Differential expression analyses in obese cases and controls were carried out separately in subcutaneous and visceral adipocytes to evaluate concordance with methylation-target gene expression changes (DESeq2, raw counts, adjusted for age, sex, ethnicity, RIN and 2 SVs). Differential expression analyses (DESeq2) were also carried out across the range of BMI in bulk RNA sequencing data from whole subcutaneous (*N* = 663) and visceral (*N* = 541) adipose tissue samples in the GTEx Consortium database (dbGaP accession number phs000424.v8.p2, 23/02/2022) using raw counts adjusted for age, sex, ethnicity and the SVs generated by SVA (separately in subcutaneous and visceral tissues). Enrichment was evaluated using an exact binomial test comparing the observed (number of differentially expressed target genes) with the background expected (total number of differentially expressed genes) success rates.

### Functional enrichment analyses

All functional enrichment analyses were carried out using permutation testing as Illumina methylation arrays preferentially evaluate pre-selected genomic sites (e.g. CG islands) and well-annotated genes. For each sentinel CG site, we identified a permutation set of 1000 unique CG sites with equivalent methylation levels and variability in their respective subcutaneous or visceral adipocyte samples, using the following criteria: i. difference in mean between sentinel and permutation; ii. difference in standard deviation (sd) between sentinel and permutation; and iii. >5-kb distance between sentinel and permutation (i.e. not at the same genomic locus). For each sentinel, difference in mean and sd were based on a sliding scale starting at mean 0.025 and sd 0.0025 and increasing incrementally by mean 0.025 and sd 0.0025 until >1000 independent permutated CG sites were achieved. This approach was selected because a low fixed mean/sd was too stringent to generate 1000 permutations at some sentinels, while a higher fixed mean/sd was too permissive at other sentinels.

For genomic enrichment analyses, we compared the number of sentinels located in a genomic feature (observed frequency) with that in each of the 1000 permutation sets (expected frequency). For human obesity and metabolic disease GWAS enrichment analyses, we identified methylation sites and GWAS SNPs (*P* < 5 × 10^–8^) in shared same Adipocyte Roadmap Chromatin State (E025), and compared the frequencies between the sentinels and the permuted background. We used the same permutation-based approach for our nearest gene pathway analyses, but limited our analyses to one sentinel per gene and one permutation per gene, to avoid recounting genes. The nearest gene to each sentinel was identified using the ChIPseeker annotation package (version 1.28.3^150^), which prioritises overlaps in promoters over other features (ranking: promoter, 5/3ʹUTR, Exon, Intron, Intergenic). Nearest genes were cross-referenced with the Molecular Signatures Database (MsigDB, hallmark, curated and ontology gene sets^151,152^). Differences in observed and expected frequencies were calculated using the Fishers Exact test and Empirical P values. Gene set enrichment analyses of genes associated with methylation in adipocytes were carried out in gProfiler^90^, using a background of the nearest gene to each of the 1000 permuted CG sites, limited to genes expressed in our human adipocyte RNA sequencing data.

### Transcription factor binding site analyses

De novo transcription factor binding motif enrichment analysis was performed using the script fingMotifGenome.pl from the Homer program package (version 4.11.1). Subcutaneous and visceral sentinels were investigated separately. Regions of interest were selected by extending each sentinel site for ±150-bp on each side. The enrichment analysis was done using two different backgrounds as controls: i. the ±150-bp regions around the 1000 permutation sites specific to each sentinel; and ii. genomic regions with GC% matching those of sentinel regions. At each motif, we identified the 10 transcription factors most likely to interact with that motif (best match between known motifs and the discovered motif) to provide a sizeable but manageable number of TFs. We then restricted these TFs to those expressed in our human RNA sequencing data (counts ≥5 in ≥20% of samples).

Positions in the region around the sentinel of respective motifs for analysis were inferred with the annotatePeaks.pl script from the Homer program package, with the options -rmrevopp to account for palindromic motifs. Genomic CpG sites relative to motifs were retrieved using the Bioconductor package seqPattern (version 1.20.0) in R. Correlation analyses of TF expression and methylation of their corresponding sentinels were carried out in the depot in which the sentinel was identified. Relationships between TFs, their respective sentinels and the predicted target genes of each sentinel (assigned sequentially first by promoter/exon/UTR overlap, then by functional interaction, then by shared TAD) were studied in combined subcutaneous and visceral adipocyte samples to increase power. Associations between TF and target gene expression were examined (i) without and (ii) with adjustment for sentinel DNA methylation level to explore the effects of sentinel methylation sites on TF-target gene relationships. Mixed effects models were carried out in the package nlme package (version 3.1_149) to adjust for sample relatedness; age, sex, ethnicity, two methylation control probe PCs and two gene expression SVs were included as fixed effect covariates to adjust for potential confounding variables. For all TF expression analyses, variance stabilising transformed counts (DESeq2, version 1.28.0) were used.

### Genetic association analyses

Two sample Mendelian Randomisation (MR) analyses were carried out to investigate causal relationships between individual sentinel 5mC sites and human obesity phenotypes using: i. 588 whole subcutaneous adipose tissue (WSAT) samples from the Twins UK cohort; and ii. summary results from recent large-scale human GWAS.

The Twins UK cohort is a nationwide registry of healthy volunteer twins in the United Kingdom, with about 14,000 registered twins since 1992, predominately Caucasian female (84%) and equal number of monozygotic and same-sex dizygotic twins. Twins UK phenotypic measurements, adipose tissue biopsies, genome-wide SNP and DNA methylation assays were performed as previously described^110–112^. Briefly, samples were genotyped using HumanHap300, HumanHap610Q, HumanHap1M Duo, and HumanHap1.2 M Duo 1 M arrays. Haplotypes from IMPUTE2 (without a reference panel) were used for fast imputation to the 1000 Genomes phase 1 dataset. Imputed SNPs were excluded based on Hardy Weinberg equilibrium (P < 1e-6), allele frequency cut-offs (MAF < 0.01), missingness (>5%) and imputation quality (info score < 0.8). Individuals with mis-assigned sex or ancestry outliers were removed. Ancestry outliers (> 7 SD) were obtained from PLINK 2.0 (unrelated) and GENESIS (related participants). Related individuals with IBS > 0.125 (PLINK 2.0) were also excluded. DNA methylation profiles in adipose tissue biopsies were obtained as described previously^112^. Methylation results were available for 588 out of 596 twins after further quality control analyses^113^. All individuals were female (mean (sd) age 59.1 (9.4)).

Human GWAS comprised: BMI as a measure of obesity (GIANT 2018, transethnic^48^); WHR adjusted for BMI as a measure of central adiposity (GIANT 2018, transethnic^49^); fasting glucose and insulin (MAGIC, Europeans, unpublished); HbA1c (MAGIC 2017^114^); T2D and T2D adjusted from BMI (DIAGRAM 2018, Europeans^50^); HDL and LDL cholesterol and triglycerides (Global Lipids Genetics Consortium 2021, transethnic^115^).

Cis-SNPs within ±500-kb of each subcutaneous and visceral sentinel were tested for association with change in sentinel DNA methylation level in WSAT using linear regression. DNA methylation levels were adjusted for technical covariates, age, predicted smoking, family relatedness, genetic principal components (PCs) and non-genetic DNA methylation PCs. Methylation-genotype associations were evaluated in the MatrixEQTL package in R (version 2.1.0) using linear models, with the adjusted methylation values as the dependent variable and the dosage of alternative allele the independent variable. Ambiguous palindromic cis-SNPs with MAF > 0.42 were removed. For each sentinel, cis-SNPs were clumped (linkage disequilibrium (LD) R2 < 0.01) and independent methylation quantitative trait locus (mQTL) SNPs associated with DNA methylation at *P* < 0.05 (Bonferroni corrected for the number of SNPs) were selected. Primary MR analyses of these mQTL SNPs and human GWAS phenotypes were implemented in R using the TwoSampleMR package (version 0.5.1^116,117^). Causal relationships were tested using the most powerful MR method (Wald Test for single mQTL SNPs, and Inverse Variance Weighted method for multiple mQTL SNPs). Cause-consequence directions of effect were evaluated using the Steiger directionality test, which compares SNP-methylation and SNP-phenotype R2 values. Potential causal effects of methylation on phenotype inferred if both the MR and Steiger tests passed a significance threshold of FDR < 0.01.

MR sensitivity testing was carried out using two approaches. First, we evaluated MR assumptions by repeating our MR analyses using correlated cis-SNPs (within ±500-kb, clumped at LD R2 > 0.8, and associated with methylation at *P* < 0.05 Bonferroni corrected) in the R package MendelianRandomization (version 0.4.1); correlated cis-SNPs were used as no sentinels had ≥3 uncorrelated cis-SNPs for such analyses. MR IVW and MR Egger regression were used to test for: i. replication; ii. heterogeneity between SNPs; and iii. evidence of horizontal pleiotropy; at each sentinel with ≥3 correlated cis-mQTLs. Second, we replication tested WSAT mQTLs implicated in positive MR results amongst our subcutaneous and visceral adipocyte samples using SNP as the predictor, methylation beta as the outcome, and adjusting for biological and technical confounders (age, sex, ethnicity, control probe PCs 1-4). For WSAT mQTL SNPs not present in our adipocyte dataset, we identified a proxy SNP present in adipocytes (the cis-SNP ( ± 500-kb) with the greatest pairwise LD with the mQTL SNP and minimum R2 > 0.8) and used the correlated allele to evaluate for association with methylation and concordance of directions of effect.

### In vitro gene silencing studies

The 3T3-L1 pre-adipocyte mouse cell line (ATCC-CL-173) was obtained commercially (LGC). Pre-adipocytes were grown in Dulbecco’s Modified Eagles Media with 10% newborn calf serum (NCS) and 1% penicillin/ streptomycin (P/S). Two days post-confluence (Day 0) differentiation was induced by switching cells to DMEM supplemented with 10% foetal bovine serum (FBS), 10-µg/ml insulin, 0.5-mM IBMX, 1-µM dexamethasone and 2-µM rosiglitazone. On day 2, cell media was refreshed with insulin media (DMEM containing 10-µg/ml insulin). Cells were maintained at 37 C and 5% CO_2_ and were differentiated in 10-cm dishes until undergoing siRNA reverse transfection.

Early differentiation 3T3-L1 adipocytes were reverse transfected at Day 2 of differentiation with Silencer Select siRNAs (Ambion) as described below, adapting reported methods^153^. Two different siRNAs against each target were used in concert to enhance target gene knockdown. Cells were transfected with siRNAs against either *Limd2* (ID; s85672; s85674), *Prrc2a* (ID; s79278; s79279) or a non-silencing (NS) (ID; Negative control #1) siRNA. RNAiMax lipofectamine transfection reagent (Life Technologies) and siRNAs were diluted separately in Opti-MEM media (Gibco), mixed together, added to empty wells and incubated for 20-mins before cell suspension was seeded. To each well of a 6-well and 12-well plate, 50-pmol of siRNA (25-pmol each siRNA) and 30-pmol siRNA (15-pmol each siRNA) were added respectively. For NS control, a single siRNA was used at the same total pmol quantity as for targets. Differentiating 3T3-L1 adipocytes in 10-cm dishes were detached at Day 2 of differentiation by treating with Tryple Express (Gibco) for 10-mins. Cells were counted, resuspended at 450,000 cells/ml in DMEM insulin media (insulin, 10-µg/ml), and added to 12-well (1 ml; 450,000 cells/well) and 6-well plates (2 ml; 900,000 cells/well) containing the pre-incubated siRNA transfection mix. The next day cells were refreshed with new insulin media (10-µg/ml insulin). On day 6, siRNA treated differentiated cells were harvested for RNA (6-well plates) or assayed for lipid accumulation (12-well plate; Oil Red O). The two distinct siRNAs for each target gene, which were designed to target different regions of the *Prrc2a* and *Limd2* mRNAs, were also tested individually to rule out off target effects.

Oil red O (ORO) staining was performed to assess lipid accumulation in mature 3T3-L1 cells (Day 6) that were reverse transfected with siRNA at early differentiation (Day 2). The protocol used was similar to that described previously^154^ with modifications. Cells were washed with PBS and fixed with 10% neutral formalin for 1-h. After formalin removal, cells were washed with sterile water then exposed to 60% isopropanol for 3-mins. After removal of 60% isopropanol, cells were stained with ORO solution (Sigma) for 10-mins, and then washed with water until all extracellular ORO was completely removed. At this point images of ORO staining at 4X and 10X magnification were acquired using the Evos m7000 microscope (Thermo Scientific). Cells were treated with 100% isopropanol for 10-mins to extract ORO stain from lipid in cells for quantification by measuring elute absorbance at 500-nM using SpectrumMax 340PC plate reader (Molecular Devices). Samples were added to 96-well plate in quadruplicate along with known ORO quantities (µg/ml) to make a standard curve to calculate µg of ORO eluted. Following ORO elution, cells were washed 2x with water to allow crystal violet (CV) nuclear staining for relative cell number normalisation. Cells were stained with 0.05% CV for 10-mins, followed by 4×10-min washes with water to remove all extracellular CV. SDS (1%) was added to cell plates and incubated for 10-mins with constant orbital agitation at 150 rpm to lyse cells and allow CV absorbance in the lysate to be measured at 560-nM using the SpectrumMax 340PC plate reader. CV samples were added to 96-well plates in quadruplicate along with known CV quantities (µg/ml) to generate a standard curve to calculate µg of CV and to thus normalise ORO data to relative cell number.

Total RNA was isolated from siRNA transfected 3T3-L1 adipocytes at Day 6 using Qiazol reagent and the RNeasy mini kit (Qiagen) according to manufacturers’ instructions, with on-column DNase (Qiagen) treatment performed during RNA isolation. The High-Capacity RNA-to-cDNA kit was used to generate cDNA by reverse transcription of 1-µg total RNA. RT-qPCR gene expression analysis was performed using the CFX384 Touch Real-Time PCR Detection System (BioRad), SSO advanced Universal SYBR Green Supermix mix, gene-specific primers (500-nM final concentration) and cDNA in a 10-µl total reaction volume. qPCR conditions were: 3-min at 95 C, then 40 cycles of 95 C for 10-s, 60 C for 30-s and followed by melting curve analysis from 65-95 C in 0.5 C steps at 5-secs/step. Sequences of primers used in qPCR analysis are listed in Supplementary Data 24. Gene expression was quantified using the delta-delta Ct (2-ΔΔCT) method and is shown relative to the non-silencing group. Two housekeeping genes *Nono* and *Ywhaz* were utilised, with their geometric mean expression being used for normalisation. Effects of knockdown on genes involved in adipocyte differentiation (*Pparg*), insulin signalling (*Glut4*, *Irs1*), lipid uptake (*Lpl*), lipid storage (*Fasn*, *Acaca*, *Scd1*) and lipid mobilisation (*Fabp4*, *Hsl*) were evaluated^155^.

Cell viability was evaluated in siRNA treated 3T3-L1 cells using the RealTime-Glo™ MT Cell Viability Assay (Promega). Cells were transfected with individual siRNAs against *Limd2* (IDs s85672 and s85674), *Prrc2a* (ID, s79278 and s79279) or a non-silencing (NS) (ID; Negative control #1) siRNA as described above. Briefly, 3T3-L1 cells were simultaneously plated into 96-well white-walled clear-bottom assay plates (5,000 cells/well), treated with siRNAs (2-pmol siRNA/well) and incubated with the RealTime-Glo™ reagent according to the manufacturer’s protocol. The luminescence signal (Relative Light Units), which corresponds to the number of metabolically active cells, was then measured at 4-h, 24-h, 48-h and 72-h post siRNA treatment in these live cells.

GraphPad Prism was used to perform Student’s *t* tests (two groups) or One-Way ANOVA tests (multiple groups, Dunnett’s test for multiple comparisons) for analyses of gene expression, Oil Red O and luminescence. Gene expression and Oil Red O data are shown relative to the non-silencing group. All data are presented as means ± SEM.

### In vitro CRISPR-activation studies

CRISPR-activation at the *PRRC2A* and *LIMD2* loci was carried out in 2 steps, transduction of the CRISPR-activation and then guide RNA (gRNA) vectors, in polyclonal human adipocytes. For each target gene we designed 3 distinct guides (Supplementary Fig. 16, Supplementary Data 25) and paired these guides in 3 different combinations to target the CRISPR-activation complex to the region of interest (Termed: F1/R2 for Guides 1 and 2; F1/R3 for Guides 1 and 3; F2/R3 for Guides 2 and 3). CRISPR gRNA were designed in CRISPOR^156^ and CHOPCHOP^157^ to target a 150-bp window at the peak of respective open chromatin sites (Supplementary Fig. 16, Supplementary Data 25). The top 3 guides with the lowest off-target scores were selected for use in pairs to target the CRISPR-activation systems to the 150-bp site (Supplementary Data 25). 3 control gRNA were selected from published studies^158,159^ to target adeno-associated virus integration site 1 (*AAVS1*), a non-functioning viral integration site encoded on human chromosome 19. Guide RNA plasmids were synthesised using a previously published dual gRNA approach^123^. Primers containing target gRNA and BsmBI ligation sites (Supplementary Data 26) were used on template pScaffold-H1 (Addgene #118152) DNA to generate PCR products containing dual gRNA that were then ligated into the LentiGuide-Hygro (Addgene# 139462) vector in a single-step digestion-ligation reaction. The resulting vectors were transformed into NEB stable bacteria and successful clones were confirmed by Sanger sequencing (Supplementary Data 25).

HEK293T cells grown in Dulbecco’s modified Eagle’s medium (DMEM) with 10% foetal bovine serum (FBS) and 1% penicillin/streptomycin (P/S) and incubated at 5% CO2 and 37 C, were co-transfected with LentiGuide-Hygro gRNA vectors using PEIpro (PolyPlus) and packaging plasmids (pMD2.G, psPAX2, Addgene #12259 and #12260). Cell media was changed 16-h after transfection. Lentiviral particles were collected at 24-h, 48-h and 72-h after media change, concentrated overnight using Lenti-X Concentrator (Takara Bio #631231), resuspended in media and stored at −80C. Lentiviral titre were quantified using qPCR Lentivirus Titre Kit (abm #LV900).

Immortalized Human Adipose-derived Stromal (ihASC) cells overexpressing *Bmi-1* and *hTERT*^160^ were obtained commercially (abm #T0540). ihASC cells were grown in Dulbecco’s Modified Eagle Medium/Nutrient Mixture F-12 (DMEM/F-12; Gibco) with 10% foetal bovine serum (FBS; Sigma), 2 ng/ml Fibroblast Growth Factor (FGF) and 50-µg/ml gentamicin (Gibco), and maintained at 37 C and 5% CO_2_. ihASC cells were transduced with lenti-dCAS9-VP64 (CRISPR-a, Addgene #61425-LVC) lentivirus and polybrene (10-µg/mL) in 6-well plates by reverse transduction. Virus was removed and media changed 48-h post transduction. Transduced ihASC cells were treated with blasticidin (5-µg/mL) until controls were dead (~ 60–84-h). Successfully transduced and selected polyclonal CRISPR-a (lenti-dCAS-VP64) ihASC cells were then transduced with LentiGuide-Hygro gRNA lentivirus using the same approach, except with hygromycin (50-µg/mL) treatment. 1 guide pair for *PRRC2A* and 1 guide pair for *AAVS1* did not survive selection leaving 2 guide pairs for *PRRC2A*, 3 for *LIMD2* and 2 for *AAVS1*.

After transduction and selection, genomic DNA and total RNA was isolated using the AllPrep Mini kit (Qiagen) according to manufacturers’ instructions, with on-column DNase treatment during RNA isolation. High capacity RNA-to-cDNA kits (Applied Biosystems #4387406) were used to generate cDNA from 1-µg RNA. RT-qPCR gene expression analysis was performed using the method described previously, using sequence-specific primers to amplify: the target genes (*PRRC2A*, *LIMD2*); neighbouring genes (*BAG6*, *STRADA*, *MAP3K3*); 2 house-keeping genes (*ACTB*, *GAPDH*, Supplementary Data 26). Successful vector transduction in transduced cells was confirmed by PCR amplification of DNA using primers specific to each vector (targeting *Cas9* for lenti-dCAS9-VP64, and *Hygro* for LentiGuide-Hygro) and gel electrophoresis of the products (Supplementary Fig. 17A, Supplementary Data 26). Correct guide pair insertion was verified by PCR amplification of DNA using primers targeting the LentiGuide-Hygro vector either side of the guide sequences (Supplementary Data 26), and Sanger Sequencing of the products (Supplementary Data 25). Transcriptional activation of the target gene, or neighbouring genes within the same TAD, was quantified using the delta-delta Ct (2-ΔΔCT) method relative to the *AAVS1* controls. Successful activation was assessed by comparing expression of the targeted gene in its CRISPR-activation cell lines with expression of the targeted gene: i. control *AAVS1* cell lines; and ii. the CRISPR activation cell lines targeting a different gene, in which we would expect no change in expression of that target gene. Specificity of activation was evaluated by measuring expression of other neighbouring genes in the same TAD as the target gene (Supplementary Fig. 17B and C). All results were standardised to the *ACTB* and *GAPDH* housekeeping genes. GraphPad Prism was used to perform One-Way ANOVA tests for comparisons of gene expression between multiple groups of activated and control cells.

Lenti-dCAS-VP64_Blast was a gift from Feng Zhang (Addgene #61425-LVC^125^); LentiGuide-Hygro was a gift from Caroline Goujon (Addgene #139462); pMD2.G and psPAX2 were a gift from Didier Trono (Addgene #12259, #12260); pScaffold-H1 was provided by the Cebola lab^161^.

### Targeted methylation sequencing

Targeted methylation sequencing was carried using the TWIST Bioscience Methylation Detection System, and a customised probe panel, in two stages: i. a technical pilot on 8 human adipocyte samples; ii. a larger study of 94 human adipocyte samples including repeat of the 8 pilot samples (23 obese and 24 control subcutaneous, 23 obese and 24 control visceral, from both the discovery replication cohorts). Sample processing and library preparation was carried out according to the TWIST Bioscience end-to-end targeted methylation sequencing workflow. Probes were designed, optimised and synthesised using TWIST’s proprietary algorithms and olio synthesis solutions to target 71 independent genomics regions (totalling 58.9-kb). For each sample, 20-ng of genomic DNA was used for fragmentation to ~265 bp (Covaris E220x), A-tailing, universal adapter ligation, enzymatic methylation conversion (New England Biolabs® EMseq^TM^) and PCR amplification (9-16 cycles). 47-200-ng of each enzymatic converted, amplified, indexed library was then hybridised in multiplex (pools of 8-samples) to the customised panel probes (120-mins), captured using streptavidin beads, amplified and then purified to generate target-enriched enzyme-converted DNA libraries for sequencing. Pilot libraries were sequenced with spike in negative and positive controls using the Illumina NovaSeq 6000 platform (100-bp paired-end); main study libraries were sequenced across 3 runs of Illumina NextSeq 2000 P2 (100-bp paired-end); both aiming for a median coverage of >100X.

Raw sequencing data was demultiplexed using BCLConvert (Version 4.0.3) and read quality was evaluated using FastQC. Fastq files from the 3 sequencing runs were merged (GNU coreutils) for processing and quality control analyses (using default settings unless specified). Reads were adapter trimmed using fastp (Version 0.22.0) with autodetection of adapter length, and then aligned to the reference genome (Ensembl GRCh37, bwameth Version 0.2.5, samtools Version 1.6, sambamba Version 0.8.2) following the TWIST recommended pipeline. Duplicates were identified and removed. Picard (CollectHsMetrics, CollectMultipleMetrics, Version 2.6.0) and MethylDackel (mbias, Version 0.5.1) were used to generate sequencing quality metrics, including on-target coverage and methylation conversion measurements. Methylation levels at individual sites were called using MethylDackel (–minDepth 10–maxVariantFrac 0.25–OT 0,0,0,96—OB 0,0,4,0; except for 9 samples where—OT and—OB values were customised to trim methylation biases observed at the ends of reads). The BISulfite-seq CUI Toolkit (BISCUIT^162^, version 1.0.2) was used to call SNPs/genotypes and confirm sample identities using a version of the Van Andel Institute Bioinformatics and Biostatistics Core Snakemake workflow^163,164^ adapted for the Altair PBS Professional workload manager. Methylation.bed files were processed using the Methrix package (Version 4.2) in R. 5 samples with low median on target coverage (<25X) were removed. For each remaining sample, methylation sites with low coverage (<25X), overlapping known SNPs (MAF > 0.01, 1000-Genomes Phase 3), and not in the genomic regions targeted by probes, were removed. Differences in DNA methylation between obese cases and controls were analysed separately for subcutaneous and visceral adipocytes, using linear models adjusting for biological covariates (age, sex and ethnicity). Concordance, precision and platform biases were evaluated in: i. the 8 samples present in the pilot and main cohorts; and ii. the main cohort at sites present in both the array and targeted methylation sequencing datasets. At the *PRRC2A* locus, the sequences (extended by ±6-bp around) around the 5 methylation sites in the region of interest were used as input for scanning with 692 human motifs in the JASPAR2022 database^165^. The sequences were scanned using the searchSeq function from the TFBSTools Bioconductor package, v1.36.0^166^ with a threshold of 85% motif similarity.

### Reporting summary

Further information on research design is available in the Nature Portfolio Reporting Summary linked to this article.

## Supplementary information

Supplementary information

Description of Additional Supplementary Files

Supplementary Data 1-26

Reporting Summary

## Data Availability

Genome-wide DNA methylation data from the discovery and replication cohorts are deposited in the Gene Expression Omnibus (GEO accession No: GSE222595). Raw RNA sequencing data from the replication cohort are available in the European Genome-Phenome Archive (EGA study no. EGAS00001007118) to provide managed open access to genetic variant containing data. Associated participant characteristics are provided for both methylation and RNA sequencing datasets. Methylation bedGraph files of the methylation differences in obese compared to lean subcutaneous and visceral adipocytes (combined discovery and replication cohort) are also available at GEO (accession No: GSE222595). The following publicly available datasets were used in this study: GeneHancer^43^ (https://www.genecards.org/), Human adipocyte Capture Hi-C^57^ (GSE110619), Human adipocyte TADs^58^ (GSE109924), Human adipocyte ATAC^121^ (GSE110734), Roadmap Epigenomes (https://egg2.wustl.edu/roadmap/web_portal/), JASPAR2022^165^ (https://jaspar.genereg.net/) and the Molecular Signatures Database (MsigDB^151^ https://www.gsea-msigdb.org/gsea/msigdb/). Human GWAS summary statistics were obtained from: GIANT^48^ (https://portals.broadinstitute.org/collaboration/giant/index.php/GIANT_consortium_data_files), MAGIC^114^ (https://magicinvestigators.org/downloads/), DIAGRAM^50^ (https://diagram-consortium.org/downloads.html), and the Global Lipids Genetics Consortium^115^ (https://csg.sph.umich.edu/willer/public/glgc-lipids2021/). Source data for adipocyte functional studies are provided with this paper. Source data are provided with this paper.

## Source data

Source data

## Code availability

Data analysis pipelines used in this study for epigenome-wide association, methylation-expression, methylation-transcription factor, TwoSample Mendelian Randomisation analyses, and targeted methylation sequencing, can be obtained from https://github.com/WRScottImperial/Human-adipocyte-5mC-obesity.
