# Supplementary material for: Integrative genomic analyses in adipocytes implicate DNA methylation in human obesity and diabetes
Source: Nat Commun. 2023 May 15;14:2784. doi: 10.1038/s41467-023-38439-z (PMC10185556; doi:10.1038/s41467-023-38439-z)
Supplement: Supplementary file 1 — Supplementary information [file 41467_2023_38439_MOESM1_ESM.pdf]

Supplementary Figure 1
Study Design.

Supplementary Figure 2A
Effect of genetic factors on methylation-phenotype associations in subcutaneous adipocytes.

Supplementary Figure 2B
Effect of genetic factors on methylation-phenotype associations in visceral adipocytes.

Supplementary Figure 3A
Evaluation of potential adipocyte impurity by contaminating gene expression in subcutaneous adipocytes.

Supplementary Figure 3B
Evaluation of potential adipocyte impurity by contaminating gene expression in visceral adipocytes.

Supplementary Figure 4
Evaluation of potential adipocyte impurity by SVA.

Supplementary Figure 5
Genomic annotation of subcutaneous and visceral sentinels.

Supplementary Figure 6
DNA methylation-target gene associations in human subcutaneous and visceral adipocytes annotated by Roadmap chromatin states.

Supplementary Figure 7
Nodal plots of enriched pathways and genesets of target genes associated with DNA methylation sentinels at FDR<0.01 in subcutaneous and visceral adipocytes.

Supplementary Figure 8
Genomic and functional annotation of enriched TF motifs in subcutaneous adipocytes.

Supplementary Figure 9
MR analyses of DNA methylation on adiposity, T2D, glycaemic and lipid traits.

Supplementary Figure 10
Adipocyte functional studies replication.

Supplementary Figure 11
Adipocyte functional studies off-target evaluation.

Supplementary Figure 12
Targeted methylation sequencing sensitivity analyses.

Supplementary Figure 13
Targeted methylation sequencing at the *PRRC2A* and *LIMD2* loci.

Supplementary Figure 14
TF motif-targeted methylation sequencing analyses.

Supplementary Figure 15
Targeted methylation sequencing at other loci of biological interest.

Supplementary Figure 16
CRISPR-activation design.

Supplementary Figure 17

CRISPR-activation results for *PRRC2A* and *LIMD2*.

Supplementary Figure 18
Human adipocyte RNA sequencing quality control analyses.

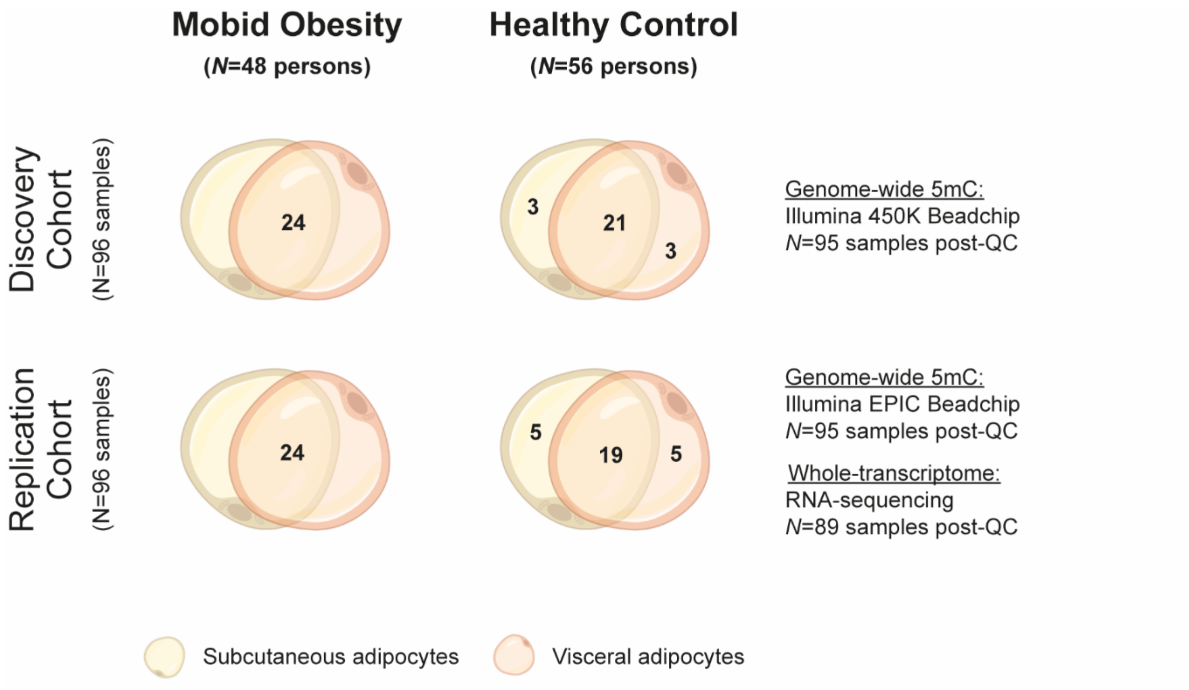

Supplementary Figure 1: Study Design.

We evaluated 192 adipocyte samples, half subcutaneous and half visceral, from people with extreme obesity and healthy controls in separate discovery and replication cohorts. In the discovery cohort, 24 obese people and 21 controls provided paired subcutaneous and visceral adipocyte samples. A further 3 controls provided just subcutaneous and 3 controls provided just visceral adipocytes. Similarly, in the replication cohort, 24 obese people and 19 controls provided paired subcutaneous and visceral adipocyte samples, with 10 further controls providing 5 subcutaneous and 5 visceral samples only. Genome-wide DNA methylation was characterised using the Illumina Methylation450 Beadchip in the 96 discovery samples, and the Illumina EPIC Beadchip in the 96 replication samples. Two methylation samples that failed quality control analyses were removed, one in each of the discovery and replication cohorts. RNA sequencing was carried out in the 96 replication samples, and 7 samples that failed sequencing or quality control analyses were removed. Created with BioRender.com.

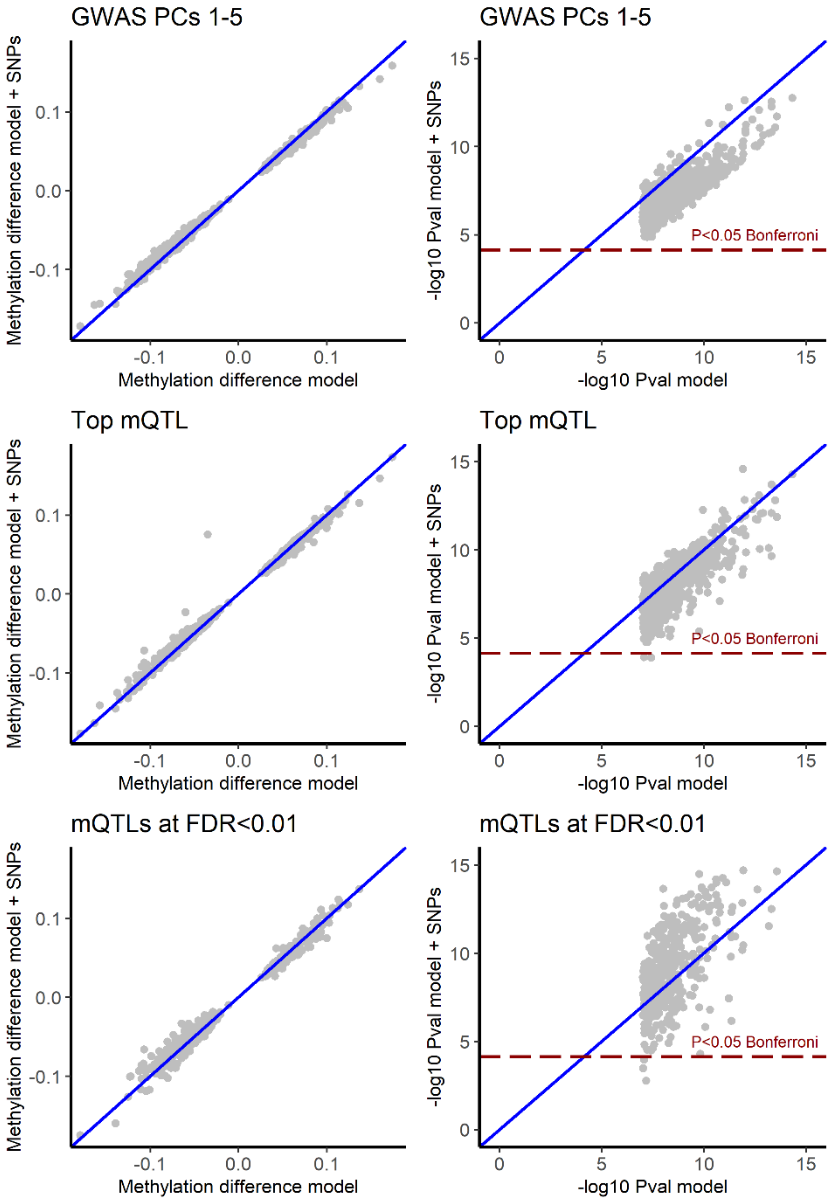

Supplementary Figure 2A: Effect of genetic factors on methylation-phenotype associations in subcutaneous adipocytes.

Associations between subcutaneous adipocyte DNA methylation and extreme obesity at each sentinel site were compared using linear regression analysis: i. the base model (X-axis: methylation difference model); and ii. the base adjusted for the effects of genetic variants (Y-axis: methylation difference model + SNPs, combined discovery and replication cohorts). Genetic factors did not systematically affect the relationships between DNA methylation and obesity, indicating that the majority of methylation-phenotype associations are likely to be environmentally driven. Top panels: adjustment for the first 5 principal components from PCA analysis of >8M genome-wide SNPs carried out in all study participants. Middle panels: adjustment for the genotype dose of the top cis-SNP (±500-kb) associated with change in methylation at each sentinel site (Top mQTL, additive model). Bottom panels: adjustment for the genotype dose of all cis-SNPs (±500-kb) associated with change in sentinel methylation at FDR<0.01 (additive model). Left panels: association effect sizes (betas). Right panels: -log10 pvalues for association. Blues lines: Null of no systematic difference. Dark red lines: Significance threshold (P<0.05, Bonferroni adjusted for the number of sentinels).

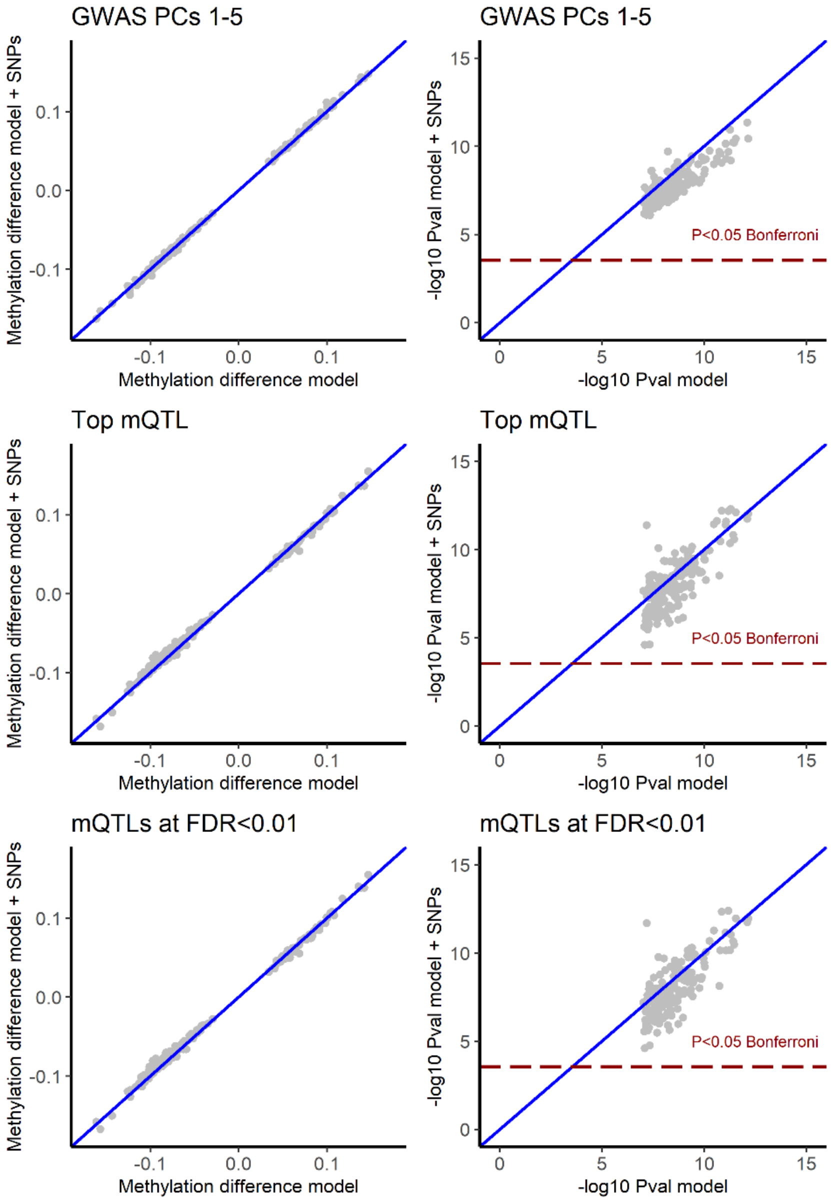

Supplementary Figure 2B: Effect of genetic factors on methylation-phenotype associations in visceral adipocytes.

Comparisons of associations between visceral adipocyte DNA methylation and extreme obesity at each sentinel site using linear regression analysis: i. the base model (X-axis: methylation difference model); and ii. the base adjusted for the effects of genetic variants (Y-axis: methylation difference model + SNPs, combined discovery and replication cohorts). As in subcutaneous adipocytes, genetic factors did not systematically affect DNA methylation-obesity associations, indicating underlying environmental origins. Top panels: adjusted for the first 5 principal components from PCA analysis of >8M SNPs carried out in all study participants. Middle panels: adjusted for the genotype dose of the top cis-SNP (±500-kb) associated with change in methylation at each sentinel site (Top mQTL, additive model). Bottom panels: adjusted for the genotype dose of all cis-SNPs (±500-kb) associated with change in sentinel methylation at FDR<0.01 (additive model). Left panels: association effect sizes (betas). Right panels: -log10 P values for association. Blues lines: Null of no systematic difference. Dark red lines: Significance threshold (P<0.05, Bonferroni adjusted for the number of sentinels).

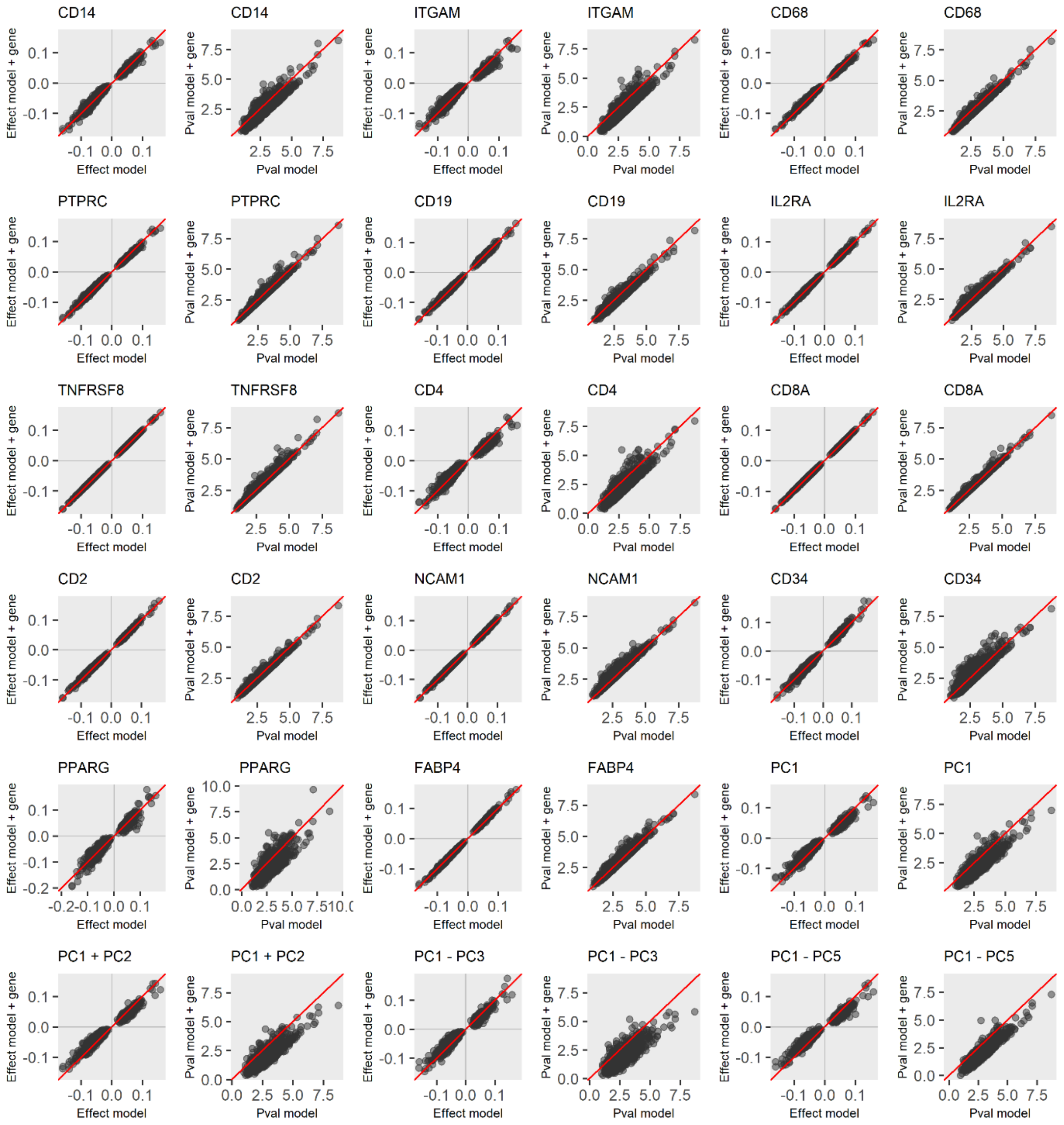

Supplementary Figure 3A: Evaluation of potential adipocyte impurity by contaminating gene expression in subcutaneous adipocytes.

Gene expression results from RNA sequencing were used to evaluate the effects of potential contaminating cells (impurity) on DNA methylation-obesity associations by comparing base models without and with adjustment for gene expression levels (subcutaneous, replication cohort). Effects of 14 individual genes were evaluated (12 potential contaminating and 2 control genes), as were principal components (PCs) from PCA analyses of all 12 potential contaminating cell genes. Analysed by linear regression. No systematic effects on DNA methylation-obesity associations at the 691 subcutaneous sentinel sites were observed. Red lines: Null of no systematic difference. Identifiers: *CD14* (monocyte/macrophage); *ITGAM* (CD11b, broad immune cell); *CD68* (monocyte/macrophage); *PTPRC* (CD45, broad immune cell); *CD19* (B lymphocyte); *IL2RA* (T lymphocyte); *TNFRSF8* (CD39, broad lymphocyte); *CD4* (T lymphocyte subtype); *CD8A* (T lymphocyte subtype); *CD2* (T lymphocyte and NK cell); *NCAM1* (NK cell); *CD34* (endothelial and precursor cell); *PPARG* (adipocyte); *FABP4* (adipocyte); PC1-PC5 (principal components from PCA analysis of the 12 immune and stromovascular genes). Analyses were carried out with variance stabilizing gene expression counts.

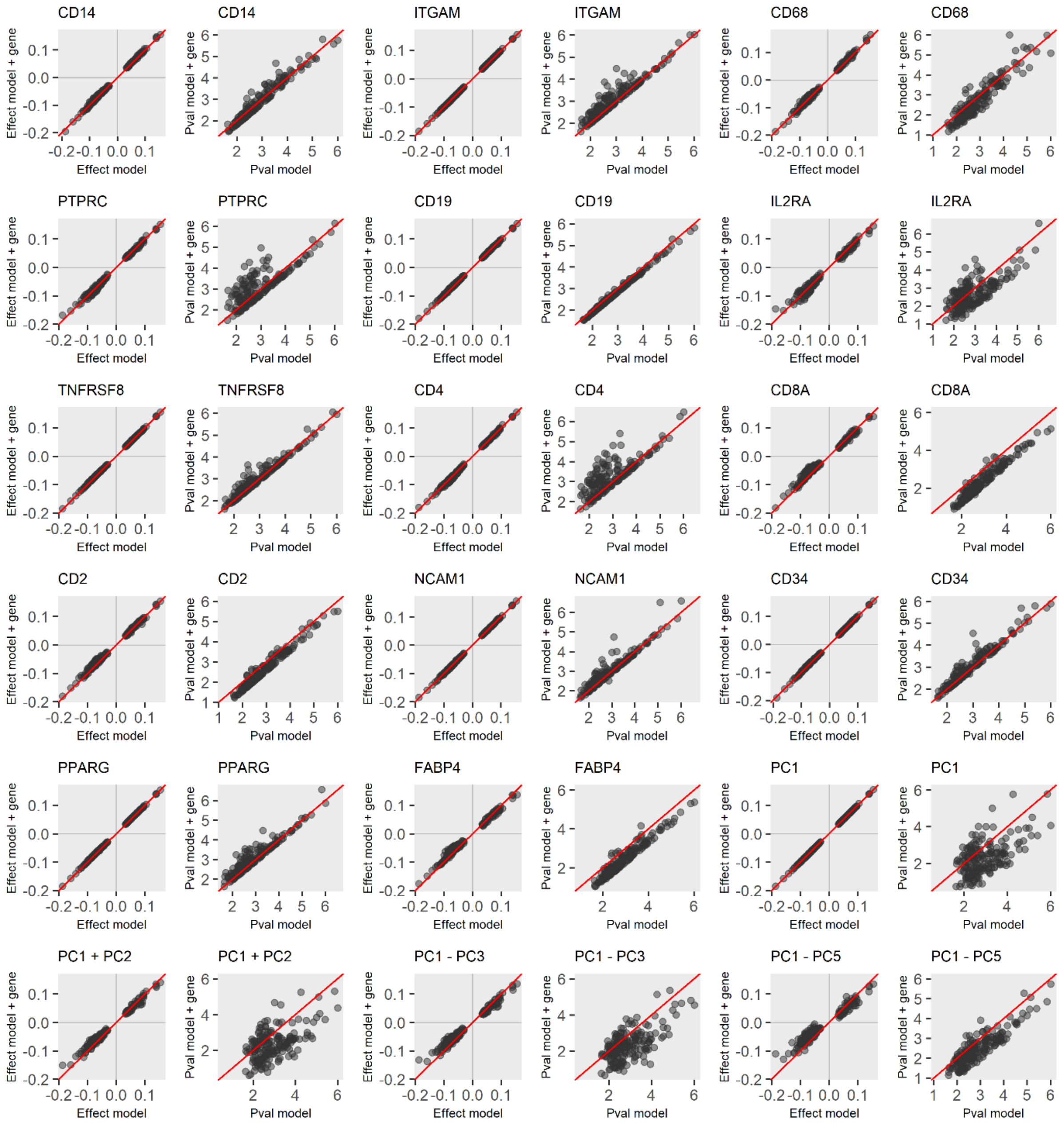

Supplementary Figure 3B: Evaluation of potential adipocyte impurity by contaminating gene expression in visceral adipocytes.

Effect of contaminating cell gene expression results from RNA sequencing (impurity) on DNA methylation-obesity associations (visceral replication cohort, 12 potential contaminating and 2 control genes, and principal components (PCs) from PCA analyses of all 12 potential contaminating cell genes). Analysed by linear regression. No systematic effects were observed at the 173 visceral sentinel sites. Red lines: Null of no systematic difference. Identifiers: *CD14* (monocyte/macrophage); *ITGAM* (CD11b, broad immune cell); *CD68* (monocyte/macrophage); *PTPRC* (CD45, broad immune cell); *CD19* (B lymphocyte); *IL2RA* (T lymphocyte); *TNFRSF8* (CD39, broad lymphocyte); *CD4* (T lymphocyte subtype); *CD8A* (T lymphocyte subtype); *CD2* (T lymphocyte and NK cell); *NCAM1* (NK cell); *CD34* (endothelial and precursor cells); *PPARG* (adipocyte); *FABP4* (adipocyte); PC1-PC5 (principal components from PCA analysis of the 12 immune and stromovascular genes). Analyses carried out with variance stabilizing gene expression counts.

A.

B.

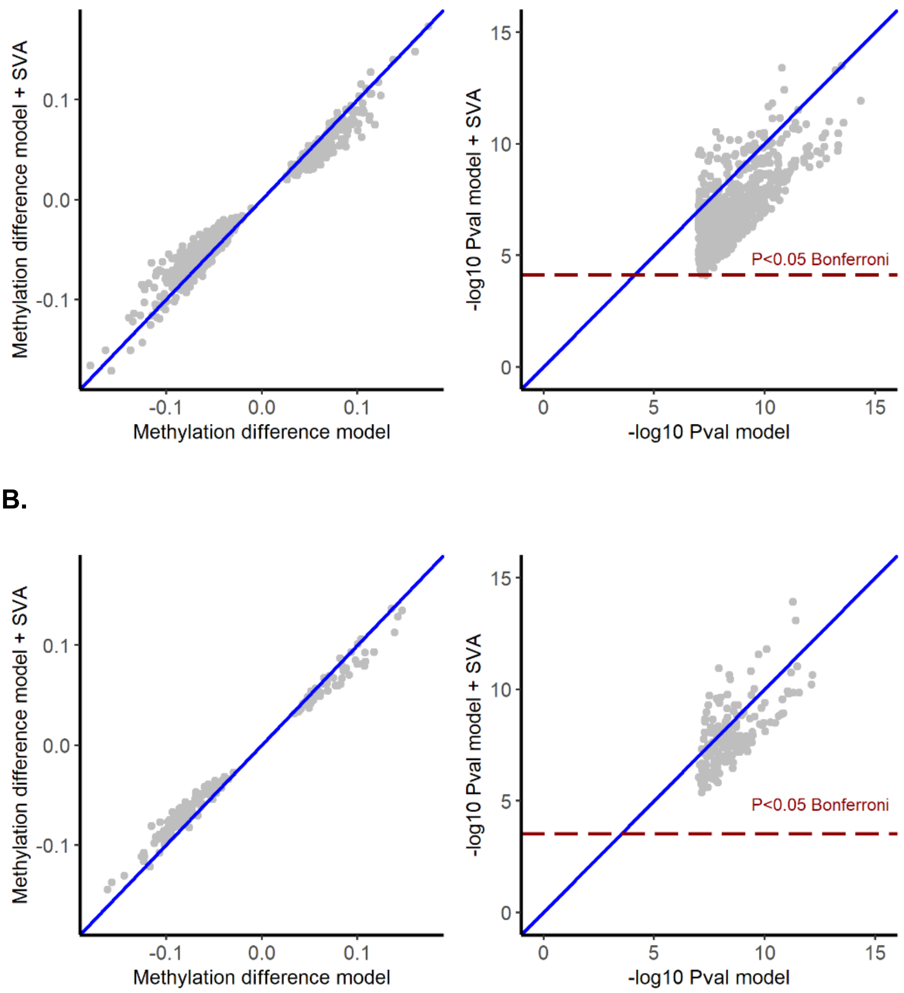

Supplementary Figure 4: Evaluation of potential adipocyte impurity by SVA.

Surrogate variable analysis was used to evaluate the effects of unknown confounders, in particular potential contaminating cell impurity, on associations between DNA methylation and extreme obesity in **A.** subcutaneous and **B.** visceral adipocytes (combined discovery and replication cohorts). Inclusion of SV1 and SV2 in the base linear regression models did not systematically alter methylation-phenotype effect sizes, and the majority of sentinels remained significantly associated with obesity after adjustment for SV1 and SV2 in the base model. Left panels: association betas. Right panels: -log10 P values. Analysed by linear regression. Blues lines: Null of no systematic difference. Dark red lines: Significance threshold (P<0.05, Bonferroni adjusted for the number of sentinels).

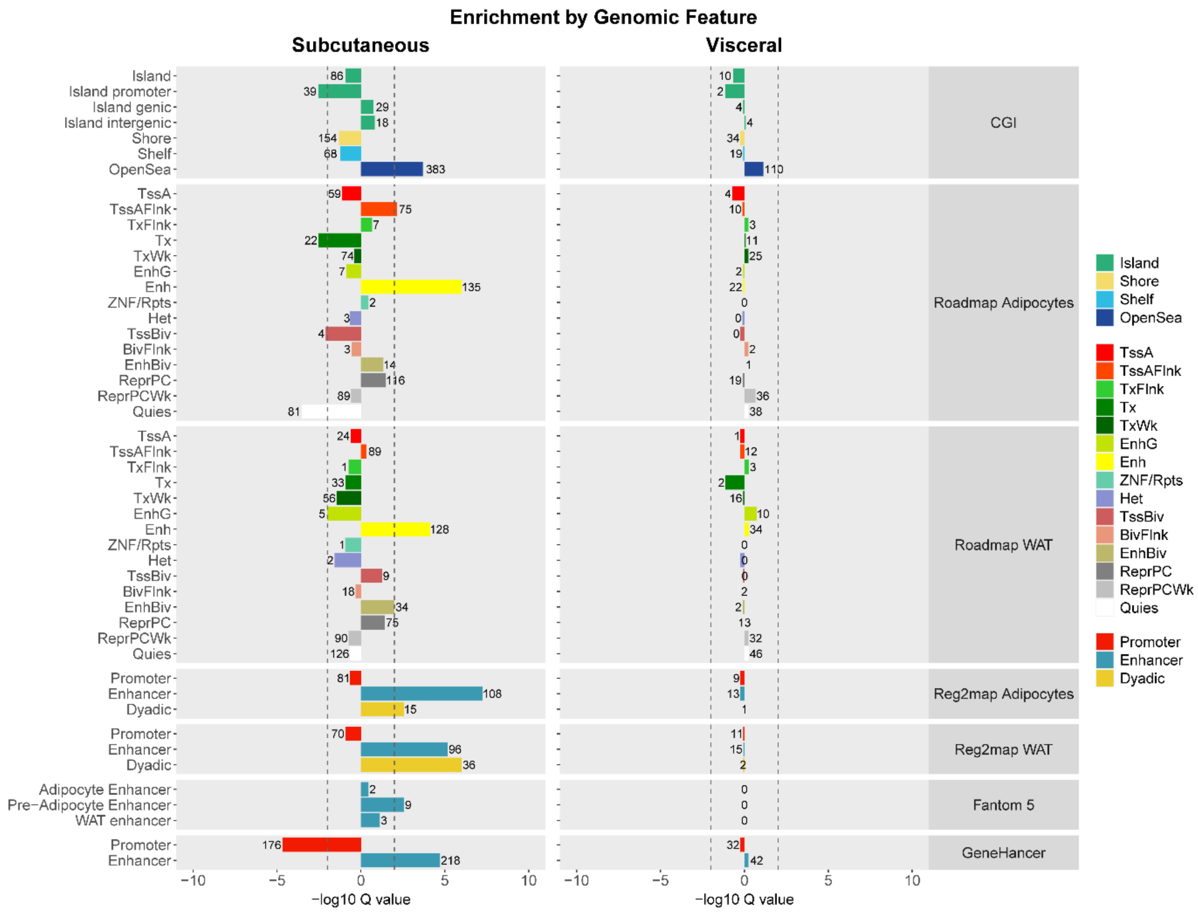

Supplementary Figure 5: Genomic annotation of subcutaneous and visceral sentinels.

Localisation of DNA methylation sentinels in subcutaneous and visceral adipocytes to functional/active genomic regions. Subcutaneous sentinels (N=691) were enriched in enhancers from various datasets and in CpG sparse genomic regions (open sea) but were underrepresented in promoter CGIs. Visceral sentinels (N=173) showed generally similar trends but did not reach statistical significance in any feature. CGI: CpG island track from UCSC. Presented as -log10 enrichment or depletion Q value, and number of observed counts, for each feature (Fisher's exact test, two-sided). Roadmap adipocytes and WAT: human adipocyte (E025) and adipose tissue nuclei chromatin states from the Roadmap epigenomics consortium (E063). Reg2map adipocytes and WAT: human adipocyte and adipose tissue regulatory features called using Roadmap and Encode epigenomes (-log10 P value ≥10). Fantom 5: human enhancer tracks called from Fantom 5 CAGE enhancer-promoter co-expression. GeneHancer: multifaceted human enhancer-target gene inference database.

TssA: Active TSS. TssAFlnk: Flanking Active TSS. TxFlnk: Transcr. at gene 5' and 3'. Tx: Strong transcription. TxWk: Weak transcription. EnhG: Genic enhancers. Enh: Enhancers. ZNF/Rpts: ZNF genes & repeats. Het: Heterochromatin. TssBiv: Bivalent/Poised TSS. BivFlnk: Flanking Bivalent TSS/Enh. EnhBiv: Bivalent Enhancer. ReprPC: Repressed PolyComb. ReprPCWk: Weak Repressed PolyComb. Quies: Quiescent/Low. Promoter: inferred promoter. Enhancer: inferred enhancer. Dyadic: inferred promoter/enhancer.

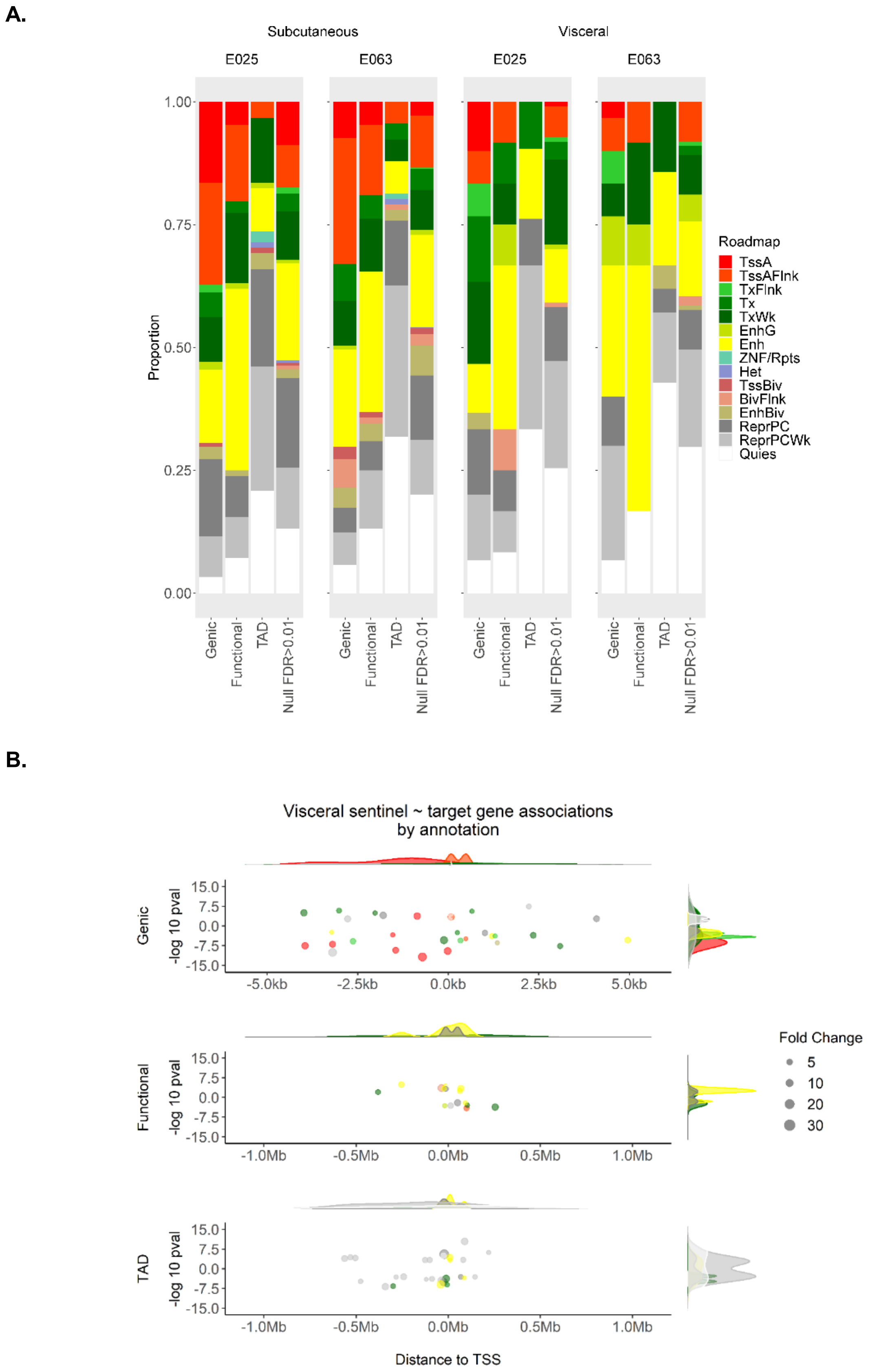

Supplementary Figure 6: DNA methylation-target gene associations in human subcutaneous and visceral adipocytes annotated by Roadmap chromatin states.

A. Subcutaneous and visceral adipocyte sentinel-target gene associations at FDR<0.01 grouped by annotation method and presented as proportion of associations in each Roadmap chromatin state. Genic: sentinel in a promoter, 5/3'UTR or exon. Functional: intronic/intergenic sentinel sharing a functional interaction with a distal target gene. TAD: intronic/intergenic sentinels and distal target genes sharing a topologically associated domain in human adipocytes. Null: methylation-target gene associations at FDR>0.01. E025: Human adipocytes. E063: human adipose tissue nuclei. All sentinel DNA methylation-target gene expression analyses were carried out in combined subcutaneous and visceral adipocyte samples.

TssA: Active TSS. TssAFlnk: Flanking Active TSS. TxFlnk: Transcr. at gene 5' and 3'. Tx: Strong transcription. TxWk: Weak transcription. EnhG: Genic enhancers. Enh: Enhancers. ZNF/Rpts: ZNF genes & repeats. Het: Heterochromatin. TssBiv: Bivalent/Poised TSS. BivFlnk: Flanking Bivalent TSS/Enh. EnhBiv: Bivalent Enhancer. ReprPC: Repressed PolyComb. ReprPCWk: Weak Repressed PolyComb. Quies: Quiescent/Low.

B. Visceral adipocyte sentinel-target gene associations at FDR<0.01 (analysed by fixed-effects linear regression), grouped by annotation method, presented as distance between the sentinel and its target gene TSS, and coloured by roadmap chromatin state (E025 adipocytes). Scatter plots of distance and -log 10 pvalue for association split according to direction of effect (methylation-expression). Density plots of each sentinel-target gene association relative to: i. distance (top); and ii. -log10 pvalue (right border). Fold change: expression fold change per unit change in methylation.

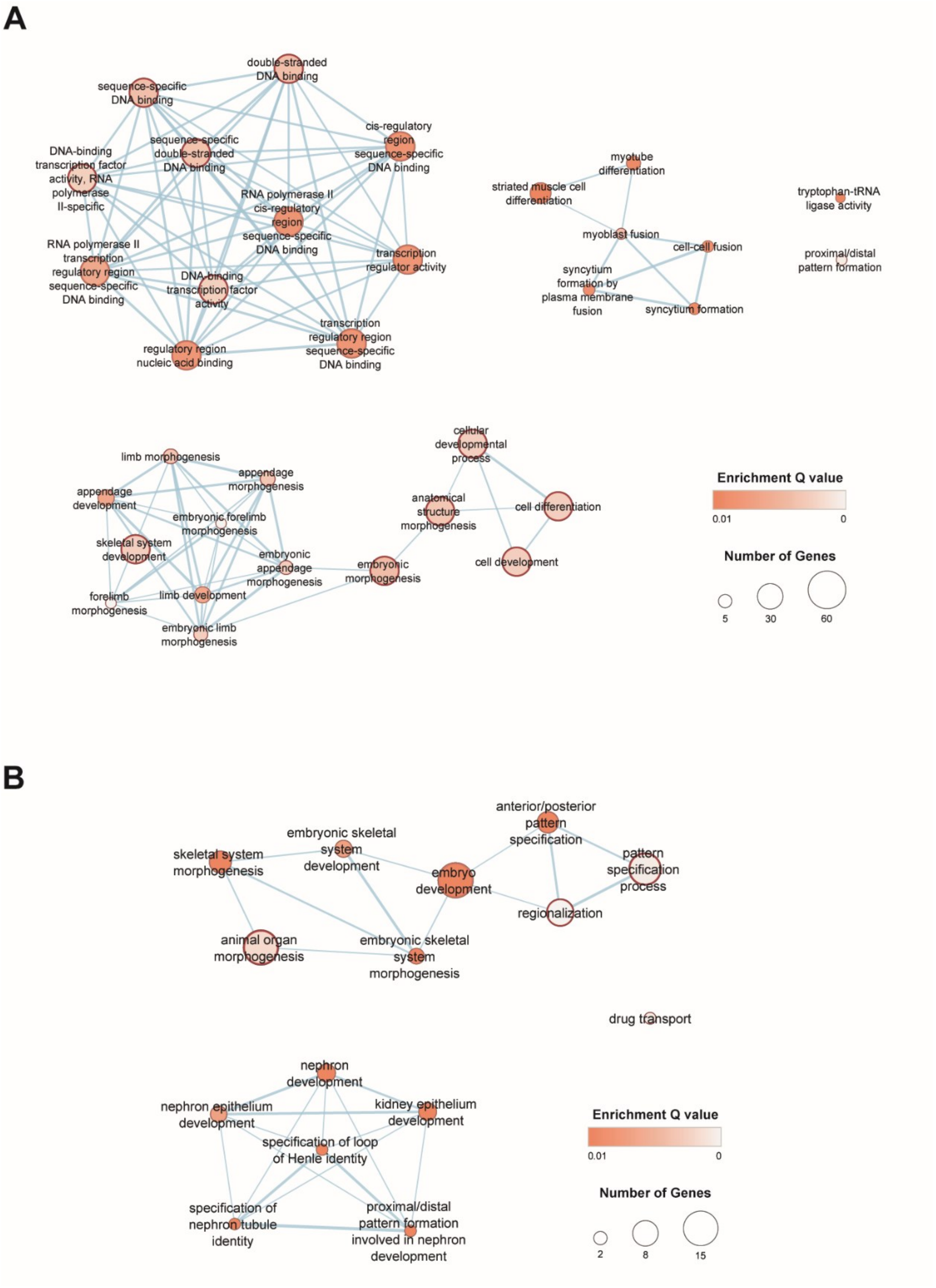

Supplementary Figure 7: Nodal plots of enriched pathways and genesets of target genes associated with DNA methylation sentinels at FDR<0.01 in subcutaneous and visceral adipocytes (gProfiler, Enrichment Map).

A. Subcutaneous: 33 enriched pathways/genesets clustered into transcriptional control and tissue development genesets, and cell, muscle and limb/morphogenesis subclusters. **B.** Visceral: 15 enriched pathways/genesets clustered into embryonic development/body patterning and nephronic development genesets. Coloured by enrichment Q value and sized by number of geneset members.

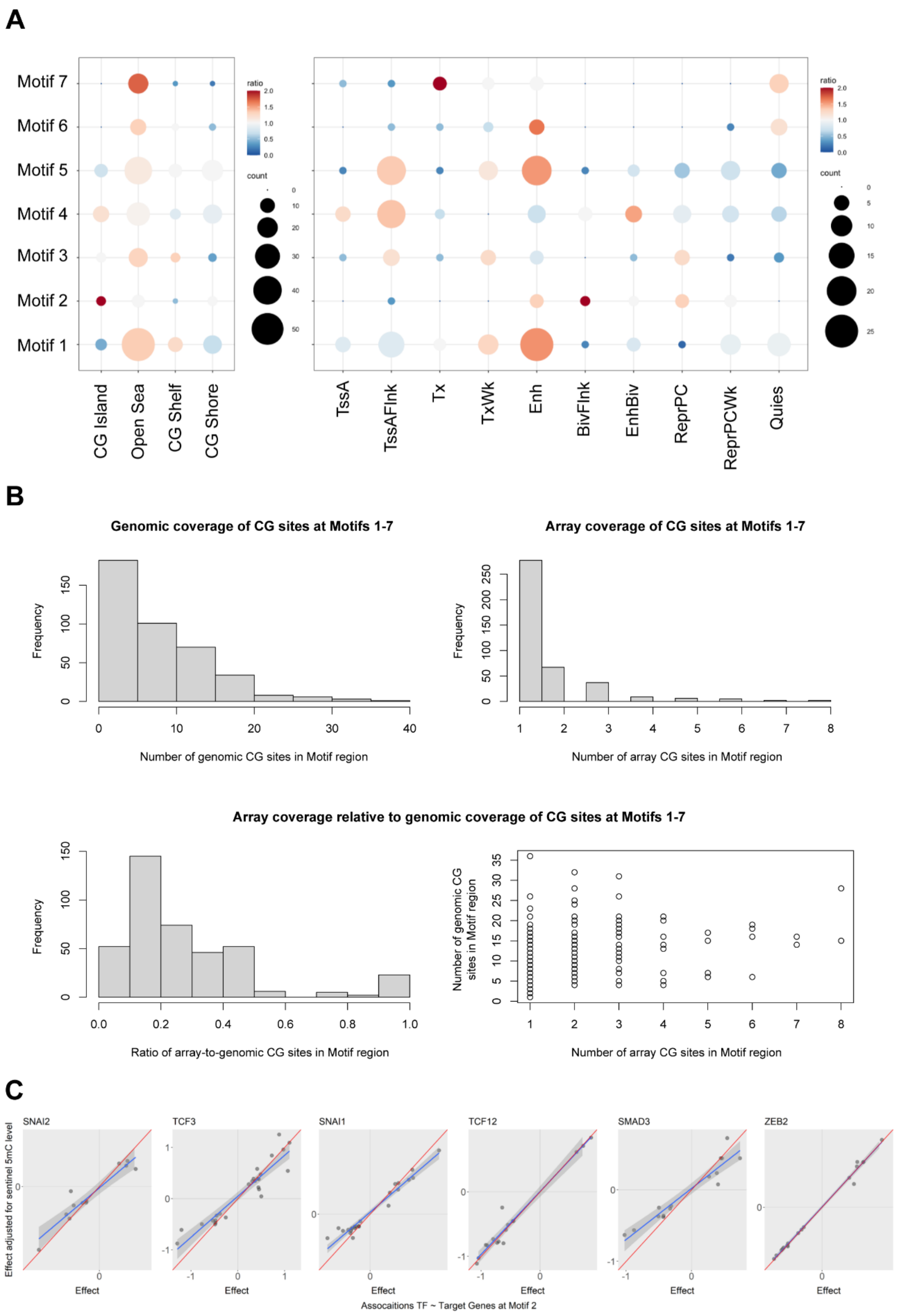

Supplementary Figure 8: Genomic and functional annotation of enriched TF motifs in subcutaneous adipocytes.

A. Genomic annotation of TF Motifs 1-7 in human CGIs and human adipocyte Roadmap chromatin states. Sized by the observed number of sentinel-motif pairs (count), and coloured by the ratio of

observed relative to the expected counts (permuted background). 5 of 15 Roadmap states with low observed counts in all 7 motifs are not presented.

TssA: Active TSS. TssAFlnk: Flanking Active TSS. Tx: Strong transcription. TxWk: Weak transcription. Enh: Enhancers. BivFlnk: Flanking Bivalent TSS/Enh. EnhBiv: Bivalent Enhancer. ReprPC: Repressed PolyComb. ReprPCWk: Weak Repressed PolyComb. Quies: Quiescent/Low.

B. Density of genomic CG sites at Motifs 1 to 7, and coverage of these CG sites on the Illumina HumanMethylation450 array. Top left panel: Frequency histogram of the number of genomic CG sites present within the +/-150-bp regions flanking Motifs 1-7. Top right panel: Frequency histogram of the number of CG sites present on the Illumina Humanmethylation450 array within the +/-150-bp regions flanking Motifs 1-7. Bottom panels: Coverage of genomic CG sites within the +/-150-bp regions flanking Motifs 1-7 by the Illumina Humanmethylation450 array, presented as the ratios (left) and counts (right).

C. Association between expression of 6 TFs predicted to bind to Motif 2, and expression of the predicted target genes of each sentinel methylation site corresponding to Motif 2 (combined subcutaneous and visceral adipocyte samples). X-axis: association betas without adjustment for sentinel methylation level. Y-axis: association betas with adjustment for sentinel methylation level. With regression line (blue) and 95% confidence intervals (dark grey). Adjustment for sentinel methylation level systematically impacted TF-target gene association relationships involving *SNAI2*, *TCF3*, *SNAI1* and *SMAD3*, but not *TCF12* and *ZEB2*.

A

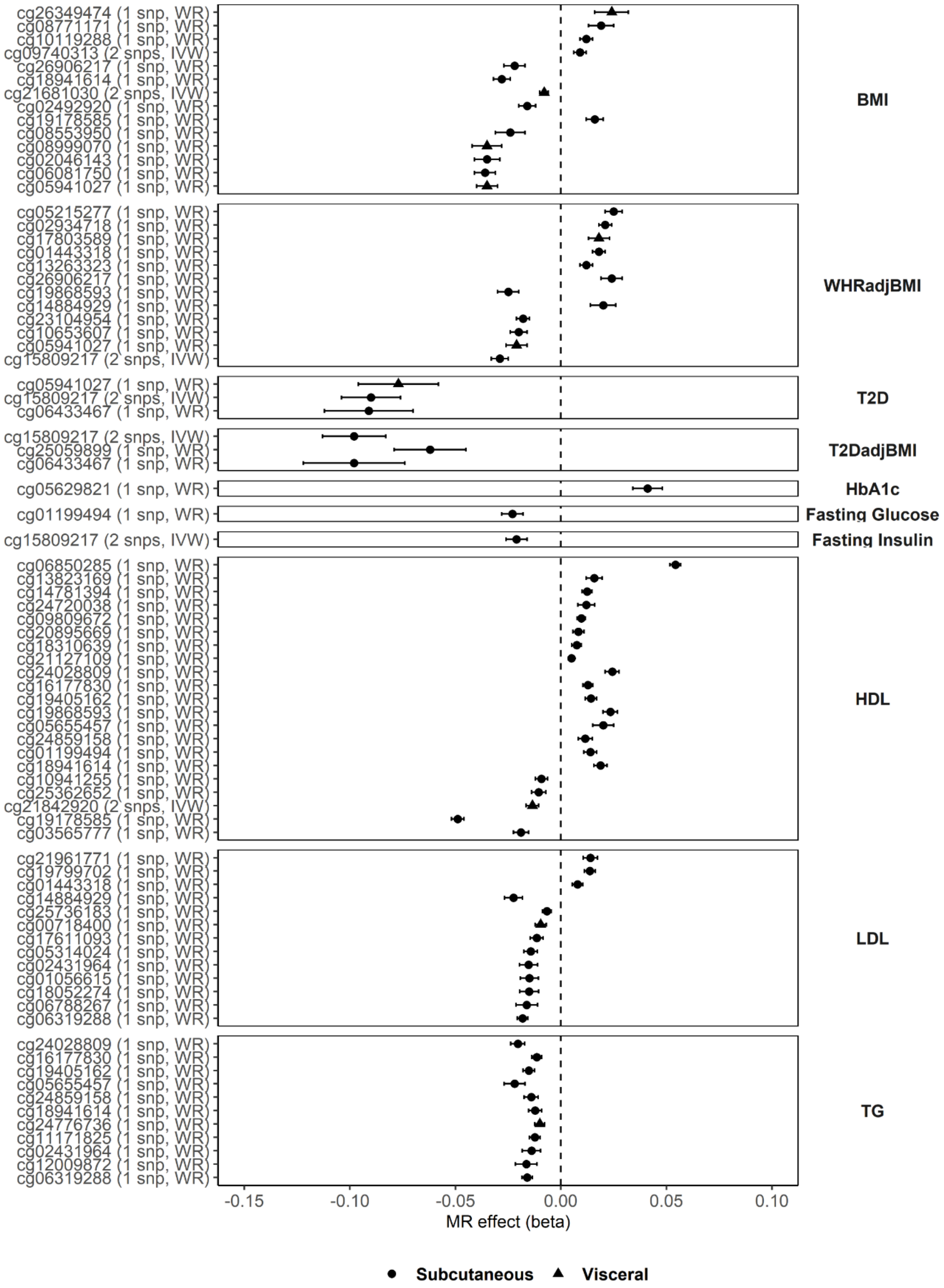
cg26349474 (1 snp, WR)
cg08771171 (1 snp, WR)
cg10119288 (1 snp, WR)
cg09740313 (2 snps, IVW)
cg26906217 (1 snp, WR)
cg18941614 (1 snp, WR)
cg21681030 (2 snps, IVW)
cg02492920 (1 snp, WR)
cg19178585 (1 snp, WR)
cg08553950 (1 snp, WR)
cg08999070 (1 snp, WR)
cg02046143 (1 snp, WR)
cg06081750 (1 snp, WR)
cg05941027 (1 snp, WR)
BMI
cg05215277 (1 snp, WR)
cg02934718 (1 snp, WR)
cg17803589 (1 snp, WR)
cg01443318 (1 snp, WR)
cg13263323 (1 snp, WR)
cg26906217 (1 snp, WR)
cg19868593 (1 snp, WR)
cg14884929 (1 snp, WR)
cg23104954 (1 snp, WR)
cg10653607 (1 snp, WR)
cg05941027 (1 snp, WR)
cg15809217 (2 snps, IVW)
WHRadjBMI
cg05941027 (1 snp, WR)
cg15809217 (2 snps, IVW)
cg06433467 (1 snp, WR)
T2D
cg15809217 (2 snps, IVW)
cg25059899 (1 snp, WR)
cg06433467 (1 snp, WR)
T2DadjBMI
cg05629821 (1 snp, WR)
HbA1c
cg01199494 (1 snp, WR)
Fasting Glucose
cg15809217 (2 snps, IVW)
Fasting Insulin
cg06850285 (1 snp, WR)
cg13823169 (1 snp, WR)
cg14781394 (1 snp, WR)
cg24720038 (1 snp, WR)
cg09809672 (1 snp, WR)
cg20895669 (1 snp, WR)
cg18310639 (1 snp, WR)
cg21127109 (1 snp, WR)
cg24028809 (1 snp, WR)
cg16177830 (1 snp, WR)
cg19405162 (1 snp, WR)
cg19868593 (1 snp, WR)
cg05655457 (1 snp, WR)
cg24859158 (1 snp, WR)
cg01199494 (1 snp, WR)
cg18941614 (1 snp, WR)
cg10941255 (1 snp, WR)
cg25362652 (1 snp, WR)
cg21842920 (2 snps, IVW)
cg19178585 (1 snp, WR)
cg03565777 (1 snp, WR)
HDL
cg21961771 (1 snp, WR)
cg19799702 (1 snp, WR)
cg01443318 (1 snp, WR)
cg14884929 (1 snp, WR)
cg25736183 (1 snp, WR)
cg00718400 (1 snp, WR)
cg17611093 (1 snp, WR)
cg05314024 (1 snp, WR)
cg02431964 (1 snp, WR)
cg01056615 (1 snp, WR)
cg18052274 (1 snp, WR)
cg06788267 (1 snp, WR)
cg06319288 (1 snp, WR)
LDL
cg24028809 (1 snp, WR)
cg16177830 (1 snp, WR)
cg19405162 (1 snp, WR)
cg05655457 (1 snp, WR)
cg24859158 (1 snp, WR)
cg18941614 (1 snp, WR)
cg24776736 (1 snp, WR)
cg11171825 (1 snp, WR)
cg02431964 (1 snp, WR)
cg12009872 (1 snp, WR)
cg06319288 (1 snp, WR)
TG
-0.15
-0.10
-0.05
0.00
0.05
0.10
MR effect (beta)
Subcutaneous
Visceral

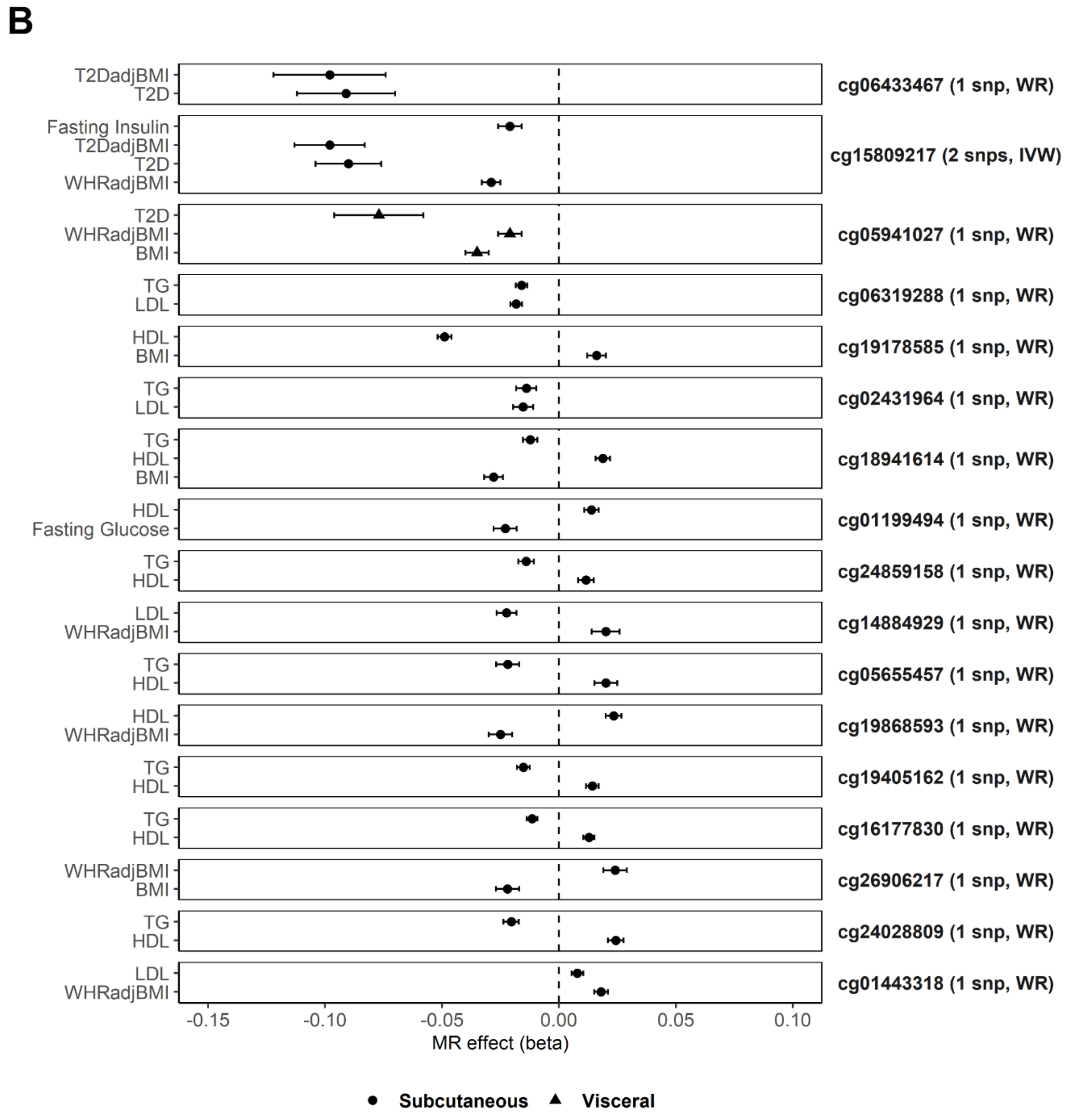

Supplementary Figure 9: MR analyses of DNA methylation on adiposity, T2D, glycaemic and lipid traits.

Forest plots of the effect sizes of subcutaneous and visceral adipocyte sentinels causally associated with human obesity, central adiposity, T2D, glycaemic and lipid traits through two sample MR in adipocytes (FDR<0.01 in both MR causal and Steiger directionality tests). **A.** Grouped by trait. **B.** Grouped by methylation site (limited to methylation sites with >1 trait association).

Centre values mark effect size estimates (MR beta) and error bars show the 95% confidence intervals. MR causal tests: Wald Ratio (WR) for single SNP instrumental variable (IV); Inverse Variance Weighted (IVW) for >1 SNP IV. BMI: body mass index as a measure of obesity (GIANT, N≤795,640). WHRadjBMI: Waist-hip ratio adjusted for BMI as a measure of central adiposity (GIANT N≤694,649). T2D and T2D adjusted for BMI as measures of T2D risk (DIAGRAM, N≤231,422). Fasting glucose and insulin (MAGIC, N≤138,589) and HbA1c (MAGIC, N≤159,940) as measures of glycaemic traits linked to T2D. HDL: High-density lipoprotein cholesterol; LDL: Low-density lipoprotein cholesterol; TG: Triglycerides (Global Lipids Genetics Consortium N≤1,654,960).

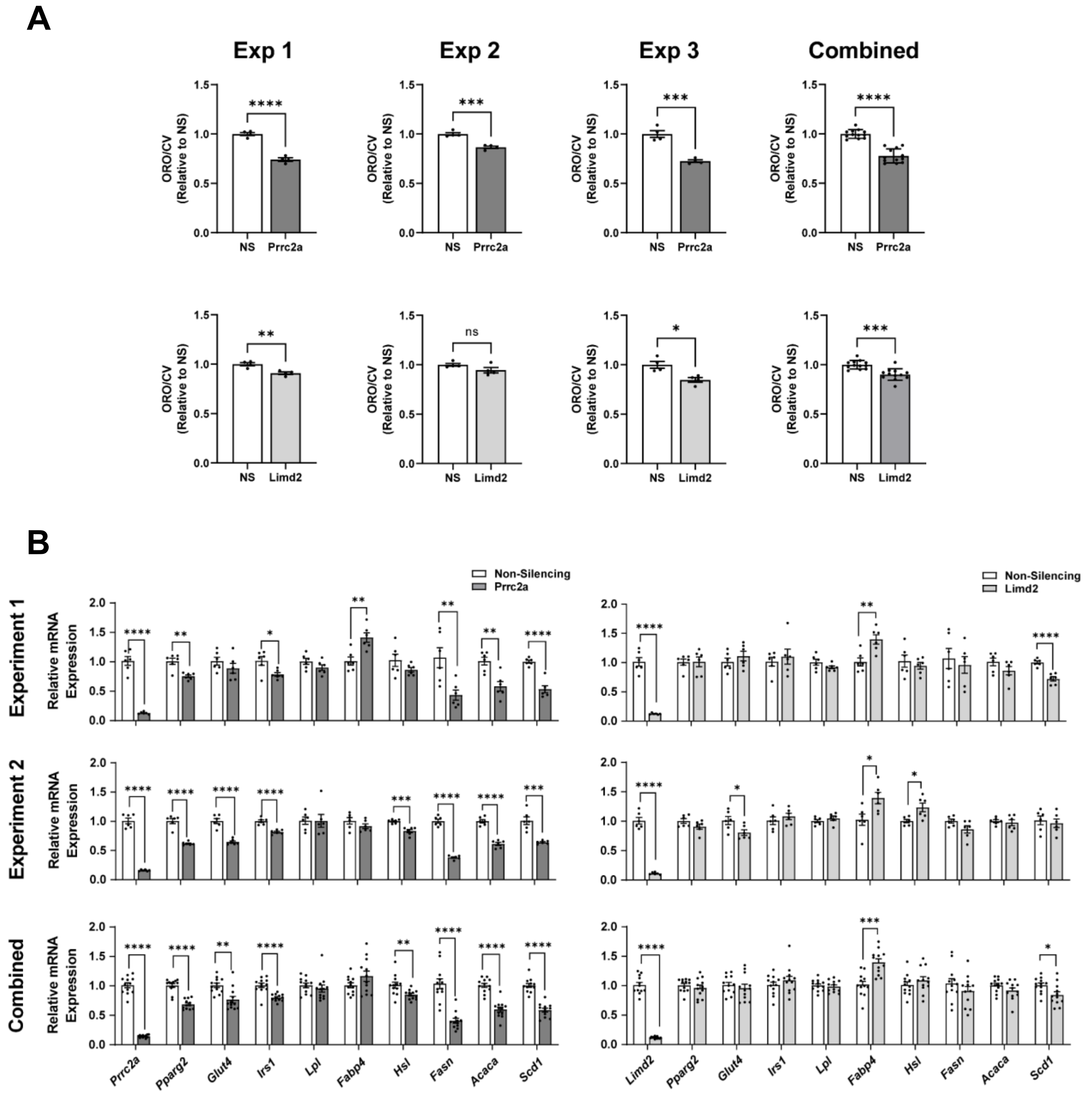

Supplementary Figure 10: Adipocyte functional studies replication.

Studies of lipid accumulation and expression of key genes involved in adipocyte metabolism at day 6 of differentiation, in 3T3-L1 adipocytes transfected with siRNA against *Prrc2a*, *Limd2* or non-silencing (NS) control at day 2 after initiation of differentiation.

A. Effects of *Prrc2a* and *Limd2* knockdown on lipid accumulation, assessed using spectrophotometry of eluted ORO stain normalised for cell number (crystal violet, CV, N=4 independent samples per condition). Results are shown for three replicate experiments (Exp. 1-3) and for the combined replicates. All values are presented as mean ± SEM relative to NS control. *Prrc2a* P=3.5x10-5 (Exp 1), P=0.00042 (Exp 2), P=0.00032 (Exp 3) and P=4.4x10-9 (combined), *Limd2* P=0.0047 (Exp 1), P=0.013 (Exp 3) and P=0.00010 (combined).

B. Effects of *Prrc2a* and *Limd2* knockdown on the expression of genes involved in adipocyte differentiation (*Pparg*), insulin signalling (*Glut4*, *Irs1*), lipid uptake (*Lpl*), lipid storage (*Fasn*, *Acaca*, *Scd1*) and lipid mobilisation (*Fabp4*, *Hsl*, N=6 independent samples per condition). Real-time qPCR values were normalised to housekeeping genes (*Nono*, *Ywhaz*). Results are shown for two replicate experiments (Experiment 1-2, with individual data points) and for the combined replicates.

All values are presented as mean ± SEM relative to NS control. *P<0.05, **P<0.01, ***P<0.001, ****P<0.0001 versus NS control (Student's t-test, two-sided); source data are provided in the Source Data file.

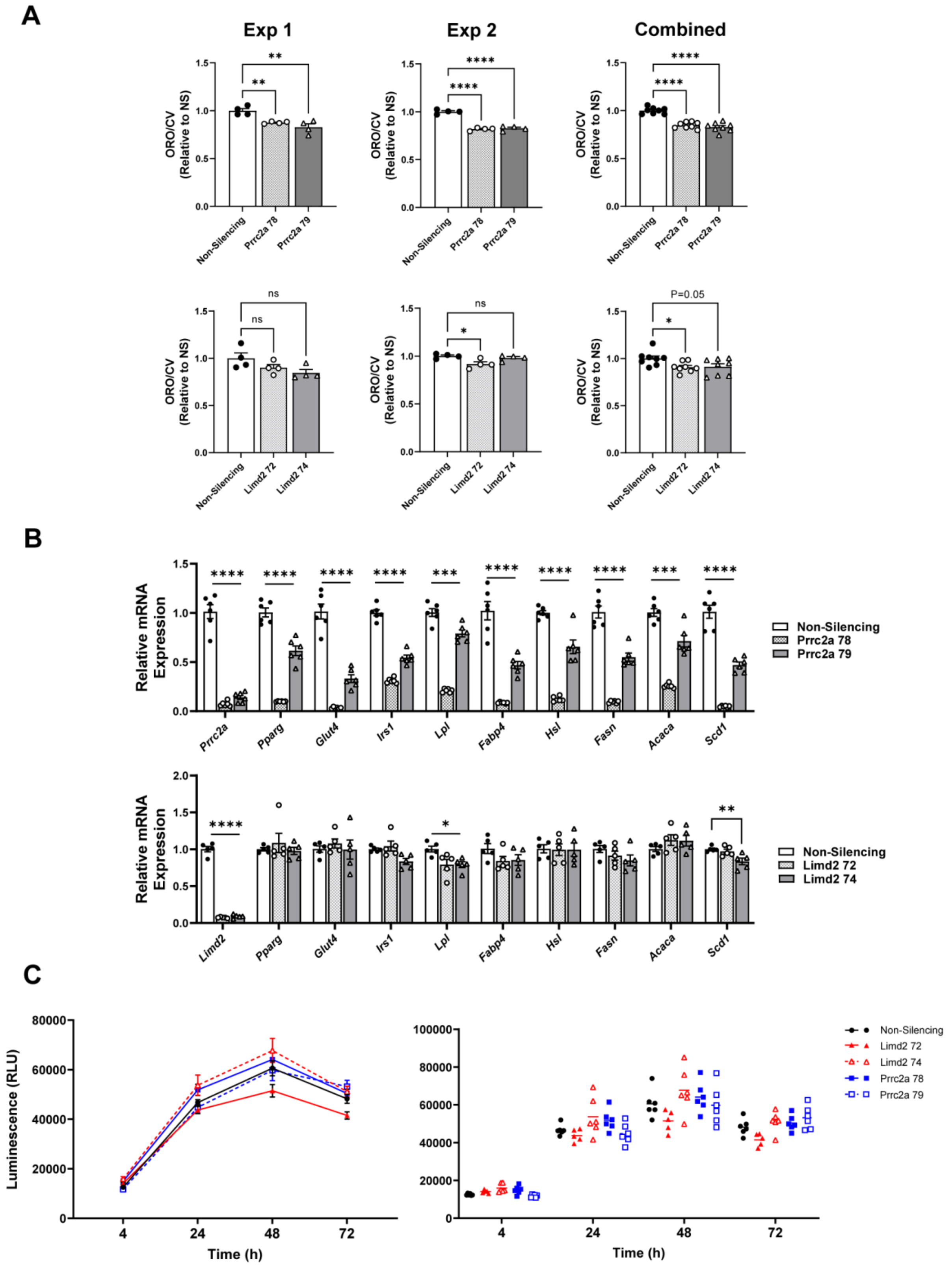

Supplementary Figure 11: Adipocyte functional studies off-target evaluation.

Studies of lipid accumulation, gene expression and cell viability in 3T3-L1 adipocytes transfected with two distinct siRNA against *Prrc2a* (78, 79), *Limd2* (72, 74) or non-silencing (NS) control. This approach was used to confirm the absence of siRNA mediated off-target effects, as the distinct siRNA were designed to share no sequence, and thus target different regions of the *Prrc2a* and *Limd2* mRNA.

A. Effects of *Prrc2a* and *Limd2* knockdown using distinct siRNA on lipid accumulation, assessed using spectrophotometry of eluted ORO stain normalised for cell number (crystal violet, CV, N=4 per condition). 3T3-L1 adipocytes were transfected at day 2 and ORO was measured at day 6 after initiation of differentiation. Results are shown for two replicate experiments (Exp. 1-2) and for the combined replicates. *Prrc2a 78* P=0.0094 (Exp 1), P<0.0001 (Exp 2) and P<0.0001 (combined), *Prrc2a 79* P=0.0012 (Exp 1), P<0.0001 (Exp 2) and P<0.0001 (combined), *Limd2 72* P=0.015 (Exp 2), P=0.040 (combined), *Limd2 74* P=0.051 (combined). All values are presented as mean ± SEM. ORO is shown relative to NS control. *P<0.05, **P<0.01, ***P<0.001, ****P<0.0001 (One-Way ANOVA test, Dunnett's test multiple comparisons); source data are provided in the Source Data file.

B. Effects of *Prrc2a* and *Limd2* siRNA silencing using distinct siRNA on the expression of genes involved in adipocyte differentiation (*Pparg*), insulin signalling (*Glut4*, *Irs1*), lipid uptake (*Lpl*), lipid storage (*Fasn*, *Acaca*, *Scd1*) and lipid mobilisation (*Fabp4*, *Hsl*, N=6 independent samples per condition *Prrca2,* N=5 independent samples per condition *Limd2*). Real-time qPCR values were normalised to housekeeping genes (*Nono*, *Ywhaz*). 3T3-L1 adipocytes were transfected at day 2 and gene expression was measured at day 6 after initiation of differentiation. All values are presented as mean ± SEM. Gene expression is shown relative to NS control. *P<0.05, **P<0.01, ***P<0.001, ****P<0.0001 (One-Way ANOVA test, Dunnett's test multiple comparisons); source data are provided in the Source Data file.

C. Effects of *Prrc2a* and *Limd2* siRNA silencing using distinct siRNA on cell viability measured using the RealTime-Glo™ luminescence assay (Relative Light Units, RLU) to quantify metabolic activity in live/healthy cells at 4-hr, 24-hr, 48-hr and 72-hr after siRNA treatment. N=6 per condition in undifferentiated 3T3-L1 adipocytes. All values are presented as mean ± SEM (Two-Way repeated measures ANOVA test, Dunnett's test multiple comparisons); source data are provided in the Source Data file.

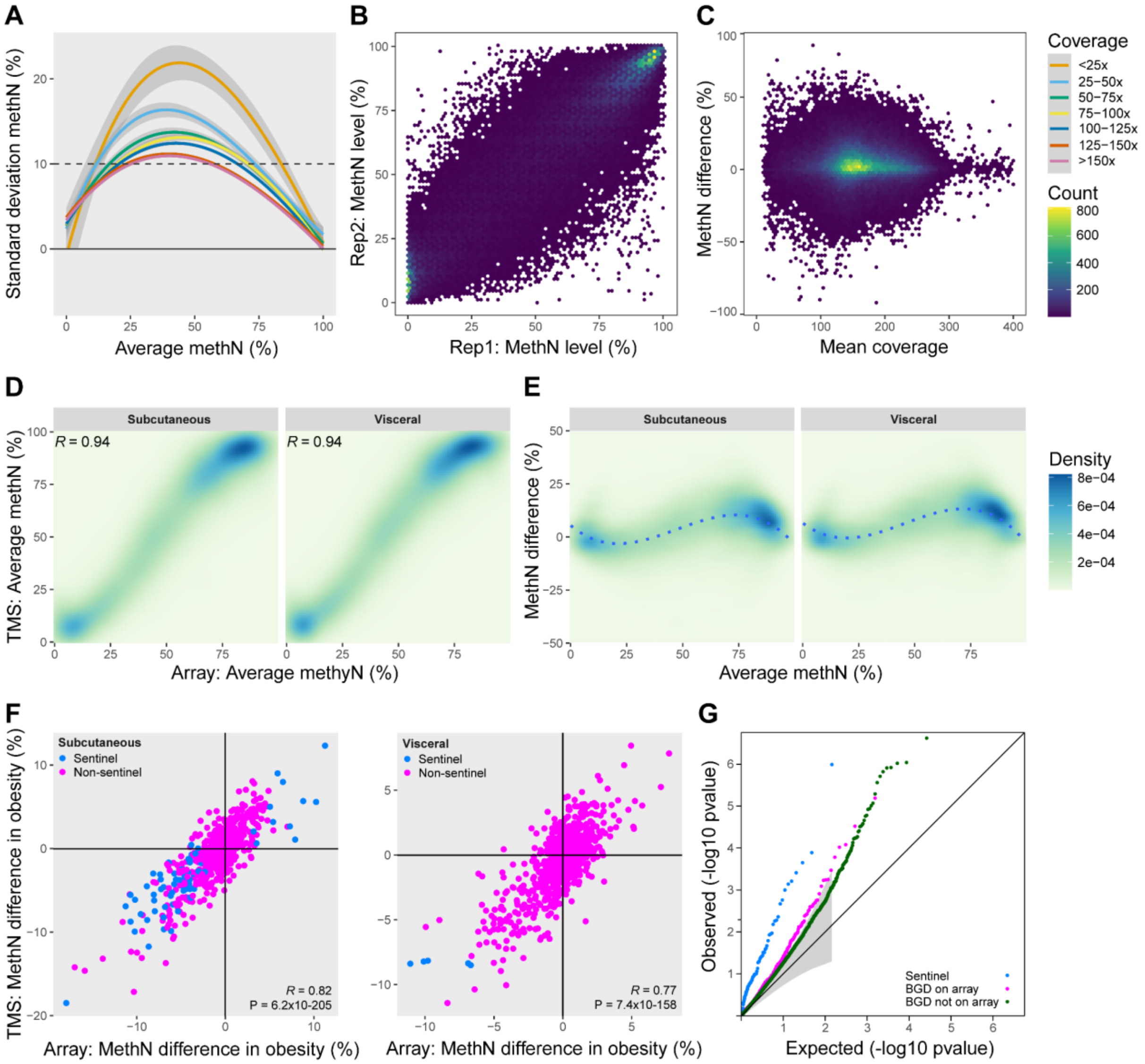

Supplementary Figure 12: Targeted methylation sequencing sensitivity analyses.

Precision and concordance analyses of 8 samples sequenced in replicate. **A.** Mean and standard deviation of methylation measurements (%) at paired sites (N=~13K) grouped by depth of coverage. Targeted methylation sequencing even at >150x coverage lacked the precision of array-based and whole-methylome sequencing (when compared to our previous work[120]). **B.** Concordance of methylation measurements (%) at paired sites. Rep1 and Rep2: Replicate 1 (pilot) and Replicate 2 (main). **C.** Differences in methylation measurements (%) between replicates relative to mean coverage at paired sites.

Comparisons of methylation measurements using Array (Illumina HumanMethylation450 and EPIC BeadChips combined) and targeted methylation sequencing (TMS) platforms at 839 sites present in both datasets. **D-E.** Methylation measurements (%) on different platforms correlated strongly but showed platform specific biases, similar to those observed in whole-methylome sequencing results[119].

Associations with obesity in 89 subcutaneous and visceral adipocyte samples evaluated by targeted methylation sequencing (TMS). **F.** Effect sizes at sentinel and non-sentinel sites present in targeted sequencing and array datasets (combined discovery and replication cohorts, %-methylation difference in obesity) were highly concordant (Pearson correlation coefficient (R), two-sided). **G.** Sentinel methylation sites were enriched for association with obesity in targeted methylation sequencing data compared to: i. non-sentinel sites present on the array; and ii. non-sentinel sites not present on the

array. Observed: observed association P values in TMS results (linear regression). Expected: expected association P values based on the null hypothesis of no associations.

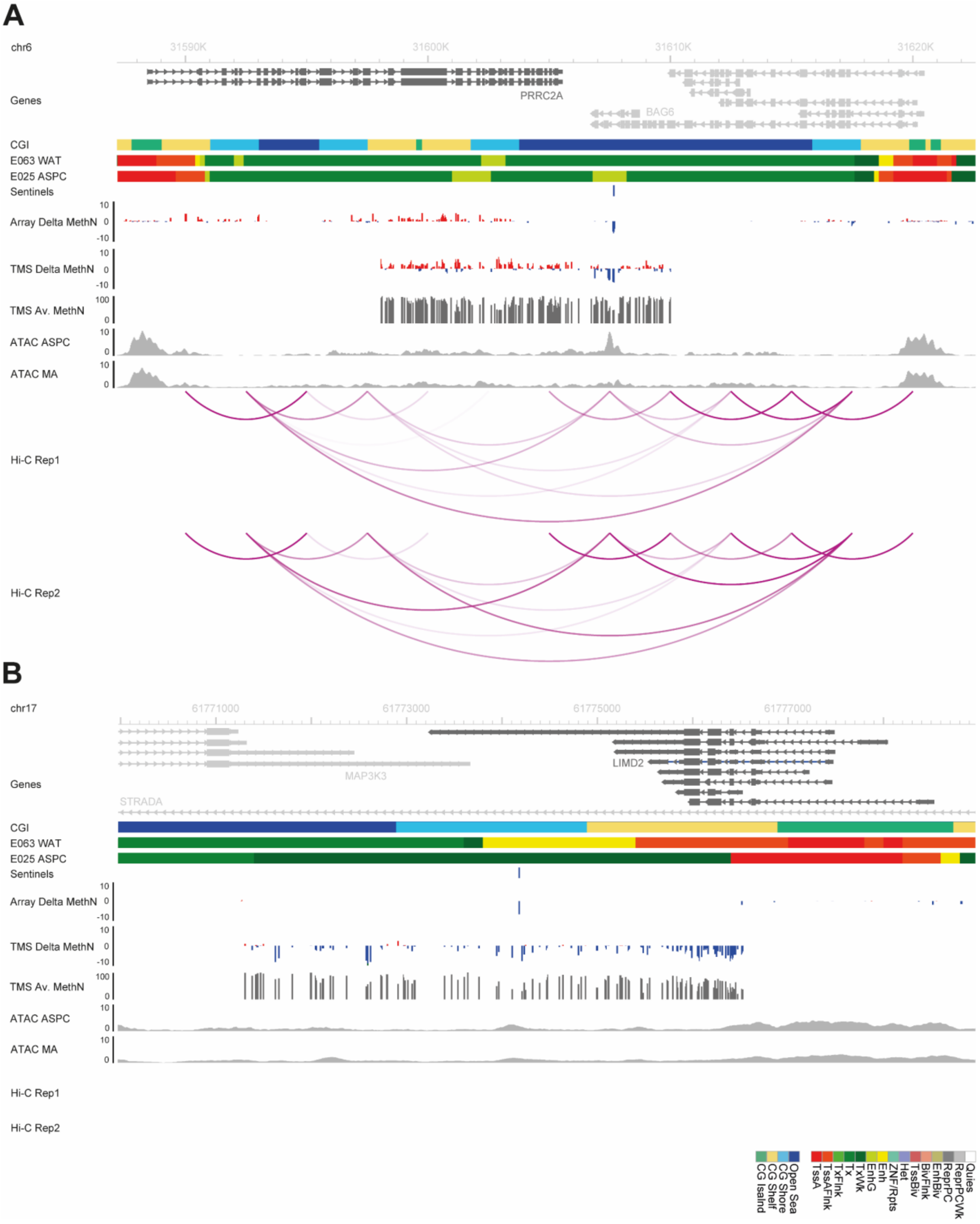

Supplementary Figure 13: Targeted methylation sequencing at the *PRRC2A* and *LIMD2* loci.

A. Targeted methylation sequencing at the *PRRC2A* locus (subcutaneous adipocytes, N=43) replicated the sentinel association and identified multiple differentially hypomethylated sites associated with obesity within a preadipocyte-specific open chromatin peak and intronic enhancer sharing functional connections with the *PRRC2A* gene.

B. Targeted methylation sequencing at the *LIMD2* locus (visceral adipocytes, N=46) also replicated the sentinel association and revealed an extended differentially methylated region covering a proximal enhancer and multiple *LIMD2* exons and splice sites.

CGI: UCSC CpG islands. E063 and E025: Roadmap adipose and adipocyte chromatin states. Sentinel: Sentinel methylation site in combined discovery and replication data. Array Delta MethyN: Difference in methylation (%) in obesity in combined discovery and replication data (red higher, blue lower). TMS Delta MethyN: Difference in methylation (%) in obesity in targeted methylation sequencing data (red higher, blue lower). TMS Av. MethyN: Mean methylation level (%) in targeted methylation sequencing data. ATAC ASPC: ATAC sequencing of undifferentiated SGBS human preadipocytes (GEO: GSE110734[121]). ATAC MA: ATAC sequencing of differentiated SGBS mature human adipocytes (GEO: GSE110734[121]). Hi-C Rep1 and Rep2: Human adipocyte Hi-C functional connectivity maps at Day 3 of differentiation in two independent replicates (GEO: GSE109924[58]).

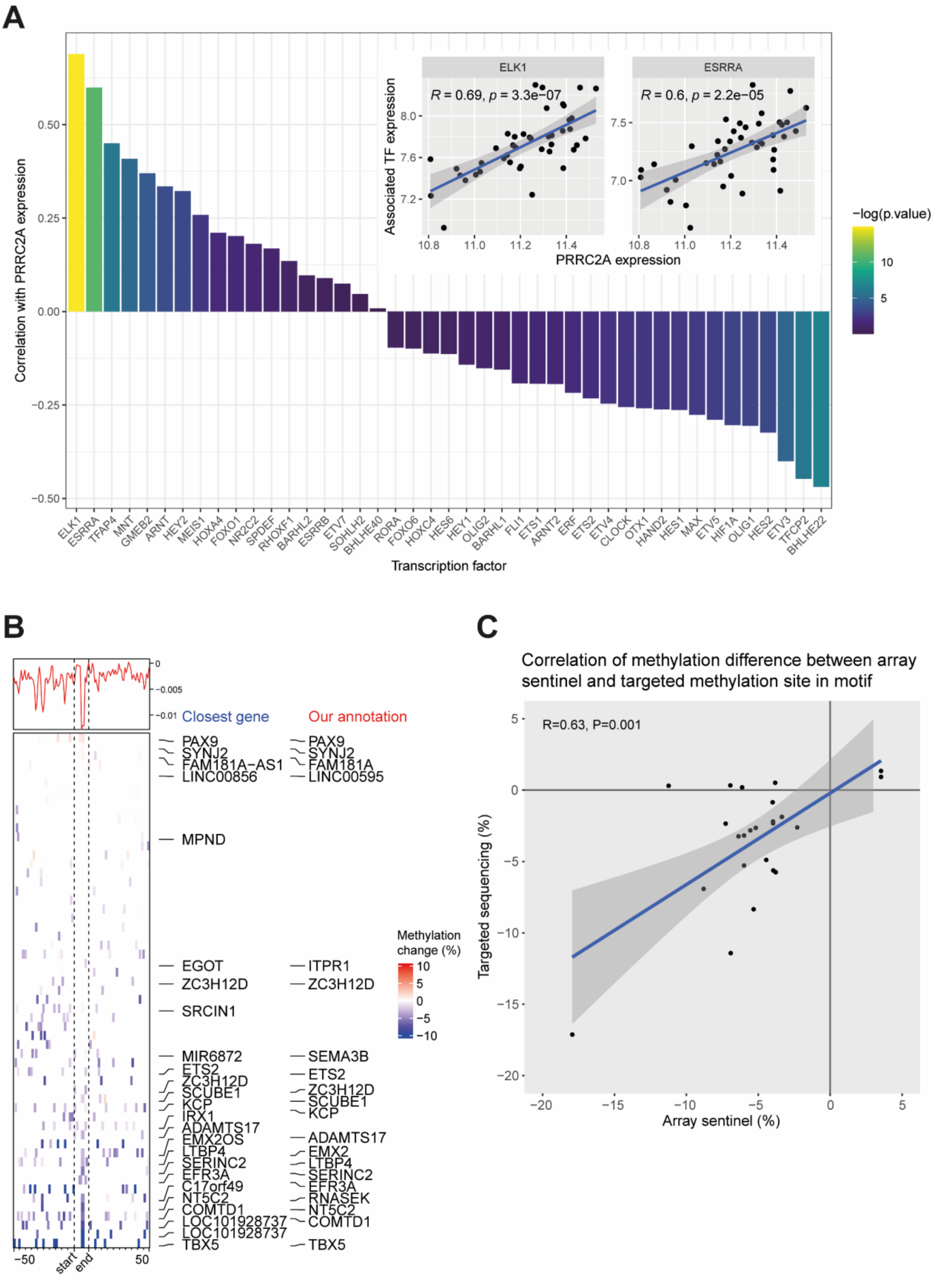
A
Correlation with PRRC2A expression
0.50
0.25
0.00
−0.25
−0.50
Transcription factor
ELK1 ESRRA TFAP4 MNT GMEB2 ARNT HEY2 MEIS1 HOXA4 FOXO1 NR2C2 SPDEF RHOXF1 BARHL2 ESRRB ETV7 SOHLH2 BHLHE40 RORA FOXO6 HOXC4 HES6 HEY1 OLIG2 BARHL1 FLI1 ETS1 ARNT2 ERF ETS2 ETV4 CLOCK OTX1 HAND2 HES1 MAX ETV5 HIF1A OLIG1 HES2 ETV3 TFCP2 BHLHE22
ELK1
ESRRA
R = 0.69, p = 3.3e−07
R = 0.6, p = 2.2e−05
Associated TF expression
PRRC2A expression
−log(p.value)
10
5
B
Closest gene
Our annotation
PAX9
SYNJ2
FAM181A−AS1
LINC00856
MPND
EGOT
ZC3H12D
SRCIN1
MIR6872
ETS2
ZC3H12D
SCUBE1
KCP
IRX1
ADAMTS17
EMX2OS
LTBP4
SERINC2
EFR3A
C17orf49
NT5C2
COMTD1
LOC101928737
LOC101928737
TBX5
PAX9
SYNJ2
FAM181A
LINC00595
ITPR1
ZC3H12D
SEMA3B
ETS2
ZC3H12D
SCUBE1
KCP
ADAMTS17
EMX2
LTBP4
SERINC2
EFR3A
RNASEK
NT5C2
COMTD1
TBX5
Methylation change (%)
10
5
0
−5
−10
−50
start
end
50
C
Correlation of methylation difference between array sentinel and targeted methylation site in motif
R=0.63, P=0.001
Targeted sequencing (%)
Array sentinel (%)

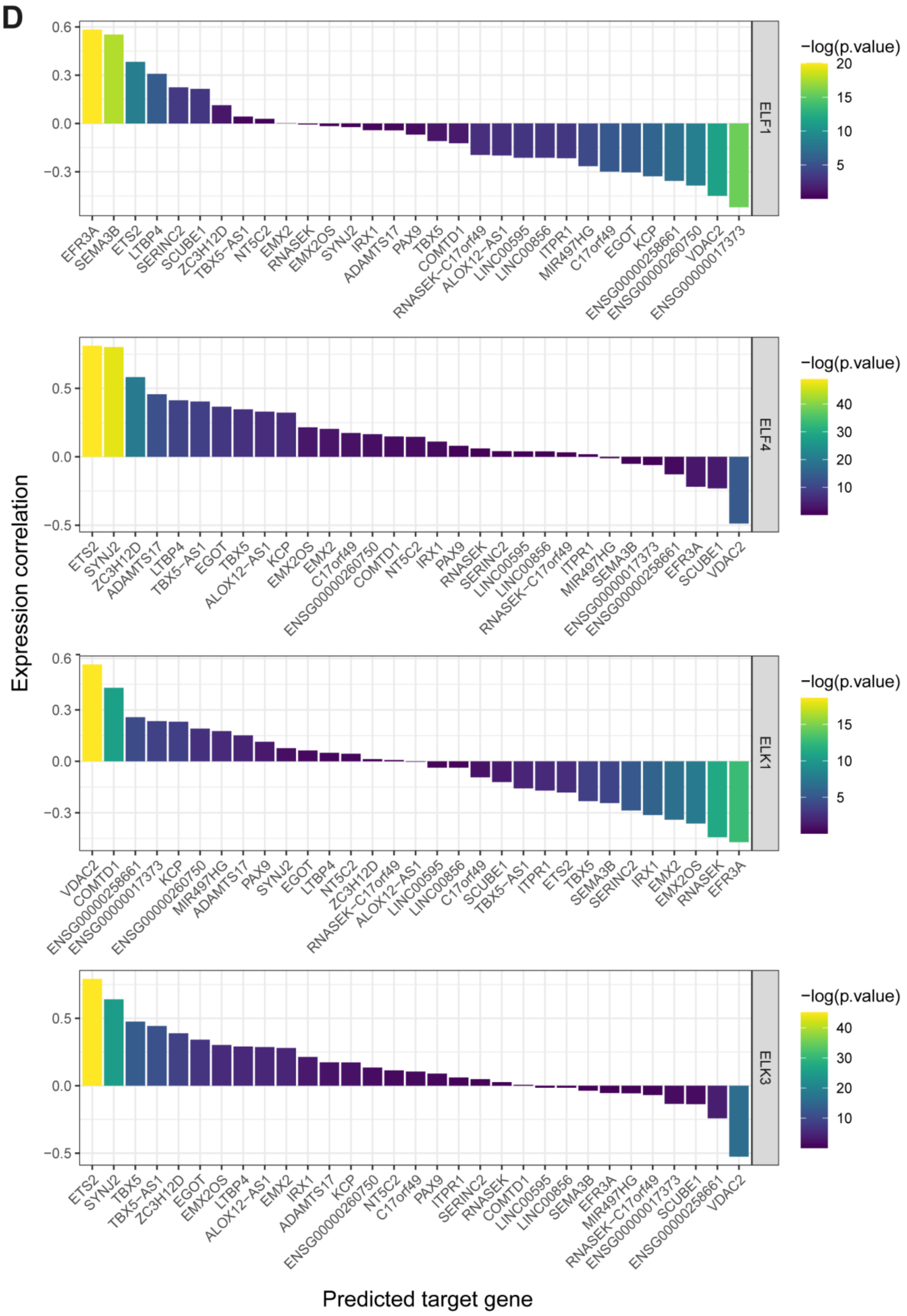

Supplementary Figure 14: TF motif-targeted methylation sequencing analyses.

A. *PRRC2A* locus. Coexpression relationships between (i) TFs predicted to bind at differentially hypomethylated sites associated with obesity, identified through targeted methylation sequencing, at the *PRRC2A* open chromatin region and (ii) *PRRC2A* expression. Presented as Pearson correlation coefficient (R) and coloured by -log10 P value (two-sided). Insets show correlation plots of the top two TFs, *ELK1* and *ESRRA*, and *PRRC2A*. *ELK1* was already linked to Motif 4 but not at the *PRRC2A* locus where the motif was >150-bp from the sentinel site.

B-D. Motif 4 loci. **B.** Heatmap shows methylation sites at each of the N=60 regions linked to Motif 4 (X-axis) ordered by their average methylation change in association with obesity. For each region with a methylation site at positions 3/4 and 7/8 of the Motif (the same position in forward/reverse orientations), the closest and functionally annotated target genes are provided. Density plot presents the sum of the methylation change adjusted for the total number of sequences. Hypomethylated sites associated with obesity were found to be enriched in the Motif, at the CG dinucleotide position, relative to the flanking regions (±50-bp). **C.** Methylation change in association with obesity at targeted methylation sequencing sites located at the CG dinucleotide position in Motif 4 (Y-axis), compared to methylation change at the paired sentinel site (combined array data, X-axis); presented as regression line (blue) and 95% confidence intervals (dark grey), Pearson correlation coefficient (R) and P value (two-sided). **D.** Correlation between (i) expression of 4 TFs predicted to bind at Motif 4 (*ELF1*, *ELF4*, *ELK1* and *ELK3*) and (ii) expression of the predicted target genes of methylation sites located in Motif 4. Presented as Pearson correlation coefficient (R) and coloured by -log10 P value (two-sided).

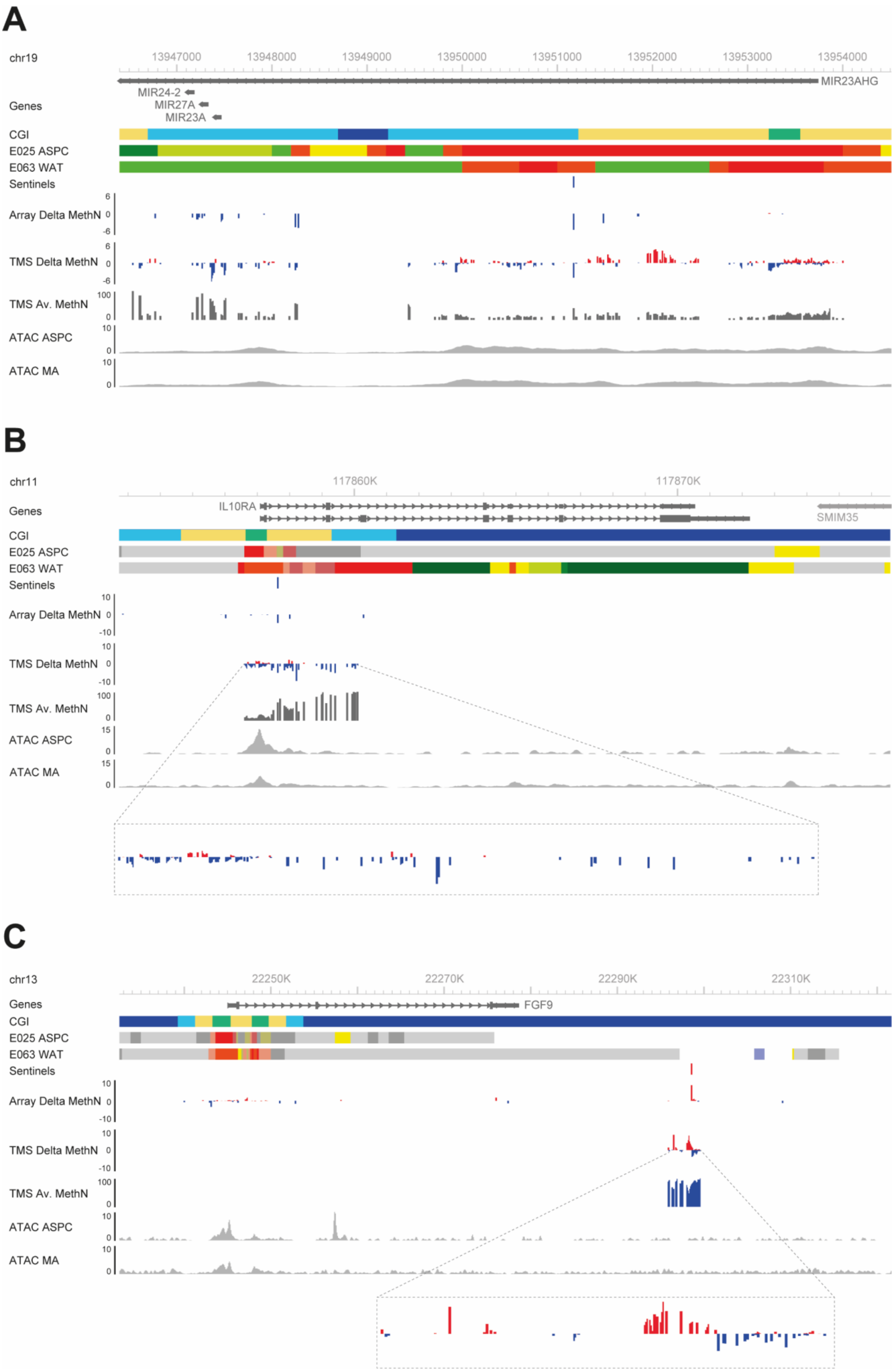
A
chr19
13947000
13948000
13949000
13950000
13951000
13952000
13953000
13954000
MIR23AHG
MIR24-2
MIR27A
MIR23A
Genes
CGI
E025 ASPC
E063 WAT
Sentinels
Array Delta MethN
TMS Delta MethN
TMS Av. MethN
ATAC ASPC
ATAC MA
B
chr11
117860K
117870K
IL10RA
SMIM35
Genes
CGI
E025 ASPC
E063 WAT
Sentinels
Array Delta MethN
TMS Delta MethN
TMS Av. MethN
ATAC ASPC
ATAC MA
C
chr13
22250K
22270K
22290K
22310K
FGF9
Genes
CGI
E025 ASPC
E063 WAT
Sentinels
Array Delta MethN
TMS Delta MethN
TMS Av. MethN
ATAC ASPC
ATAC MA

D

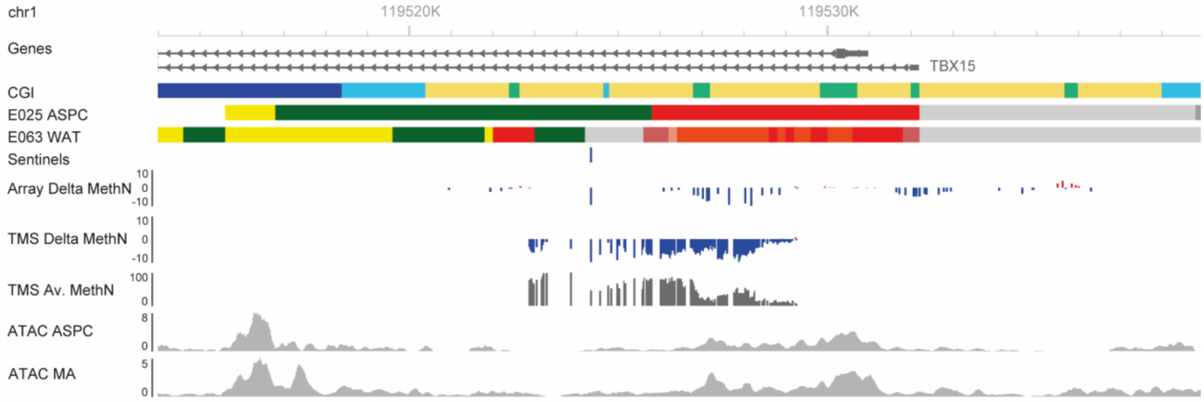
chr1
119520K
119530K
Genes
TBX15
CGI
E025 ASPC
E063 WAT
Sentinels
Array Delta MethN
TMS Delta MethN
TMS Av. MethN
ATAC ASPC
ATAC MA

E

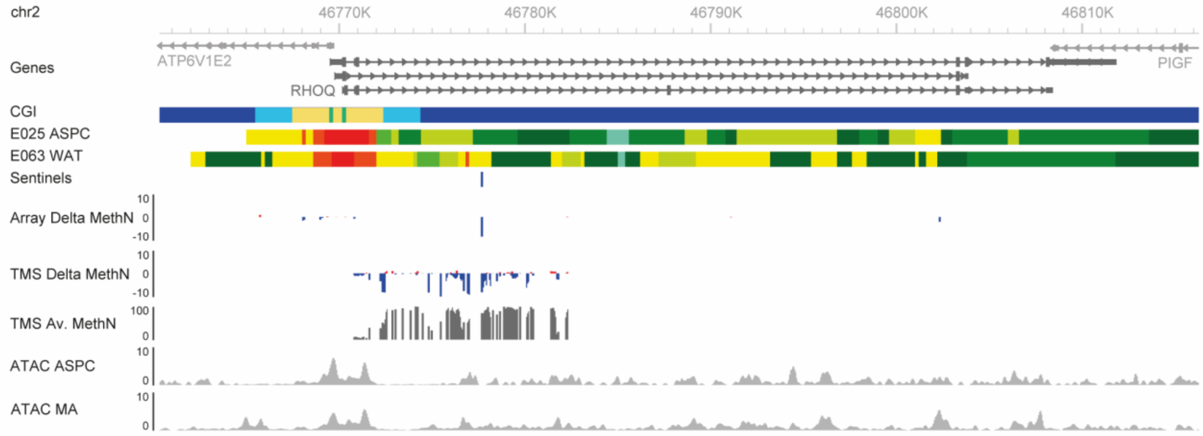
chr2
46770K
46780K
46790K
46800K
46810K
Genes
ATP6V1E2
RHOQ
PIGF
CGI
E025 ASPC
E063 WAT
Sentinels
Array Delta MethN
TMS Delta MethN
TMS Av. MethN
ATAC ASPC
ATAC MA

F

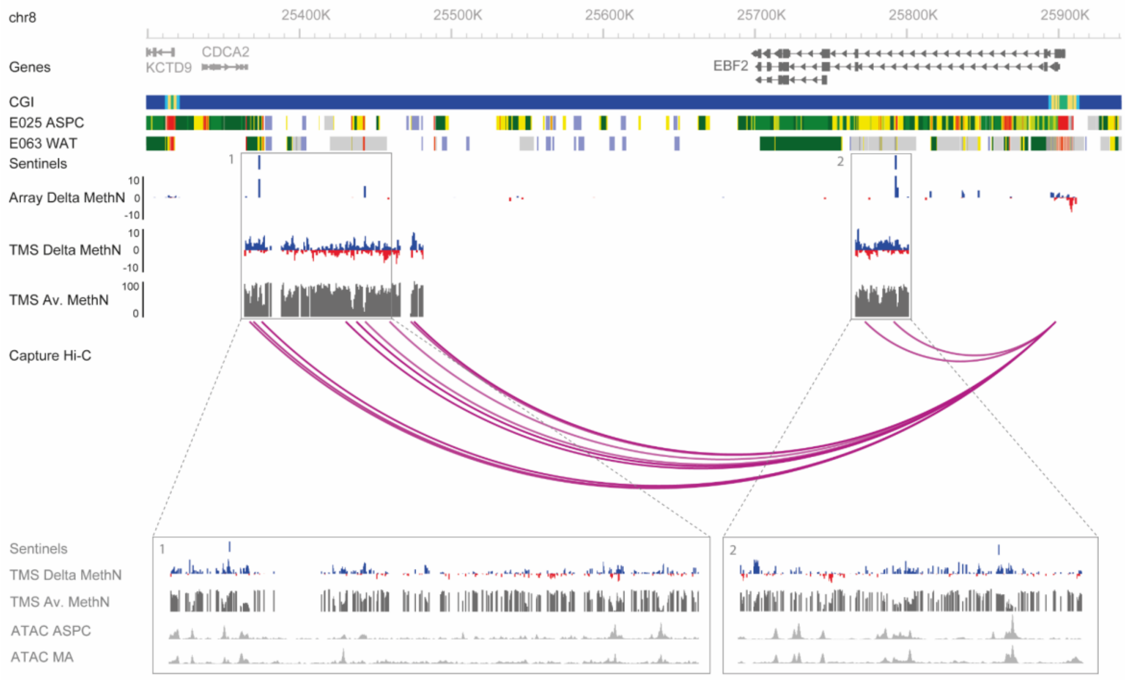
chr8
25400K
25500K
25600K
25700K
25800K
25900K
Genes
CDCA2
KCTD9
EBF2
CGI
E025 ASPC
E063 WAT
Sentinels
Array Delta MethN
TMS Delta MethN
TMS Av. MethN
Capture Hi-C
ATAC ASPC
ATAC MA

G

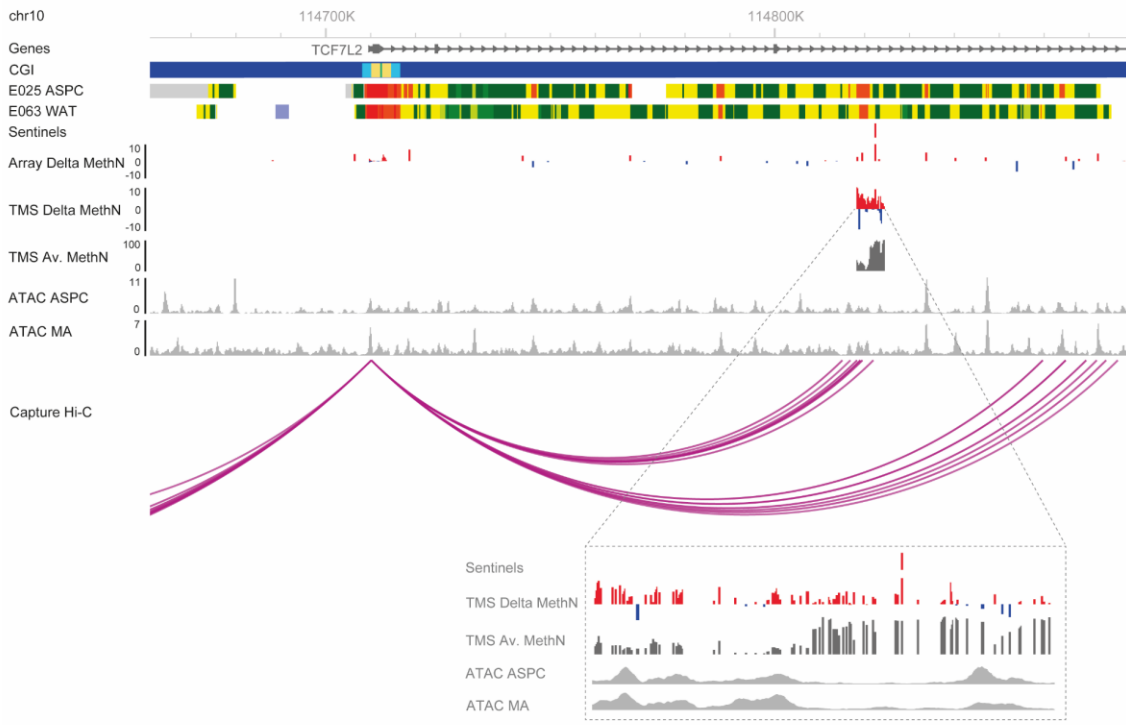
chr10
114700K
114800K
Genes
TCF7L2
CGI
E025 ASPC
E063 WAT
Sentinels
Array Delta MethN
TMS Delta MethN
TMS Av. MethN
ATAC ASPC
ATAC MA
Capture Hi-C

H

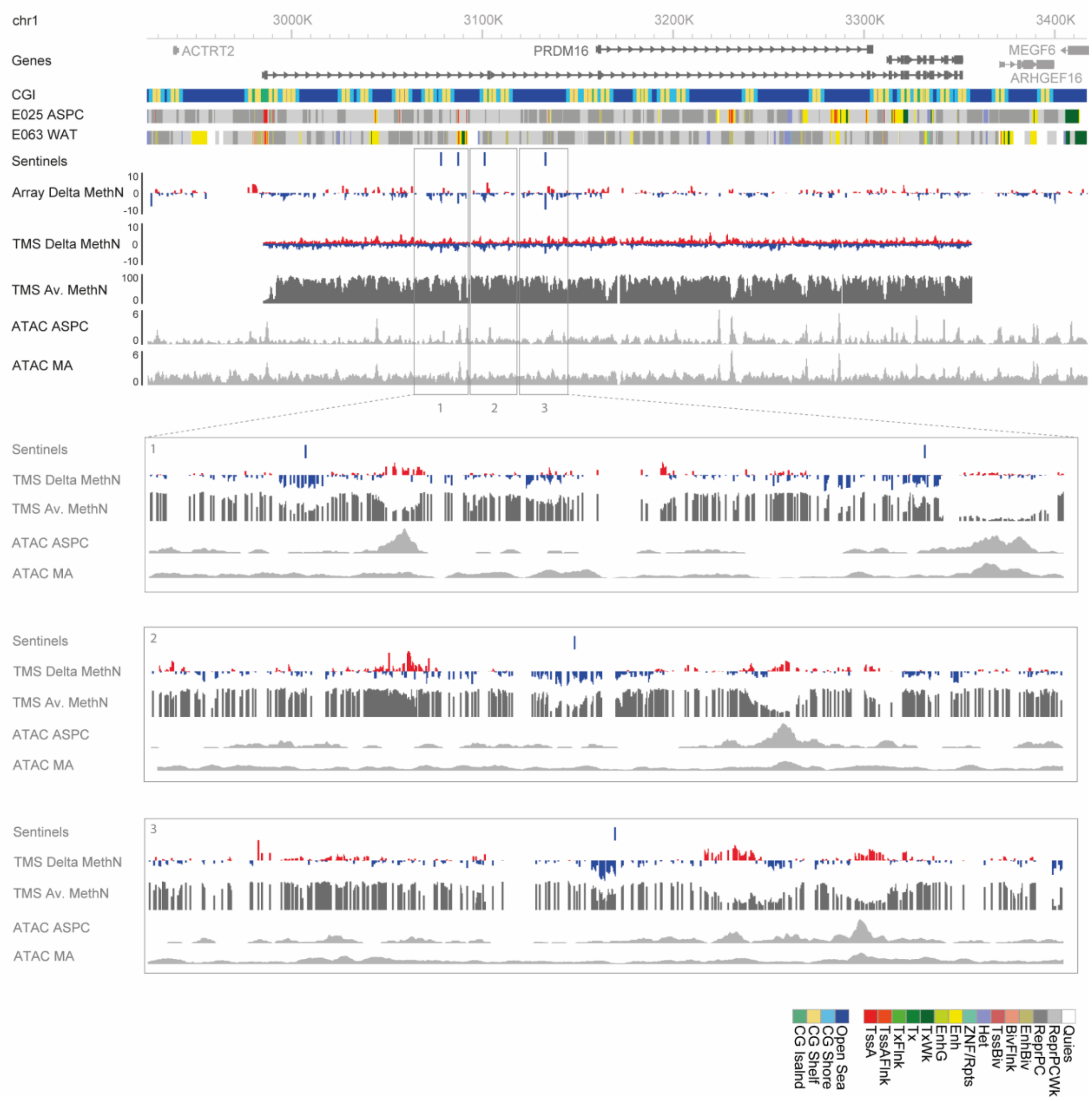

Supplementary Figure 15: Targeted methylation sequencing at other loci of biological interest.

A. *MIR23A*, *MIR24-2*, *MIR27A* cluster. **B.** *IL10RA.* **C.** *FGF9.* **D.** *TBX15.* **E.** *RHOQ.* **F.** *EBF2.* **G.** *TCF7L2.* **H.** *PRDM16.*

CGI: UCSC CpG islands. E063 and E025: Roadmap adipose and adipocyte chromatin states. Sentinel: Sentinel methylation site in combined discovery and replication data. Array Delta MethyN: Difference in methylation (%) in obesity in combined discovery and replication data (red higher, blue lower). TMS Delta MethyN: Difference in methylation (%) in obesity in targeted methylation sequencing data (red higher, blue lower). TMS Av. MethyN: Mean methylation level (%) in targeted methylation sequencing data. ATAC ASPC: ATAC sequencing of undifferentiated SGBS human preadipocytes (GEO: GSE110734[121]). ATAC MA: ATAC sequencing of differentiated SGBS mature human adipocytes (GEO: GSE110734[121]). Capture Hi-C: Human adipocyte promoter capture HiC interaction (GEO: GSE110619[57]).

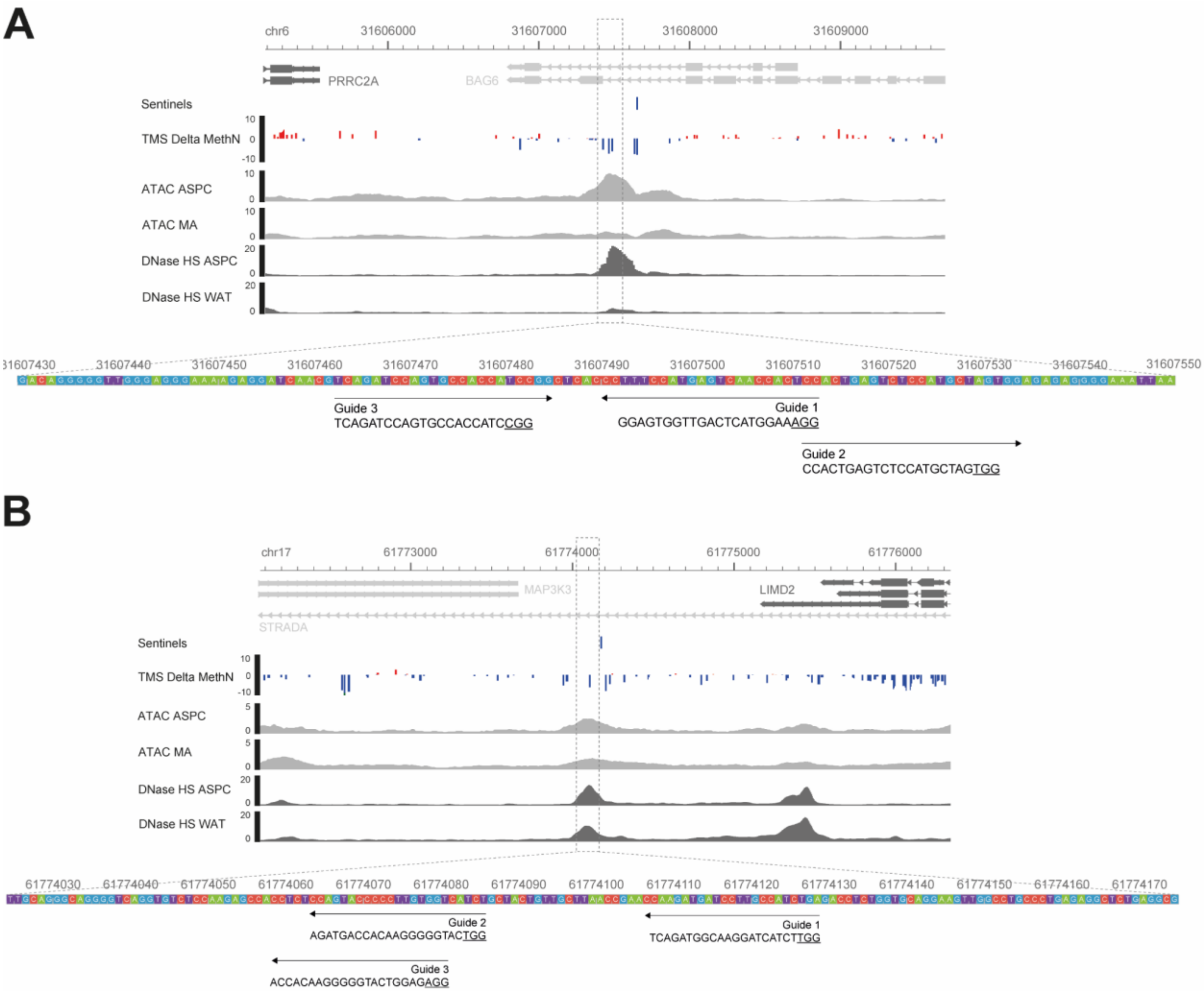

Supplementary Figure 16: CRISPR-activation design.

For each locus, **A.** *PRRC2A* and **B.** *LIMD2*, three guide RNA (gRNA) were designed to target open chromatin peaks at which differentially methylated sites identified by targeted methylation sequencing and flanking the sentinel methylation site were located. Forward genomic DNA sequence and associated gRNA sequences are presented, with PAM sites underlined. Each of the guides was paired together resulting in 3 guide pairs per locus: F1/R2 for Guides 1 and 2; F1/R3 for Guides 1 and 3; F2/R3 for Guides 2 and 3. One of the guide pairs for the *PRRC2A* locus, F1/R3, failed to transduce.

Sentinels: Sentinel methylation site. TMS Delta MethyN: Difference in methylation (%) in obesity in targeted methylation sequencing data (red higher, blue lower). ATAC ASPC: ATAC sequencing of undifferentiated SGBS human preadipocytes (GEO: GSE110734[121]). ATAC MA: ATAC sequencing of differentiated SGBS mature human adipocytes (GEO: GSE110734[121]). DNase HS ASPC: Imputed DNase hypersensitivity site in adipocytes from Roadmap E025. DNase HS WAT: Imputed DNase hypersensitivity site in whole-adipose tissues from Roadmap E063.

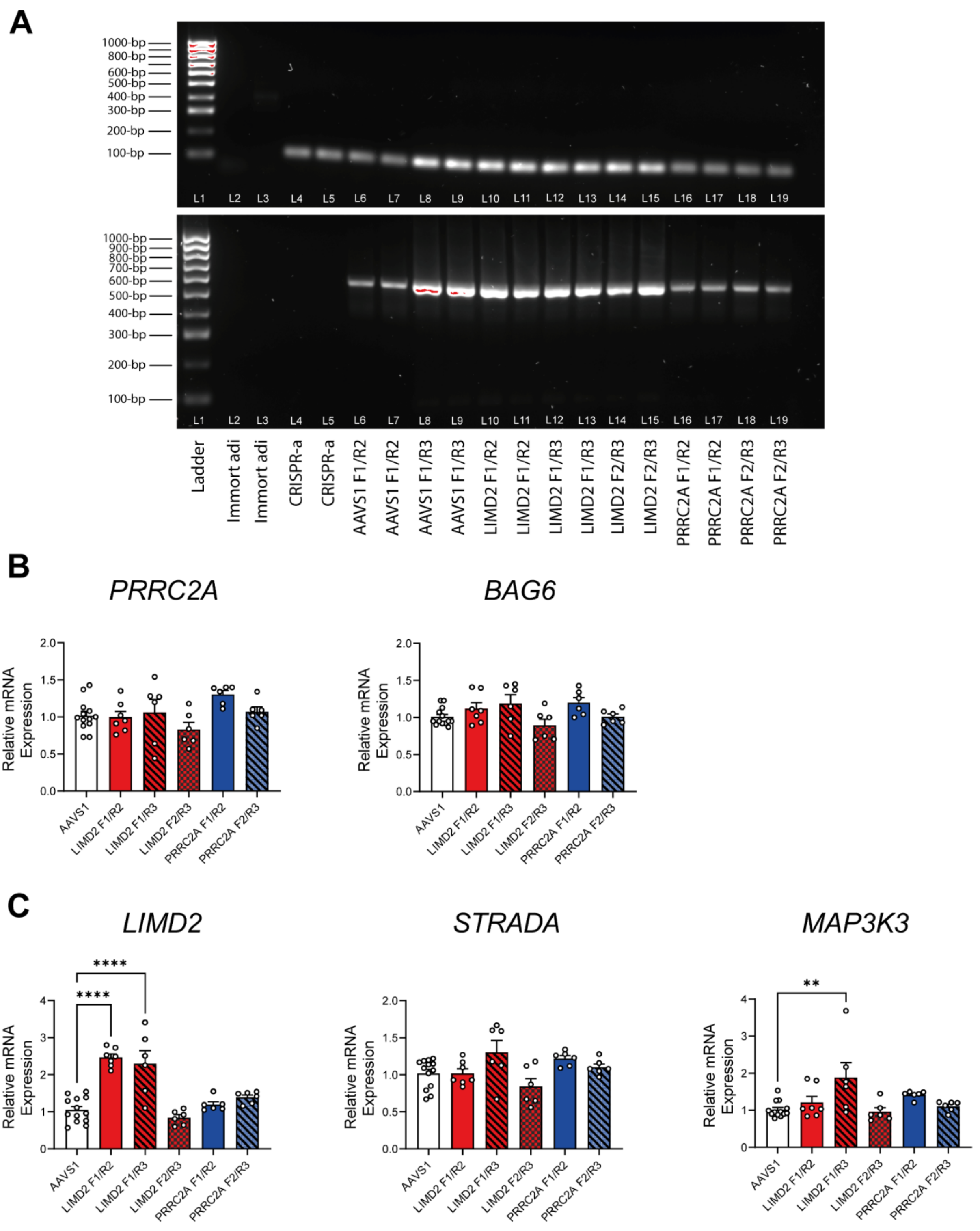

Supplementary Figure 17: CRISPR-activation results for *PRRC2A* and *LIMD2.*

A. Gels confirming successful transduction of the CRISPR-activation (top panel) and guide RNA (bottom panel) vectors (after selection) by amplification of DNA from human adipocyte cell lines: i. without transduction (Immort adi, Lanes 2-3); ii. transduced with activating vector alone (CRISPR-a, Lanes 4-5, Lenti dCAS-VP64); iii. transduced with activating and guide vectors (Lenti dCAS-VP64, LentiGuide-Hygro) containing target gene specific gRNA (*AAVS1* Lanes 6-9, *LIMD2* Lanes 10-15,

PPRC2A Lanes 16-19). Expected sizes of amplification products: Lenti dCAS-VP64 – 105-bp; LentiGuide-Hygro – 571-bp.

B-C. Expression of the predicted target genes of CRISPR-activation, as well as neighbouring genes in the same TAD, in immortalised human adipocytes targeted with gRNA pairs specific to the *PRRC2A, LIMD2* and control *AAVS1* loci (N=6-7/condition). **B.** Targeted activation at the *PRRC2A* locus (using two pairs of guides, F1/R2 N=6 and F2/R3 N=6 independent samples) had no effect on *PRRC2A* expression or expression of the neighbouring gene *BAG6* compared to: i. control *AAVS1* cells; and ii. *LIMD2* gRNA treated cells in which no change in *PRRC2A* would be expected. **C.** Targeted activation at the *LIMD2* locus increased *LIMD2* expression in two (F1/R2 N=7 and F1/R3 N=6 independent samples, P<0.0001) but not a third *LIMD2* cell line (F2/R3 N=6 independent samples) compared to: i. control *AAVS1* cells; and ii. *PRRC2A* gRNA treated cells in which no change in *LIMD2* would be expected. For one *LIMD2* cell line (F1/R3), an increase in expression of the neighbouring *MAP3K3* gene was observed (P=0.0011), though this was explained by an outlying data point. No corresponding increase in the neighbouring *STRADA* gene was observed.

All results are presented as mean ± SEM relative to *AAVS1*, standardised to the housekeeping genes *ACTB* and *GAPDH*. *AAVS1* represents the combined results for the *AAVS1* F1/R2 and F1/R3 guide pairs (N=13 independent samples). *P<0.05, **P<0.01, ***P<0.001, ****P<0.0001 (One-Way ANOVA test, Dunnett's test multiple comparisons); source data are provided in the Source Data file.

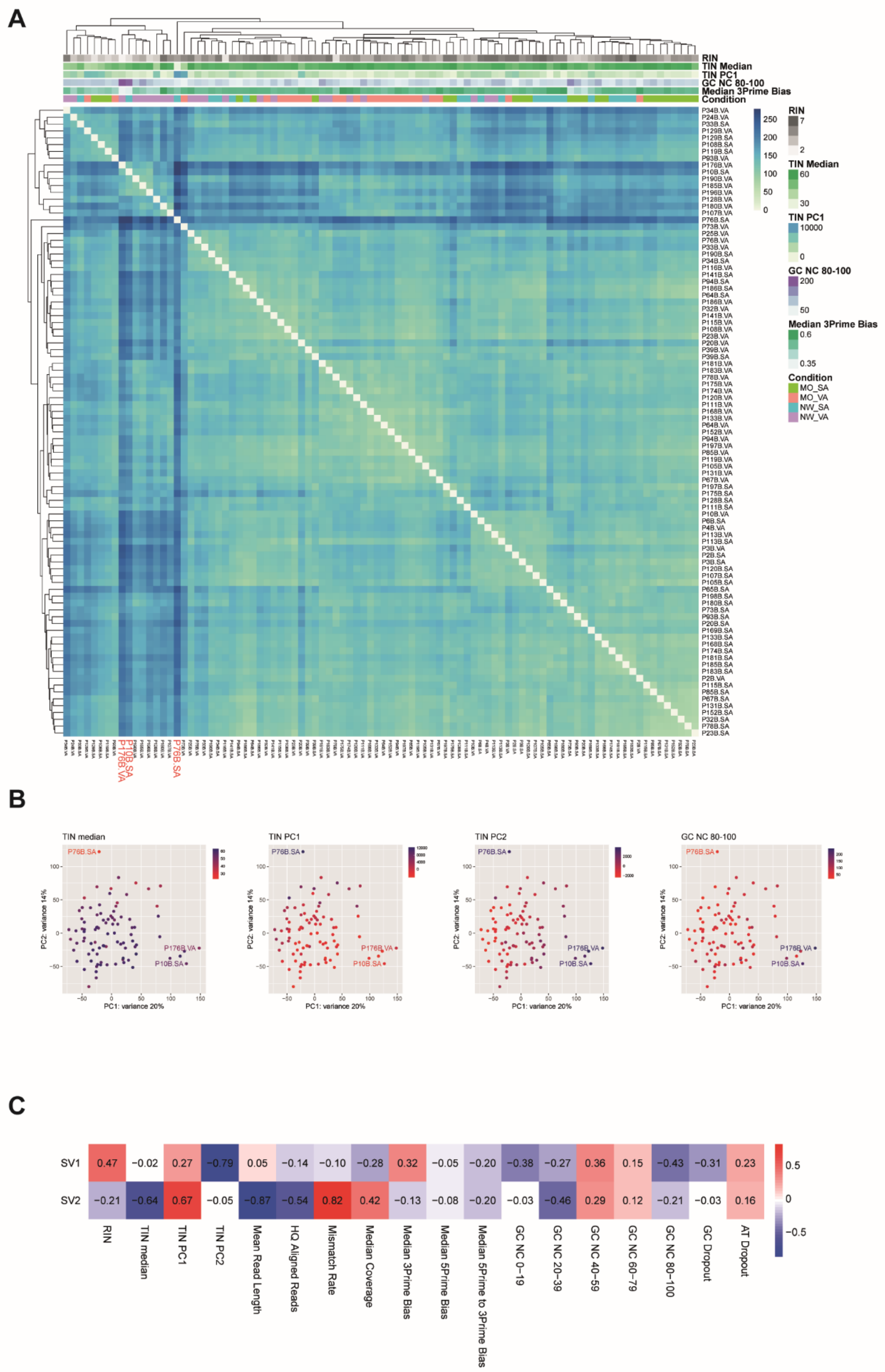

Supplementary Figure 18: Human adipocyte RNA sequencing quality control analyses.

A. Heatmap of between sample distance matrix (euclidean) of whole-transcriptome results (variance stabilising transformed counts, 92 subcutaneous and visceral adipocyte samples with RNA sequencing results at >15M assigned reads). Coloured by RNA integrity (RIN), transcript integrity (TIN Median and PC1), GC bias (GC NC 80-100), coverage bias (Median 3Prime Bias) and sample type. MO_SA: obese subcutaneous. NW_SA: control subcutaneous. MO_VA: obese visceral. NW_VA: control visceral.

B. Principal components 1 and 2 from principal component analysis of whole-transcriptome results (variance stabilising transformed counts, 92 subcutaneous and visceral adipocyte samples). Coloured by sequencing quality control metric: TIN (Median, PC1 and PC2) and GC bias (GC NC 80-100).

C. Surrogate variable analysis of whole-transcriptome results (variance stabilising transformed counts, 92 subcutaneous and visceral adipocyte samples). Pairwise correlation of resulting surrogate variables 1 and 2 with measures of sample quality and sequencing quality (Pearsons): RNA quality (RIN); read and transcript length and quality (TIN Median, PC1 and PC2, Mean Read Length, HQ (high-quality) Aligned Reads, Mismatch Rate); sequencing coverage (Median Coverage, 3Prime, 5Pime, 5Prime to 3Prime Bias) and sequencing GC bias (GC NC 0-100, GC Dropout, AT Dropout).
