# Supplementary material for: Integrative genomic analyses in adipocytes implicate DNA methylation in human obesity and diabetes
Source: Nat Commun. 2023 May 15;14:2784. doi: 10.1038/s41467-023-38439-z (PMC10185556; doi:10.1038/s41467-023-38439-z)
Supplement: Supplementary file 2 — Description of Additional Supplementary Files [file 41467_2023_38439_MOESM2_ESM.pdf]

Description of Additional Supplementary Files

File Name: Supplementary Data 1
Description: **Participant characteristics.** Presented as mean (SD) for continuous variables and proportion for categorical variables. The Student's t-test (two-sided) was used to compare means of continuous variables, and the Chi-squared test to compare frequencies of categorical variables.

File Name: Supplementary Data 2
Description: **Subcutaneous DNA methylation sentinels associated with extreme human obesity in discovery and replication analyses.** Analysed by multivariate linear regression in separate discovery and replication cohorts, and combined by inverse variance weighted meta-analysis.

File Name: Supplementary Data 3
Description: **Visceral DNA methylation sentinels associated with extreme human obesity in discovery and replication analyses.** Analysed by multivariate linear regression in separate discovery and replication cohorts, and combined by inverse variance weighted meta-analysis.

File Name: Supplementary Data 4
Description: **Cross-depot comparisons of significant DNA methylation sentinels associated with extreme obesity.** Analysed by multivariate linear regression in separate discovery and replication cohorts, and combined by inverse variance weighted meta-analysis.

File Name: Supplementary Data 5
Description: **Replication of subcutaneous and visceral DNA methylation sentinels in association with BMI in N=538 whole subcutaneous adipose tissue samples from the Twins UK study.** Greater enrichment of obesity-associated subcutaneous than visceral adipocyte sentinels in whole subcutaneous adipose tissue from MuTHER. concord. = same direction of effect between (i) association with obesity in adipocytes and (ii) association with BMI in adipose tissue, analysed by linear regression; FDR = False Discovery Rate; P<0.05 adj = P<0.05 Bonferroni adjusted; N = number; P_enrich = Binom Test P value (one sided).

File Name: Supplementary Data 6
Description: **Positional overlap of DNA methylation sentinels with human adiposity and metabolic disease GWAS SNPs.**

File Name: Supplementary Data 7
Description: **Associations between genic sentinels and flanking/overlapping cis- target genes in subcutaneous adipocytes.** Analysed by mixed-effects linear regression in combined adipocyte samples from the replication cohort.

File Name: Supplementary Data 8
Description: **Associations between genic sentinels and flanking/overlapping cis- target genes in visceral adipocytes.** Analysed by mixed-effects linear regression in combined adipocyte samples from the replication cohort.

File Name: Supplementary Data 9
Description: **Associations between non-genic sentinels and functionally assigned cis- target genes in subcutaneous adipocytes.** Analysed by mixed-effects linear regression in combined adipocyte samples from the replication cohort.

File Name: Supplementary Data 10
Description: **Associations between non-genic sentinels and functionally assigned cis- target genes in visceral adipocytes.** Analysed by mixed-effects linear regression in combined adipocyte samples from the replication cohort.

File Name: Supplementary Data 11
Description: **Associations between non-genic sentinels and TAD assigned cis- target genes in subcutaneous adipocytes.** Analysed by mixed-effects linear regression in combined adipocyte samples from the replication cohort.

File Name: Supplementary Data 12
Description: **Associations between non-genic sentinels and TAD assigned cis- target genes in visceral adipocytes.** Analysed by mixed-effects linear regression in combined adipocyte samples from the replication cohort.

File Name: Supplementary Data 13
Description: **Depot specific differential expression of cis- target genes in association with obesity among isolated adipocytes and whole adipose tissues.** Depot-specific enrichment of the target genes of DNA methylation sentinels in association with obesity or BMI, using 2 approaches: i. isolated human adipocytes; and ii. whole-adipose tissues from GTEx. Analysed using the negative binomial distribution in DESeq2 (Wald Test, two-sided). Subcut. = subcutaneous; Visc = visceral; FDR = false discovery rate; P<0.05 adj = Bonferroni adjusted P value; concord. = same direction of effect between (i) methylation-expression-obesity associations in adipocytes and (ii) differential expression-obesity; N = number; % = percentage; assoc = associations meeting criteria; same = discovery depot; alt = alternative depot; bgd = depot background; P enrich = binomial test (observed v. background rates, one-sided).

File Name: Supplementary Data 14
Description: **Pathway and gene set analyses of the nearest cis- genes to each subcutaneous and visceral DNA methylation sentinel.** Presented as N genes observed, Mean SEM genes expected in 1000 permutations, fold enrichment of observed / mean expected, and Empirical P value (one-sided).

File Name: Supplementary Data 15
Description: **Pathway and gene set analyses of cis- target genes associated with subcutaneous and visceral methylation sentinels.** Over-representation analysis using the hypergeometric test (one-sided, Benjamini-Hochberg FDR, g:Profiler).

File Name: Supplementary Data 16
Description: **Enriched transcription factor binding motifs within +/-150-bp of the subcutaneous adipocyte DNA methylation sentinels.** Over-representation analysis using the hypergeometric test (one-sided), compared to permuted (Array) and Genomic backgrounds.

File Name: Supplementary Data 17
Description: **Transcription factors predicted to bind at enriched transcription factor binding motifs in subcutaneous adipocytes.**

File Name: Supplementary Data 18
Description: **Putative transcription factor binding motifs within +/-150-bp of the visceral DNA methylation sentinels.** Over-representation analysis using the hypergeometric test (one-sided), compared to permuted (Array) background.

File Name: Supplementary Data 19
Description: **Mendelian Randomisation analyses investigating the causal effects of adipose tissue DNA methylation on human obesity, obesity-induced metabolic disturbances, T2D and lipid traits.** Two Sample MR analyses carried out using the Wald ratio for single SNPs, and inverse-variance weighted (IVW) fixed-effects meta-analysis for multiple SNPs. Directionality evaluated with the Steiger test. Sensitivity analyses used the IVW and MR-Egger methods and included multiple SNPs (clumped at LD $R2>0.8$) adjusting for correlation structure. Heterogeneity measured by Cochran's Q statistic and associated P value.

File Name: Supplementary Data 20
Description: **Replication of human adipose tissue mQTLs with MR evidence of disease causation in human subcutaneous and visceral adipocytes.** Genotype doses were tested for association with change in methylation in subcutaneous and visceral adipocytes separately using linear regression (additive model).

File Name: Supplementary Data 21
Description: **Targeted methylation sequencing regions.**

File Name: Supplementary Data 22
Description: **Targeted methylation sequencing results in subcutaneous and visceral adipocytes.** Analysed by multivariate linear regression separately in subcutaneous (N=43) and visceral (N=46) adipocytes.

File Name: Supplementary Data 23
Description: **TF-*PRCC2A* co-expression association models.**

File Name: Supplementary Data 24
Description: **Primers used in RT-qPCR studies of gene expression in adipocytes.**

File Name: Supplementary Data 25
Description: **CRISPR-activation guide RNA design and verification.**

File Name: Supplementary Data 26
Description: **CRISPR-activation guide RNA and RT-qPCR primers.**
