# Supplementary material for: Integrative genomic analyses in adipocytes implicate DNA methylation in human obesity and diabetes
Source: Nat Commun. 2023 May 15;14:2784. doi: 10.1038/s41467-023-38439-z (PMC10185556; doi:10.1038/s41467-023-38439-z)
Supplement: Supplementary file 4 — Reporting Summary [file 41467_2023_38439_MOESM4_ESM.pdf]

nature portfolio

Corresponding author(s): Dr William Scott
Last updated by author(s): Mar 13, 2023

Reporting Summary

Nature Portfolio wishes to improve the reproducibility of the work that we publish. This form provides structure for consistency and transparency in reporting. For further information on Nature Portfolio policies, see our Editorial Policies and the Editorial Policy Checklist.

Statistics

For all statistical analyses, confirm that the following items are present in the figure legend, table legend, main text, or Methods section.

n/a	Confirmed	
☐	☒	The exact sample size (*n*) for each experimental group/condition, given as a discrete number and unit of measurement
☐	☒	A statement on whether measurements were taken from distinct samples or whether the same sample was measured repeatedly
☐	☒	The statistical test(s) used AND whether they are one- or two-sided<br>*Only common tests should be described solely by name; describe more complex techniques in the Methods section.*
☐	☒	A description of all covariates tested
☐	☒	A description of any assumptions or corrections, such as tests of normality and adjustment for multiple comparisons
☐	☒	A full description of the statistical parameters including central tendency (e.g. means) or other basic estimates (e.g. regression coefficient) AND variation (e.g. standard deviation) or associated estimates of uncertainty (e.g. confidence intervals)
☐	☒	For null hypothesis testing, the test statistic (e.g. *F*, *t*, *r*) with confidence intervals, effect sizes, degrees of freedom and *P* value noted<br>*Give P values as exact values whenever suitable.*
☒	☐	For Bayesian analysis, information on the choice of priors and Markov chain Monte Carlo settings
☒	☐	For hierarchical and complex designs, identification of the appropriate level for tests and full reporting of outcomes
☒	☐	Estimates of effect sizes (e.g. Cohen's *d*, Pearson's *r*), indicating how they were calculated

Our web collection on statistics for biologists contains articles on many of the points above.

Software and code

Policy information about availability of computer code

Data collection	1. Raw genome-wide DNA methylation data acquisition (Illumina HumanMethylation450 and EPIC BeadChips, HiScan) followed by retrieval, background correction, detection, and quantile-normalisation of signal intensities (Minifi 1.36.0 and limma 2.12.0, in R).<br>2. Raw RNA sequencing demultiplex, alignment, counts and quality control (Bcl2Fastq version 2.20, STAR 2.6.0, FeatureCounts 1.6.2, samtools version 1.9, FastQC 0.11.5, MultiQC 1.9, Picard 2.18.12, and RseQC 2.6.4).<br>3. Raw targeted methylation sequencing data (BCLConvert 4.0.3, fastp 0.22.0, bwameth 0.2.5, samtools 1.6, sambamba 0.8.2, Picard 2.6.0, MethylDackel 0.5.1, Methrix 4.2, BISCUIT 1.0.2).
Data analysis	4. Genome wide association between quantile-normalised DNA methylation beta values and extreme human obesity using multivariate regression with adjustment for biological/technical covariates (in R).<br>5. Association between quantile-normalised DNA methylation beta values and transformed cis-gene expression counts (variancePartition 1.18.0: voomWithDreamWeights, DESeq2 1.28.0) using multivariate regression with adjustment for biological/technical covariates (variancePartition 1.18.0: DREAM, and nlme 3.1-149).<br>6. Differential expression analyses in adipocytes (DESeq2 1.28.0).<br>7. Functional enrichment analyses comparing observed sentinel methylation sites with expected background (based on 1000 null permuted DNA methylation datasets) in R.<br>8. Transcription factor binding site enrichment analyses of observed sentinel DNA methylation sites compared to permuted and genomic background (using Homer 4.11.1: fingMotifGenome.pl and annotatePeaks.pl, seqPattern 1.20.0, TFBSTools 1.36.0, and nlme 3.1-149).<br>9. Mendelian Randomisation (MR) analyses of genotype-methylation-phenotype relationships (using TwoSampleMR 0.5.1 and MendelianRandomization 0.4.1, in R); adipocyte genetic variant studies were done in IMPUTE2 and PLINK2.0; adipose mQTL analyses used MatrixEQTL 2.1.0.<br>10. Association between DNA methylation values from targeted sequencing and extreme human obesity using multivariate regression with

adjustment for biological/technical covariates (Methrix 4.2 in R).
11. Custom scripts for generating association, functional enrichment and MR results are available from Github: https://github.com/WRScottImperial/Human-adipocyte-5mC-obesity.
12. False discovery rates were applied using qvalue 2.20.0 in R.

For manuscripts utilizing custom algorithms or software that are central to the research but not yet described in published literature, software must be made available to editors and reviewers. We strongly encourage code deposition in a community repository (e.g. GitHub). See the Nature Portfolio guidelines for submitting code & software for further information.

Data

Policy information about availability of data

All manuscripts must include a data availability statement. This statement should provide the following information, where applicable:

- Accession codes, unique identifiers, or web links for publicly available datasets
- A description of any restrictions on data availability
- For clinical datasets or third party data, please ensure that the statement adheres to our policy

Genome-wide DNA methylation data from the discovery and replication cohorts are deposited in the Gene Expression Omnibus (GEO accession No: GSE222595, https://www.ncbi.nlm.nih.gov/geo/query/acc.cgi?acc=GSE222595). Raw RNA sequencing data from the replication cohort are available in the European Genome-Phenome Archive (EGA study no. EGAS00001007118, https://ega-archive.org/studies/EGAS00001007118) to provide managed open access to genetic variant containing data. Associated participant characteristics are provided for both methylation and RNA sequencing datasets. Methylation bedGraph files of the methylation differences in obese compared to lean subcutaneous and visceral adipocytes (combined discovery and replication cohort) are also available at GEO (accession No: GSE222595, https://www.ncbi.nlm.nih.gov/geo/query/acc.cgi?acc=GSE222595). The following publicly available datasets were used in this study: GeneHancer (https://www.genecards.org/), Human adipocyte Capture Hi-C (https://www.ncbi.nlm.nih.gov/geo/query/acc.cgi?acc=GSE110619), Human adipocyte TADs (https://www.ncbi.nlm.nih.gov/geo/query/acc.cgi?acc=GSE109924), Human adipocyte ATAC (https://www.ncbi.nlm.nih.gov/geo/query/acc.cgi?acc=GSE110734), Roadmap Epigenomes (https://egg2.wustl.edu/roadmap/web_portal/), JASPAR2022 (https://jaspar.genereg.net/) and the Molecular Signatures Database (MsigDB https://www.gsea-msigdb.org/gsea/msigdb/). Human GWAS summary statistics were obtained from: GIANT (https://portals.broadinstitute.org/collaboration/giant/index.php/GIANT_consortium_data_files), MAGIC (https://magicinvestigators.org/downloads/), DIAGRAM (https://diagram-consortium.org/downloads.html), and the Global Lipids Genetics Consortium (https://csg.sph.umich.edu/willer/public/glgc-lipids2021/). Source data for adipocyte functional studies are provided with this paper.

Human research participants

Policy information about studies involving human research participants and Sex and Gender in Research.

Reporting on sex and gender	Study findings relate to both sexes combined (self reported), although the majority of study participants were female (>75%) due to the demographics of people with obesity undergoing bariatric weight loss surgery, and the need to match controls with cases. Sex-specific analyses were not performed because of the limited sample sizes, and consequent (i) lack of power to evaluate sex-specific effects and (ii) risk of over-fitting relationships in males.
Population characteristics	Obese and healthy control study participants were matched for age, sex and ethnicity (within 3-yrs) in the discovery and replication cohorts. Overall, the combined cohorts were >75% female, age range 20-70-yrs.
Recruitment	Obese cases and healthy controls were recruited sequentially from bariatric and other pre-operative clinics at University College London Hospital. People with type-2 diabetes on medication were excluded as were people with systemic illnesses not related to obesity. Obese study participants were then selected at random from among the larger recruited cohort to minimise selection bias; control study participants were chosen to match to each obese participant (age within 3-yrs, sex, ethnicity). The majority of participants were female due to the demographics of people undergoing bariatric surgery. We are unable to test for and thus rule out female sex-specific effects due to our sample size.
Ethics oversight	The study was approved by the London – City Road and Hampstead Research Ethics Committee, United Kingdom (reference 13/LO/0477).

Note that full information on the approval of the study protocol must also be provided in the manuscript.

Field-specific reporting

Please select the one below that is the best fit for your research. If you are not sure, read the appropriate sections before making your selection.

☒ Life sciences ☐ Behavioural & social sciences ☐ Ecological, evolutionary & environmental sciences

For a reference copy of the document with all sections, see nature.com/documents/nr-reporting-summary-flat.pdf

Life sciences study design

All studies must disclose on these points even when the disclosure is negative.

Sample size	Sample size estimates were made using observed associations between DNA methylation and BMI in blood from our previously published

Sample size	studies (https://doi.org/10.1038/nature20784, in GPower 3.1). Based on our extreme trait design we expect >95% power to detect a >4% difference in DNA methylation between cases and controls in combined discovery and replication analyses.
Data exclusions	Two DNA methylation samples that failed quality control were excluded. 5 RNA sequencing samples that failed quality control or represented extreme technical outliers were excluded.
Replication	Genome-wide DNA methylation findings in adipocytes were replicated in independent discovery (N=96) and replication (N=96) cohorts. Human adipocyte findings were further replicated in an independent cohort of whole adipose tissue samples (TwinsUK, N=538). Adipocyte cell studies were repeated (at least two times) to ensure reproducibility; attempts at replication were successful.
Randomization	Human adipocyte samples were selected from a larger cohort of obese cases and controls, matching for age, sex and ethnicity (<3-yrs). Genome wide-DNA methylation and RNA sequencing assays were carried out in single batches. Sample order for genomic library preparation and BeadChip/sequencing assays was randomised. Biological and technical covariates were included in regression models examining methylation, expression and phenotype relationships.
Blinding	Non formal blinding was considered necessary as samples/genomic libraries were anonymised and processed in a randomised order.

Reporting for specific materials, systems and methods

We require information from authors about some types of materials, experimental systems and methods used in many studies. Here, indicate whether each material, system or method listed is relevant to your study. If you are not sure if a list item applies to your research, read the appropriate section before selecting a response.

Materials & experimental systems

n/a	Involved in the study
☒	☐ Antibodies
☐	☒ Eukaryotic cell lines
☒	☐ Palaeontology and archaeology
☒	☐ Animals and other organisms
☒	☐ Clinical data
☒	☐ Dual use research of concern

Methods

n/a	Involved in the study
☒	☐ ChIP-seq
☒	☐ Flow cytometry
☒	☐ MRI-based neuroimaging

Eukaryotic cell lines

Policy information about cell lines and Sex and Gender in Research

Cell line source(s)	3T3-L1 adipocytes from ATCC LGC UK, Immortalised human adipocytes from abm, HEK293T from the European Collection of Authenticated Cell Cultures.
Authentication	The cells used in the study were tested for adipocyte differentiation capacity and adipocyte specific marker genes.
Mycoplasma contamination	The cells used in the study were tested to confirm the abscence of Mycoplasma infection.
Commonly misidentified lines (See ICLAC register)	No commonly misidentified cell lines used.
